# Fast Phenotypic and Genetic Changes in Life History and Mortality of Experimental Populations of Guppies (*Poecilia reticulata*) Exposed to Size‐Dependent Harvest in a Decade‐Long Selection Experiment

**DOI:** 10.1111/eva.70253

**Published:** 2026-05-18

**Authors:** B. Diaz Pauli, E. D. Mach, H. Savolainen, A. C. Utne‐Palm, V. Bartuseviciute, D. Reznick, M. Heino

**Affiliations:** ^1^ Department of Biological Sciences University of Bergen Bergen Norway; ^2^ Institute of Marine Research Bergen Norway; ^3^ Department of Evolution, Ecology and Organismal Biology University of California Riverside California USA; ^4^ Complexity Science and Evolution Unit Okinawa Institute of Science and Technology Graduate University (OIST) Okinawa Japan

**Keywords:** anthropogenic impact, fisheries‐induced evolution, haldanes, life history, natural mortality, senescence

## Abstract

Anthropogenic stressors on wild populations commonly impact large individuals more strongly than small ones. A particularly intense mortality on large individuals occurs during harvesting (fishing and hunting). Strong harvest‐induced, positively size‐dependent mortality leads to smaller size and earlier maturation. However, its effects on other fitness components are yet unclear, as different trade‐offs are at play (e.g., current vs. future reproduction, growth vs. reproduction, growth vs. survival). Here we present a unique decade‐long size‐dependent selection experiment to study the interplay of trade‐offs among life‐history traits and the relative importance of harvest‐induced and natural selective pressures using guppies, *Poecilia reticulata,* as model species. By allowing iteroparous life history in our populations, we showed that populations exposed to positively size‐dependent harvest, as that imposed by fisheries, evolved towards faster life history (i.e., heritable changes towards early maturation and high fecundity) and higher “natural” mortality in laboratory conditions, despite an increased lifespan. Our results on length and maturation match those of earlier artificial selection experiments (modelling semelparous life histories), but the different experimental designs resulted in opposite conclusions about fecundity and juvenile growth. Our experimental design, allowing intertemporal trade‐offs to unfold naturally under dynamic demographic environments, overcomes the limitations of earlier studies that did not permit these trade‐offs. Our unique study, lasting 10 years and assessing over 14 life‐history traits, evidences the importance of sufficiently realistic experimental set‐ups to better understand the impacts of anthropogenic stressors. Future research should investigate the ecosystem consequences of these life‐history changes to further understand the impact of size‐dependent harvest on fish populations.

## 1 Introduction

The effects of human‐induced stressors, such as climate change, harvesting, habitat loss, and introduced species, on wild animals are body‐size dependent, with large individuals being more impacted compared to the small ones (Cardillo et al. 2005; Heino et al. 2015; Ahti et al. 2020; Verberk et al. 2021; Kopf et al. 2024). Harvesting, in particular fishing and hunting, has an especially strong impact on size, as large individuals are commonly targeted because of their high economic value (Heino et al. 2015; Worm 2015; Kuparinen and Festa‐Bianchet 2017). Additionally, the extraction rate associated with such harvesting is particularly intensive relative to natural predation (Darimont et al. 2015). Together, selectivity and intensity of harvesting make it a stressor with a strong impact on population demography.

Intense fishing‐induced mortality has not only resulted in large population declines (Rousseau et al. 2019) but also in important shifts in life‐history traits, such as earlier maturation (Sharpe and Hendry 2009; Heino et al. 2015). Three different processes could contribute toward such phenotypic changes: (1) removal of older and larger fish shifts a population's age and size distribution toward younger and smaller fish as a direct demographic response (*Baranov's fishing up effect*; Baranov 1918), (2) reduction in biomass reduces intra‐specific competition and results in higher per‐capita food availability, thus allowing for faster growth and earlier maturation as a phenotypically plastic response (*compensatory response*; Law 2000), and (3) increased mortality favours individuals with genotypes that allow earlier maturation at smaller sizes as an evolutionary response (*fisheries‐induced evolution*). Adaptation through earlier maturation is predicted even if fishing impacted all sizes equally because increased mortality reduces expected future reproductive success (Stearns 1992). Fishing that targets larger individuals makes selection towards faster life histories even stronger. While these general principles of fisheries‐induced adaptation are widely recognized and understood, there are many dimensions that are still poorly known. The most urgent need is understanding the harvest effects on trade‐offs between the traits under direct selection and other fitness components and characterizing the ecological consequences of the observed trait changes at the population and ecosystem levels (Kuparinen and Festa‐Bianchet 2017).

Strong positively size‐dependent mortality is known to favour individuals with smaller size and earlier maturation; however, its effects on other fitness components are yet unclear. An immediate cost is the reduced absolute fecundity usually associated with smaller body size (number or quality of offspring; Walsh et al. 2006; Uusi‐Heikkilä et al. 2015). A more complex set of trade‐offs originates from the trade‐off between current and future reproduction. Evolutionary theory predicts that in age‐structured populations, reproductive investments should increase when mortality increases (Charlesworth 1994; Montiglio et al. 2018a), in agreement with the observed higher gonadosomatic index in a few studies in the wild (guppy: Reznick, Rodd, and Cardenas 1996; Reznick et al. 1997; cod: Heino et al. 2015) – but recall that reproductive investment does not only refer to production of eggs (as originally presented in the classical theory), but also includes investment in secondary sexual traits and sexual behaviours (courting, migration, etc.; Heino et al. 2015). Thus, an increased reproductive investment may imply a survival cost, as mating and behaviours including those to ensure resources for reproduction might involve higher predation risk. Moreover, in fish, carrying larger gonads may lead to indirect fitness costs through lower swimming performance (Jørgensen and Holt 2013; Montiglio et al. 2018b), but see (Olsen et al. 2023).

Size‐dependent mortality effects on juvenile and adult growth are complex (Enberg et al. 2012; Heino et al. 2015). According to life‐history theory, harvest‐induced size‐dependent mortality should generally lead to slower adult growth, as there is a trade‐off between somatic growth and reproductive investment. In contrast, whether faster or slower juvenile growth is expected depends on how size‐dependent harvest mortality interacts with growth and maturation, e.g., whether the harvest size‐limit is set bellow or above the typical maturation size (Dunlop et al. 2009). However, the compensatory effect of fishing predicts phenotypic growth rate to increase because of reduced population density (Law 2000). Finally, growth also trades off with survival. Fast growing individuals might invest less energy in basal metabolism or present more risky behaviours (Dunlop et al. 2009; Enberg et al. 2012). The relative importance of all these trade‐offs and on which sizes mortality predominantly occurs would lead to different responses in maturation schedules. Mortality on medium‐sized or adult individuals can favour early or late maturation depending on whether mortality costs exceed growth costs (Poos et al. 2011). Such interplay between trade‐off and size‐dependent mortality may only become apparent in iteroparous life histories that face trade‐offs between current and future investments (Poos et al. 2011; Diaz Pauli and Heino 2014).

Studying the interplay of trade‐offs among life‐history traits, as well as understanding the relative importance of harvest‐induced and natural selective pressures is challenging. Selection experiments are a useful tool to study aspects of size‐dependent mortality that are out of reach for field studies (Diaz Pauli and Heino 2014). These types of experiments have proven that positively size‐dependent mortality has direct effects on life histories within short time‐scales: markedly smaller adult body size can be reached within 3–6 generations (Conover and Munch 2002; Amaral and Johnston 2012; van Wijk et al. 2013; Uusi‐Heikkilä et al. 2015; Renneville et al. 2020; Wootton et al. 2021; Xi et al. 2023), along with earlier maturation (van Wijk et al. 2013; Xi et al. 2023), changes in reproductive investment (Walsh et al. 2006; Uusi‐Heikkilä et al. 2015; Sbragaglia et al. 2019), and behaviour (Walsh et al. 2006; Uusi‐Heikkilä et al. 2015; Diaz Pauli et al. 2019; Sbragaglia et al. 2021; Roy et al. 2023). Finally, some of the selection experiments found genetic differences linked to size‐dependent mortality (van Wijk et al. 2013; Uusi‐Heikkilä et al. 2015; Therkildsen et al. 2019; Sun et al. 2025). However, all these experiments involved artificial selection performed on semelparous species or forcing semelparity on naturally iteroparous species through discrete‐generations experimental designs. While such designs are worthy in many ways, they inhibit the trade‐offs between current and future investments from unfolding as they would in iteroparous populations (Diaz Pauli and Heino 2014). Studies with natural predation in the wild have shown analogous results in iteroparous life histories—evolutionary change towards delayed maturation in response to released predation (opposite to increased harvest; Reznick and Travis 2019). This led to higher population density and lower food availability, and eventually, the evolution of delayed maturity (Travis et al. 2023).

Here we present a size‐dependent harvesting experiment featuring guppies ( *Poecilia reticulata* ) as the model species. Three experimental treatments were created with three different harvest regimes: (1) positively size‐dependent harvest (hereafter “Positive harvest”) where only individuals above a minimum size were removed from the population, (2) negatively size‐dependent harvest (“Negative harvest”) where only individuals *under* the aforementioned minimum size were removed, and (3) size‐independent harvest (“Random harvest”) where both large and small individuals were removed. Positive harvest mimics the typical size‐dependent mortality imposed by fishing and is the foremost treatment of the study. Negative harvest and Random harvest provide the necessary contrast and can even be interpreted as kinds of control treatments, acknowledging that a “true”, selectively neutral control treatment would not be possible to realize. Random harvest is unselective in size, but the imposed mortality is still evolutionary selective, as it is different from natural mortality regime and favours fast life histories, like Positive harvest, but at a lower intensity. Negative harvest mimics wild conditions where juveniles experience higher mortality than adults. In addition, Random and Negative harvest could approximate two proposed harvesting regimes, balanced harvest (Garcia et al. 2012) and longevity conservation (Kopf et al. 2024), respectively. Our harvest rates are within or below what wild populations are likely to experience (Reznick, Butler, et al. 1996), and are also in the range seen in commercial fisheries if we liken our 6‐week harvest cycles to annual cycles in the wild (e.g., Olsen and Moland 2011). The selection experiment occurred over a decade, during which the populations experienced two phases of size‐dependent harvest and one phase of recovery, where all lines were exposed to Random harvest. The aim of the recovery phase was assessing whether life‐history traits would recover to initial conditions. On six occasions throughout the experiment, all populations were sampled, and 2‐generation‐common garden experiments were carried out to reveal any heritable response to harvest, as differences that persist after two generations are assumed to have a genetic basis (Reznick and Travis 2019). We compared the change over time among size‐dependent harvest regimes in the following traits and metrics: size, growth, maturation, fecundity, natural mortality, and generation time. We also considered two metrics of size commonly used in fisheries science: length above the minimum size limit, and length at which 50% of the individuals are mature.

Our study aims to fill gaps in current knowledge by overcoming limitations discussed above. Thus, we conducted a laboratory natural selection experiment (see glossary; sensu Garland 2003; Diaz Pauli and Heino 2014) designed to allow the fish to express their natural, iteroparous life history (Diaz Pauli and Heino 2014). The guppies lived in mixed‐age populations with overlapping generations. Density‐dependent processes (including cannibalism) as well as “natural” and sexual selection were allowed to operate. With these premises, our selection experiment represents higher ecological realism than any other experiment before (but see Bouffet‐Halle et al. 2021). Our aim was to assess the multi‐trait response to positively size‐dependent harvest in the presented laboratory setting, comparing the changes with the initial conditions and between harvest regimes to determine (1) the key driver of the changes (harvest regime, change in biomass, or acclimatation to lab conditions), and (2) the nature of changes, that is, whether they are purely plastic or also heritable and thus they are due mainly to compensatory responses or also to fisheries‐induced evolution. In addition, we compare our results to earlier studies with lesser ecological realism. We expected populations where large individuals were harvested to evolve towards fast life history with smaller body length, earlier maturation at smaller length, higher fecundity, and higher natural mortality rate, and hence shorter lifespans.

## 2 Material and Methods

### 2.1 Timeline of the Experiment

**FIGURE 1:**
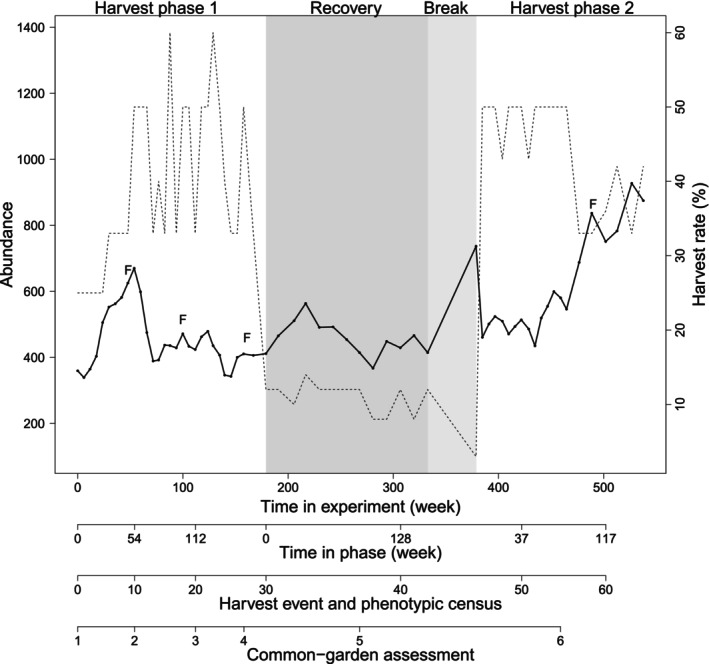
Change in average abundance from all nine populations (solid line) and harvest proportion (dotted line) during the timeline in the selection experiment (first *x*‐axis). Different harvest phases are shown with white and shaded areas, and time within each phase in second *x*‐axis. Harvest events, phenotypic censuses and common‐garden assessments are shown in third and fourth *x*‐axis, respectively. “F” indicates the four times that extra females were sampled to assess fecundity.

Nine replicate populations were created in March 2010 with 140 F1‐offspring of wild‐caught parents and maintained in 400‐l aquaria (See Appendix S1: section 2.1 for details). Each population was initiated with an even sex ratio and an equal contribution from each wild female as feasible. In October 2010, when the replicate populations had over 200 individuals and showed broad age and size distributions, we started the size‐dependent harvest. At the onset of harvest, the populations had an average total abundance of 360 ± 88 individuals and a mature abundance of 193 ± 94 individuals (Table S1, Appendix S1: section 2.1). Here we include data compiled until February 2021, covering 520 weeks (10 years). Throughout the experiment, population abundance averaged 506 ± 190 individuals and ranged from 100 to 1384 individuals (Figure 1, Figure S1).

The selection experiment was divided into three phases: (1) harvest phase 1, where the populations were harvested every 6 weeks following the treatment‐specific size‐dependent regime (see below) and lasted 167 weeks, (2) recovery phase, where all populations experienced Random harvest every 12 weeks for a total duration of 154 weeks, followed by 46 weeks where there was no harvest at all, and (3) harvest phase 2, which was similar to harvest phase 1 and lasted 153 weeks. During harvest phase 2, the last six harvesting events occurred at 12‐week intervals; in these events, the harvest intensity was doubled to maintain intensity comparable to the 6‐week intervals (Figure 1). Food was rationed and equal for all populations, as the aim was to have a compensatory response in the populations. Food ratio was not constant throughout the experiment but adapted to the average abundance and biomass of all populations to maintain animal welfare. For more details see Diaz Pauli (2012) and Appendix S1.

### 2.2 Size‐Dependent Harvest Regimes

The three different harvest regimes, each replicated three times, were as follows: (1) *Positive harvest* was positively size‐dependent and consisted of removing a proportion *P* of the individuals larger than 16 mm standard length (SL, measured from the tip of the snout until the insertion of the caudal fin in the peduncle), assuming equal numbers of small and large fish; this would correspond to harvest proportion *P*/2 at the population level, (2) *Negative harvest*, with negatively size‐dependent harvest, removed *P* of the individuals smaller than 16 mm SL (approximately *P*/2 of the whole populations), and (3) *Random harvest; P*/2 of all the individuals were removed, irrespectively of size. The size threshold for harvesting was chosen because males and females in the founding population begin maturing typically around 16 mm SL. Harvest intensity *P* was modified throughout the experiment, depending on the population abundance and harvest interval. It started out low (*p* = 25%) when the populations were still small, and it was increased when a steady increase in abundance was observed, or decreased when a steady decrease in abundance occurred (Figure 1). On average, the harvest intensity was 40%, with a maximum intensity of 60% and a minimum of 25%, which corresponds to a mortality of about 12.5%–30% of the whole population. This is comparable to harvest mortality experienced by Atlantic cod, *Gadus morhua* , from the coast of Skagerrak, Norway (25%–50% in 1 year Olsen and Moland 2011), if we liken our 6‐week harvest cycles to annual cycles in the wild. See Appendix S1: section 1.1 for detailed methods.

### 2.3 Life‐History and Population Assessments

We performed two types of life‐history and population assessments:

1. Phenotypic censuses, which occurred at each harvesting event, consisted of sampling 25% of the population. These censuses fulfilled two functions: they provided a population census (population abundance and biomass) as well as a phenotypic assessment of population structure in terms of individual length, sex, and male maturity over time. In total, we performed 63 phenotypic censuses, which involved measuring on average 135 ± 52 individuals per population each time. In four of these, we also obtained data of female maturation and fecundity, which cannot be assessed without sacrificing the individual (Figure 1, Appendix S1: section 2.4).
2. Common‐garden assessments, which were conducted to measure life‐history traits under standardized conditions without directly confounding parental effects. These assessments thus allowed to determine whether phenotypic change in traits had a genetic basis and would persist across generations. They occurred roughly once a year during harvest phase 1, and once for recovery phase and harvest phase 2. In total there were six common‐garden assessments; the first one represents the populations prior any size‐dependent harvest, the following three represent harvest phase 1, while the last two represent the recovery phase and harvest phase 2, respectively (Figure 1). In each common‐garden assessment on average 15 ± 7 females and 17 ± 9 males per population were assessed. Males were assessed from birth until weeks after maturation, i.e., on average until they reached 137 ± 116 days old. Females were assessed from birth until after the birth of their second brood, i.e., on average until they reached 230 ± 100 days old. In the fourth common‐garden assessments, all individuals were assessed from birth to death. All assessed individuals were second‐generation offspring of four females randomly sampled per population and common‐garden assessment. All individuals (sampled females, first‐ and second‐generation offspring) were reared in isolation in 2 L tanks under common‐garden conditions: all tanks were in a flow‐through system at a constant temperature of 25°C (±0.5°C) and 12:12 light regime, with no harvest, and food equal for all individuals. The following life‐history traits were assayed: growth, age and size at maturation, fecundity, and lifespan. See Appendix S1: section 2.5 for details on feeding, maintenance, and measurements.

### 2.4 Population Demographics

In addition to demographic snapshots from each harvest (see section 2.3 above), we collected data on survival and age. During harvest phase 1, we VIE‐marked (Visible Implant Elastomer tags, Northwest Marine Technology Inc.; Appendix S1: section 2.4) and followed 28 cohorts, from which we estimated age (in 6‐week cycles), survival probability, and, together with the fecundity from the phenotypic census, population‐specific estimates of length‐dependent fecundity. These data were used to estimate generation time using the life table method (Molles Jr 2002, Appendix S1: section 3.3).

Fish that naturally perished were recorded throughout the experiment. The recorded deaths were mostly adult fish, as dead juveniles were rapidly ingested or degraded and hence were not commonly found. These deaths were caused by senescence or microparasites; no macroparasites were present in the system. Therefore, we refer to this mortality as *directly observed natural mortality*, calculated as the number of dead individuals during a 6‐week interval divided by the total abundance of the population at the beginning of the interval.

In addition, we estimated *total natural mortality* from mark‐recapture data obtained in harvest phase 1 (see above and Appendix S1: section 3.4). Within a cohort of marked individuals, the number *N*_*t*+1_ of individuals at time *t* + 1 equals the number *N*_*t*_ of individuals at time *t* times the probability of surviving harvest (*P*_hs_) and natural factors (*P*_ns_ = *e* ^−*μ*Δ*t*^), i.e., *N*_*t*+1_ = *e* ^−*μ*Δ*t*^ *P*_hs_ *N*_*t*_, where *μ* is total natural mortality expressed as an instantaneous rate. This mortality estimate includes directly observed natural mortality (as defined above) and any unobserved mortality.

### 2.5 Statistical Analysis

All statistical analyses were performed with software R 4.0.5 (R Core Team 2021), and all generalized mixed models (GMM) were performed with the R package glmmTMB (version 1.0.2.9000; Brooks et al. 2017). Model diagnostics were performed with R package DHARMa (version 0.4.5; Hartig 2022) to ensure that assumptions were met.

For the phenotypic and population censuses, we tested the fixed effects of harvest regime, phase in the experiment, and time (in weeks) within a phase, and their 3‐way interactions with GLMMs using population (9 levels) as random effect and controlling for the effect of length (Appendix S1: section 3). The dependent variables were the following traits, assuming Gaussian error distribution unless otherwise stated: (1) biomass, (2) length of males and females larger than 16 mm (SL), (3) probability of being mature for males and females (also referred to as maturity ogives; binomial distribution), with which we estimated the Length at which 50% of individuals are mature (L50), (4) fecundity (zero‐truncated negative binomial distribution), (5) natural mortality (directly observed: zero‐inflated gamma distribution, total: Poisson distribution), and (6) generation time. Data from all the 63 phenotypic censuses (Figure 1) were used, except for probability of being mature in females and fecundity, which were estimated with data from 4 phenotypic censuses (Figure 1). Hence, when modelling these two traits, time in phase was omitted. Generation time was estimated for harvest phase 1 only, with harvest regime as the only fixed effect. In addition, we estimated the rate of change in haldanes of the length at maturation in males and females in both harvest phases (assuming equal generation time; see Appendix S1: section 3).

For the common‐garden assessments, we tested the effects of harvest regime and phase in the experiment as fixed factors, and their interaction, with GLMMs using individual ID as random effect and controlling for the effect of length. We tested the following variables: (1) two parameters of a biphasic growth model (Quince et al. 2008; Boukal et al. 2014): the size‐independent coefficient of energy acquisition, *c*, and the relative reproductive investment *r*, (2) length at average age at maturation, (3) probability of becoming mature (i.e., the probabilistic maturation reaction norm; binomial distribution) with which we characterized as the length at which 50% of individuals became mature (also referred to as Lp50), (4) early fecundity as the number of offspring born in a female's first two broods, (5) lifetime fecundity (negative binomial distribution), (6) adult lifespan, as the time between maturation and natural death (details in Appendix S1: section 3). All traits were estimated for males and females, except the two fecundity traits that were only available for females. Lifetime fecundity and lifespan models contained Population as random factor instead of individual ID. The six common‐garden assessments were used to estimate all traits, except for lifetime fecundity and adult lifespan. The former was estimated using only the 4th and 6th assessments for which data beyond 330 days (100 days after average age of last brood) were available, and lifespan was estimated using only the 4th assessment when we observed individuals until their natural death. It should be noted that Lp50 is not directly comparable to L50 and length above 16 mm, as Lp50 represents the length at becoming mature and does not consider potential changes in length after maturation, as the other measures do.

To evaluate the demographic effects on life‐history traits, we performed elasticity analyses (Benton and Grant 1999). Elasticity here is defined as the proportional change of a trait (relative to its original value) for a proportional change in biomass. Elasticity estimates were calculated to describe the larger transitions from no size‐dependent harvest to size‐dependent harvest for the traits obtained from the phenotypic assessment. This resulted in the estimation of three elasticities per life‐history trait: (1) transition from the start of experiment to end of harvest phase 1, (2) from end of recovery to end of harvest phase 2, and (3) from the start of the experiment until the end of the experiment (end of harvest phase 2). The variables considered were the phenotypic assessments of length above 16 mm and L50 for males and females, as well as natural intrinsic mortality, because these were the only traits with enough data for all transitions.

## 3 Results

Here we present our results based on the three postulated effects of harvesting: whether harvesting (1) had demographic effects, as expected from all contributing processes (Baranov's fishing up effect, compensatory response, and fisheries‐induced evolution), (2) led to a phenotypic response, as expected when compensatory response and fisheries‐induced evolution take place, and (3) led to a genetic change, which would represent fisheries‐induced evolution. For simplicity here we present changes for four timepoints: before harvest started and at the end of harvest phase 1, recovery phase, and harvest phase 2. Results on detailed trends throughout the experiment can be found in Appendix S1 (section 4).

### 3.1 Harvesting Has Direct Demographic Effects That Depend on the Harvest Regime

#### 3.1.1 Biomass

**FIGURE 2:**
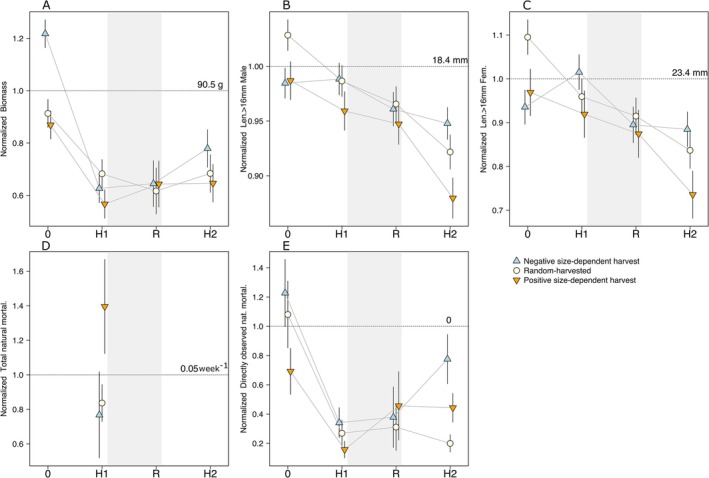
Changes in population demographics before harvest started (0 in *x*‐axis), and at the end of harvest phase 1 (H1), recovery phase (R), and harvest phase 2 (H2). Estimated values (symbols) and CI (whiskers) for (A) biomass, (B) mean length above 16 mm in males, (C) and females, (D) total natural mortality, and (E) directly observed natural mortality (dead individuals per 6 weeks and abundance) for Positive (orange inverted triangle), Random (beige circle), and Negative harvest (blue triangle). Each trait value is normalised by the mean of that trait before harvest started, represented by the dotted horizontal line with the actual value printed above it.

Over the course of the experiment there was an overall decrease in relative biomass in all populations, which was mainly driven by the biomass decrease between the start of the experiment and the end of harvest phase 1 (Figure 2A). This decrease in relative biomass was steepest for Negative harvest, partly due to its higher starting biomass, while it was most gradual for Random harvest (Table S3a). After that initial change, populations exposed to Positive and Random harvest did not experience significant changes in biomass among phases, while only populations exposed to Negatively size‐dependent harvest presented higher biomass at the end of harvest phase 2, relative to the end previous phases (Table S3a). Therefore, harvest regimes did not differ much in biomass at the different phases of the experiment. The only significant differences occurred at the end of harvest phase 1 between Positive and Random harvest (Random‐Positive, estimate (±SE) = 10.5 ± 3.5 g, *t*‐ratio = 2.96, df = 547, *p*‐value = 0.003), and at the end of harvest phase 2 between Positive and Negative harvest (Negative–Positive, estimate = 12 ± 4.7 g, *t*‐ratio = 2.56, df = 547, *p*‐value = 0.011). Those differences were much smaller relative to the initial harvest regime differences. Negative harvest had on average 30 g higher biomass than the other harvest regimes before size‐selective harvest started (Figure 2A; e.g., estimated difference between Positive and Negative harvest = 32 ± 3.5 g, *t*‐ratio = 9.04, df = 547, *p*‐value < 0.0001). Despite the initial difference, biomass equalised among harvest regimes during the Recovery phase and remained similar among harvest regimes (Figure 2A).

#### 3.1.2 Truncation of Size Structure

To address whether the size structure of the populations was truncated we focus on the average length of fish above the size limit, 16 mm. This average length presented an overall decrease for all harvest regimes, including the size‐independent harvest. Therefore, all populations presented a demographic truncation with fewer larger and probably older individuals. However, for both sexes, the decrease was fastest for populations exposed to Positive harvest and slowest for Negative harvest (Figure 2B,C). The observed decrease in average length above 16 mm in Negative harvest is mainly due to the sharp decrease occurring in the Recovery phase, where all populations experienced size‐independent harvest. The exposure to Negative harvest during harvest phase 1 led to an increase in average length in both males and females (Table S4a,b) and to no change in harvest phase 2 in males (Table S4a). Females exposed to Negative harvest presented a slight decrease in average length during harvest phase 2, but this was relatively smaller than the other changes described in the other phases (Table S4a).

Overall, populations exposed to Negative harvest had larger length than those exposed to Positive harvest by the end of both harvest phases, but these differences disappeared during the recovery phase (Figure 2B,C; Table S4a,b). For both males and females, differences were largest at the end of harvest phase 2. Females exposed to Positive harvest were on average (±SE) 3.5 ± 0.8 mm shorter than those exposed to Negative harvest (df = 22,484, *t*‐ratio = 4.36, *p*‐value < 0.0001), while males were 1.3 ± 0.2 mm shorter than those in Negative harvest (df = 18,016, *t*‐ratio = 5.69, *p*‐value < 0.0001). Fish exposed to Random harvest had an intermediate length, yet clearly different from the other two harvest regimes (Figure 2B,C; Table S4a,b). Overall, these changes in length were not elastic to changes in biomass (Table S4c). The only exception is in Positive harvest for the change between recovery and harvest 2 phases, where length was elastic and responded to the change in biomass in opposite directions (Table S4c). However, the biomass change in Positive harvest, between end of recovery and harvest phase 2, was not significant (Figure 2; Table S3a).

#### 3.1.3 Non‐Harvest Mortality: Total and Directly Observed Natural Mortality

Total natural mortality due to resource competition, cannibalism, and disease was higher in populations exposed to Positive harvest (*μ* = 0.0718 week^−1^) than in the other harvest regimes, which did not differ (*μ* = 0.0424 week^−1^; Figure 2D; Table S5a). This total natural mortality was not affected by population abundance (Table S5a).

Directly observed natural mortality presented a sharp decrease after the harvesting started (Figure 2E), being almost 2 times higher relative to the end of harvest phase 1 (Table S5b). After that, it was highest at the end of recovery phase, and lowest during the size‐dependent harvest phases (Figure 2E, Table S5b). Differences among size‐dependent harvest regimes were larger during the harvest phases relative to the recovery phase (Figure 2E). The largest differences occurred at the end of harvest phase 2, where all harvest regimes differed among each other, being directly observed natural mortality largest in Negative harvest and lowest in Random harvest. For instance, Positive harvest had 1.72 times lower mortality than Negative harvest (Table S5c). By the end of harvest phase 1, populations exposed to Positive harvest had 1.45 times lower mortality compared to Negative harvest (Figure 2E). Finally, by the end of the recovery phase, Positive and Negative harvest did not differ. However, Random harvest populations presented the lowest directly observed natural mortality—i.e., 1.75 times lower than Negative harvest (Table S5c). Directly observed natural mortality was elastic to biomass change for all transitions considered in all harvest regimes (Table S5d). A decrease in biomass, as between the start and the end of the experiment or throughout harvest phase 1, was linked to a decreased in directly observed natural mortality. However, between the recovery phase and harvest phase 2 this relationship was reversed for Random and Positive harvest, as biomass did not significantly change for those populations.

#### 3.1.4 Generation Time

Populations exposed to Positive harvest presented the shortest generation time (190 days), while those exposed to Negative harvest presented the longest ones. The average generation time (±SD) was 267 ± 67 days or about 1.4 generations per year. Positive harvest populations had on average (±SE) 2.7 ± 0.2 and 3.3 ± 0.2 months shorter generation time relative to Random harvest (*t*‐ratio = 13.00, *p*‐value < 0.0002) and Negative harvest, respectively (*t*‐ratio = 15.34; *p*‐value = 0.0001). Random and Negative harvest had similar generation times.

### 3.2 Size‐Dependent Harvest Affects Phenotypic Traits Differently Under Different Harvest Regimes

#### 3.2.1 Mean Size at Maturation: L50

**FIGURE 3:**
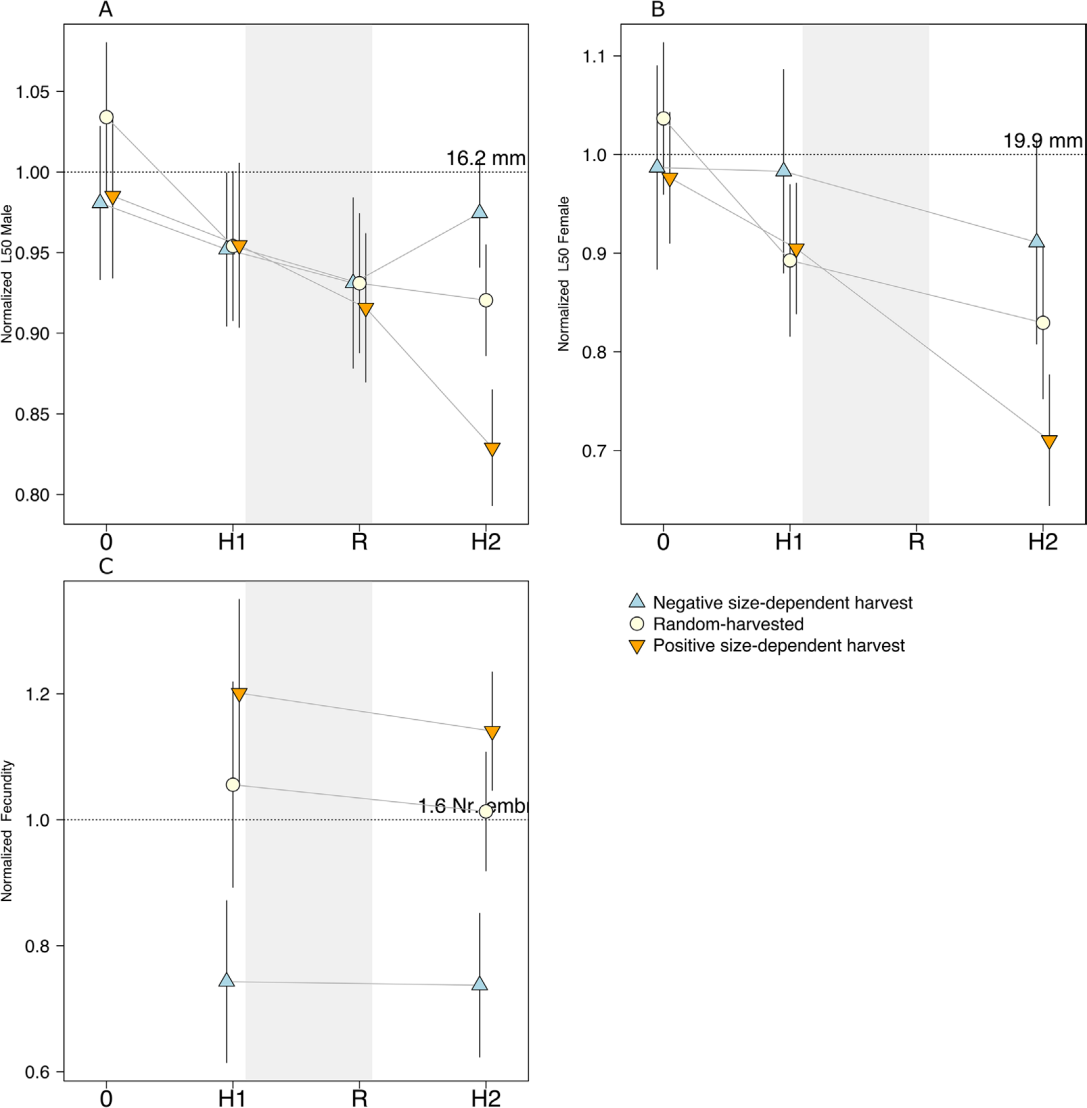
Phenotypic assessment before harvest started (0 in *x*‐axis), at the end of harvest phase 1 (H1), end of recovery phase (R), and end of harvest phase 2 (H2). Estimated values (symbols) and CI (whiskers) for the 3 traits assessed in males and females (panels) for Positive (orange inverted triangle), Random (beige circle), and Negative harvest (blue triangle). Each trait value is normalised by the mean of that trait before harvest started, which is represented by the dotted horizontal line and its actual value is printed above it. Nr. emb. refers to number of embryos.

Length at which 50% of males were mature (L50) changed over the course of the experiment (Figure 3A; Figure S3), and similar shifts were observed for females, despite having less data available on them (Figure 3B, Figure S3). Overall, L50 decreased throughout the experiment similarly as the average length of fish above 16 mm, but the descent was less steep. This overall trend was influenced by the different size‐dependent harvest regimes and was largely insensitive to biomass change for males (Table S6c). Populations exposed to Positive and Random harvest clearly presented an increase in the probability of maturing, and hence a decrease in L50, throughout the experiment (Tables S6 and S7a). For instance, males exposed to Positive harvest had 260 times higher odds of being mature at the end of harvest phase 2, relative to the beginning of the experiment (Table S6b), while for males exposed to Negative harvest these odds were only 3 times higher (Table S6b); these responses were insensitive to biomass change (Table S6c). However, in Negative harvest the descend in L50 was mainly driven by the probability of being mature increasing from the start of the experiment to the end of harvest phase 1, as there was no change between end of harvest phase 1 and recovery phase, and between the end of both harvest phases. There was an increase in probability of being mature between the end of recovery phase and harvest phase 2, but this was much lower than the initial value (Table S6b). All transitions in Negative and Random harvest were insensitive to biomass change, while in Positive harvest, male L50 was highly elastic to biomass between end of recovery and harvest phase 2, where an increase in biomass was linked to a decrease in L50 (Table S6c). However, the insignificant biomass change is unlikely to be the driver of the observed significant decrease in L50 and the odds of maturing 50 times larger by the end of harvest phase 2 relative to end of recovery phase (Figure S2; Table S6b). Female L50 was elastic relative to biomass change only between the start and the end of the experiment for Positive harvest (Table S6c). For Positive harvest a decrease in biomass of 20 g was linked with a decrease in L50 of 4 mm (Figure 3). However, a larger biomass decrease (49 g) in Negative harvest during the same transition (Table S3a) was not linked to a similar decrease in L50. Therefore, biomass might have influenced female L50 throughout the experiment but might not be the sole driver of change in L50, as it affected differently the harvest regimes.

Differences among size‐dependent harvest regimes in probability of being mature and hence in L50 were in the same direction as those observed in length above 16 mm, but the differences were somewhat smaller. We found no differences at the end of harvest phase 1 and recovery phase for either male or female (Figure 2A,B, Figure S3; Tables S6b and S7b). Yet, it should be noted that at the start of the recovery phase, which happened only 6 weeks after the end of harvest phase 1, we did find differences in probability of being mature in males (Table S6b). At the start of the recovery phase, males exposed to Positive or Random harvest had 3 times the odds of being mature relative to males exposed to Negative harvest (Table S6b). These initial differences at the start of the recovery phase are driving the differences observed in Figure 2A, as by the end of the recovery phase the harvest regimes no longer differed from each other (Table S6b). There are no data for females in the recovery phase and thus we cannot compare harvest regimes in that phase. Moreover, Positive and Random harvest presented a significant rate of decrease in male length at maturation of *h*_p(0.73)_ = −1.37 [−2.70, −0.04; *p*‐value = 0.044], and *h*_p(0.58)_ = −3.17 [−4.46, −1.87; *p*‐value < 0.001], respectively, while there was not a significant change for the Negative harvest *h*_p(0.56)_ = 0.33 [−1.81, 2.46; *p*‐value = 0.756] (Figure S4). Qualitative similar but weaker changes were observed for females, Random harvest: *h*_p(0.58)_ = −0.19 [−0.283, −0.101; *p*‐value = < 0.001], Positive harvest: *h*_p(0.73)_ = −0.03 [−0.053, −0.02; *p*‐value = 0.073], and Negative harvest: *h*_p(0.56)_ = 0.06 [−0.025, 0.138; *p*‐value = 0.058].

At the end of harvest phase 2 there were clear differences in the probability of being mature among harvest regimes in males and females (Figure 3A,B, Figure S3). Males and females exposed to Positive harvest were respectively 100 and 13 times more likely to be mature than those exposed to Negative harvest (Tables S6b and S7b). Therefore, fish exposed to Positive harvest presented shorter L50 relative to the other harvest regimes (Figure 3A,B). Fish exposed to Random harvest had intermediate L50, which was significantly different from the other harvest regimes. The differences among harvest regimes are larger by the end of harvest phase 2 than at the start of the recovery phase. In agreement with these results, length at maturation in Positive harvest males declined at a rate of h_p(0.71)_ = −2.64 haldanes [−3.90, −1.39; *p*‐value = 0.0003]. The rate of increase in length at maturity in Negative harvest males was faster than the rate of decrease in Positive harvest males (h_p(0.47)_ = 2.86 [0.44, 5.27; *p*‐value = 0.023]). The Random harvest lines did not show a significant phenotypic change in length at maturation throughout harvest phase 2 (h_p(0.53)_ = −0.28 [−2.29, 1.73; *p*‐value = 0.773]) (Figure S4).

#### 3.2.2 Fecundity

There was a general decrease in fecundity during the experiment (Figure 3C): females from harvest phase 1 had higher fecundity than in harvest phase 2. The trend is driven by females in harvest phase 2 having both a lower probability of having embryos and producing fewer embryos per litter (Table S8). We have no fecundity data for the start of the experiment or during the recovery phase.

Fecundity in females differed between Positive and Negative harvest regimes at the end of both harvest phases. A female of average length (19.6 mm) above the fecundity threshold (13 mm) exposed to Positive harvest would have on average 1.95 embryos and thus higher fecundity than a female exposed to Negative harvest, which would have 1.52 embryos (Figure 3C; Table S8).

### 3.3 Harvest‐Induced Phenotypic [and Demographic] Changes Are Partially Heritable and Thus Partially Genetic

#### 3.3.1 Length at Maturation in Common‐Garden Assessment

**FIGURE 4:**
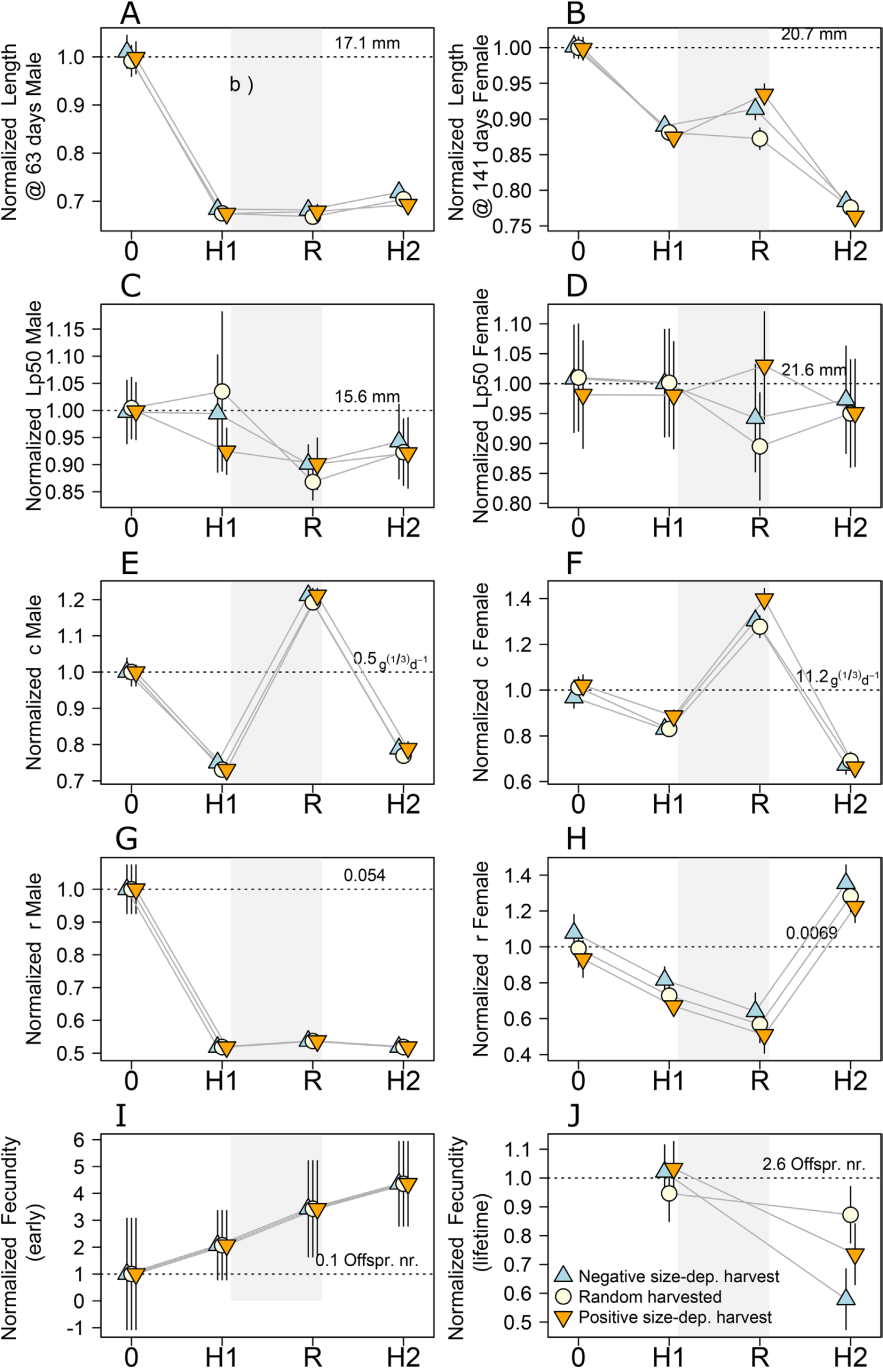
Common‐garden assessment over time (x‐axis) before harvest started (0), at the end of harvest phase 1 (H1), end of recovery phase (R), and end of harvest phase 2 (H2). Estimated values (symbols) and SE (whiskers) for the length‐, growth‐, and fecundity‐related traits assessed in males and females (panels) for Positive (orange inverted triangle), Random (beige circle), and Negative harvest (blue triangle). Each trait value is normalised by the mean of that trait before harvest started, which is represented by the dotted horizontal line and its actual value is printed above it.

Mean length of both males and females steeply declined over time in the experiment (Figure 4A,B). This decrease is much steeper in the common‐garden assessment (Figure 4A,B) relative to the phenotypic census (Figure 3A,B). In the case of the common‐garden assessment, it is mainly driven by the sharp decrease from the start of the experiment to the end of harvest phase 1. For males, the decrease is parallel for all harvest regimes (Figure 4A; Table S10a), while for females it is steeper for females exposed to Positive harvest (Figure 4B; Table S10b).

Length at age in males only differed among harvest regimes during harvest phase 2, where males exposed to Positive harvest were 0.44 mm shorter than males exposed to Negative harvest (Figure 4A; Table S10a). Females from the Positive harvest treatment were shorter than those from the Negative harvest treatment in both harvest phases (0.33 mm in phase 1; 0.44 mm, in phase 2; Figure 4B; Table S10b). These differences disappeared during the recovery phase; females from Positive harvest and negative harvest no longer differed, but both were larger than females exposed to Random harvest (e.g., 1.28 mm shorter than Positive harvest). In contrast, all differences among treatments in males at the end of Harvest phase 1 disappeared during the recovery phase.

#### 3.3.2 Probability of Becoming Mature From Common‐Garden Assessment

There was no consistent pattern of change in the length at which 50% of fish became mature (Lp50) among harvest phases (Figure 4C,D; Figures S6 and S7). It should be noted that Lp50, as estimated in the common‐garden assessment, is not directly comparable to L50 and length above 16 mm, as Lp50 represents the length at becoming mature and does not consider potential changes in length after maturation, as the other measures do. In males, there seems to be a decrease in Lp50 from the start of the experiment until the recovery phase, then an increase during harvest phase 2, although this trend was not parallel for all size‐dependent harvests (Figure 4C; Table S11a; Figure S6). In Positive harvest males, the initial decrease in Lp50 is driven by an increase in the odds of becoming mature from the start of the experiment to harvest phase 1, while in the other harvest regimes the descent in Lp50 occurred between harvest phase 1 and recovery phase (Table S11b). Moreover, the increase in Lp50 in harvest phase 2 occurred in the Random and Negative harvest treatments, but not in Positive harvest (Table S11b). In females, the general trends include a decrease in Lp50 during the recovery phase followed by an increase in harvest phase 2, being steeper for Positive harvest in both cases (Figure 4D; Table S12a,b; Figure S7).

Differences among harvest regimes varied with phase in the experiment. During harvest phase 1, males and females exposed to Positive harvest had twice and three times higher odds of maturing, respectively, and hence shorter Lp50 than Negative harvest (Figure 4C,D; Tables S11b and S12b). These differences in probability of maturing in the common‐garden assessment correspond to the differences observed in the phenotypic assessment for the probability of being mature at the start of the recovery phase. In recovery phase, the difference was maintained in females, while Random males had higher odds of becoming mature than Positive ones. All differences among harvest regimes disappeared in harvest phase 2, opposite to expectations (Tables S11b and S12b).

#### 3.3.3 Energy Acquisition and Allocation Between Growth and Reproduction in Common‐Garden Assessment

For both males and females, harvest phases 1 and 2 led to overall lower energy acquisition rate, *c*, relative to the initial and recovery phases (Figure 4E,F), implying a slower juvenile growth (Figures S8 and S9; Table S2a). In males, both harvest phases had equal and low *c*, while the highest *c* and thus the fastest juvenile growth occurred on the initial phase before size‐depended harvest started (Figure 4E Figure S8; Tables S13a,b and S2b). In females, the recovery phase had the highest *c*, while harvest phase 2 presented the slowest *c* and thus juvenile growth (Figure 4F and Figure S9; Tables S14a,b and S2c). Reproductive investment, *r*, in males and females declined throughout the experiment (Figure 4G,H). In males, this decline was concentrated in harvest phase 1 (Figure 4G; Table S13b). In females, there was an increase in *r* during harvest phase 2 (Figure 4H; Table S14b). These differences in *c* and *r* among phases (Figure 4E) resulted in different male growth curves throughout the experiment (Figure S8). In general terms, initial and recovery phases had steeper juvenile growth curves and an earlier shift towards the slower adult growth (due to larger *c* and *r*, respectively) than size‐dependent harvest phases.

Male growth curves differed very little among harvest regimes, as only fish exposed to Negative harvest presented a slower juvenile growth relative to fish exposed to random harvest (Figure S8), due to the lower energy acquisition rate, *c* (Table S13a). Juvenile growth of males did not differ between males exposed to Positive harvest and Negative harvest. There was no difference in reproductive investment, *r*, among males of different harvest regimes (Figure 4G). These differences together with the differences among phases, and those in length at maturation, resulted in overall males exposed to Positive harvest with growth curves that plateau at shorter lengths relative to the other harvest regimes in harvest phases 1 and 2. However, in the recovery phase, males exposed to Random harvest had growth curves that plateau at shorter length (Figure S8). Female growth curves were more strongly affected (through changes in α, *c* and *r*) by harvest regimes relative to males (Figure S9). Female juvenile growth was steeper—through higher values of coefficient in the energy acquisition rate, *c—*when exposed to Positive harvest relative to the other harvest regimes only during the harvest phase 1 and the recovery phase (Figure S9, Table S14b). Females exposed to Negative harvest presented a higher investment in reproduction, *r*, and hence an earlier shift towards adult growth relative to females exposed to Positive harvest in all phases (Figure S9; Table S14b).

#### 3.3.4 Fecundity: First Broods and Lifetime Fecundity in Common‐Garden Assessment

As with the phenotypic census, fecundity in the first broods of the common‐garden assessment was higher in harvest phase 2 relative to the initial phase and harvest phase 1. However, fecundity did not increase over time in the experiment (Figure 4I; Table S15a). Moreover, we did not observe differences among the harvest regimes in the common‐garden assessment (Table S15a), which contrasts with our results from the phenotypic census of fecundity (Table S1).

Lifetime fecundity decreased between harvest phase 1 and 2, but we do not have data on the other phases (Figure 4J; Table S15b). This decrease was largest for Negative harvest and non‐significant for Random harvest (Table S15b). This resulted in females exposed to Negative harvest presenting a lower lifetime fecundity during harvest phase 2 relative to the other harvest regimes. Lifetime fecundity did not differ among harvest regimes during harvest phase 1 (Figure 4J; Table S15b).

#### 3.3.5 Lifespan in Common‐Garden Assessment

Males exposed to Negative harvest had shorter mean (±SE) adult lifespans (409 ± 37 days) than males exposed to the other harvest regimes. Males from the Positive harvest treatment survived on average 586 ± 37 days. Females from the Positive harvest also had longer adult lifespans (644 ± 28.8 days), relative to other harvest regimes (Figure S10; Table S16). Harvest regime had no effect on reproductive lifespan. However, Positive harvest females had 95 days longer post‐reproductive lifespan than Random harvest (Figure S10; Table S16). All these differences in lifespan were presented at the end of harvest phase 1.

## 4 Discussion

Our decade‐long harvesting experiment allowing iteroparous life history showed that populations exposed to positively size‐dependent harvest, similar to that imposed by fisheries, had faster development and a higher natural total mortality rate in the population tanks, but increased individual lifespan when reared in isolation. Except for lifespan in isolation, these results broadly matched expectations according to life‐history theory and hence an evolutionary effect of size‐dependent mortality. However, some variables also presented common trends over the course of the experiment, which could be due to adaptation or habituation to the laboratory conditions or shared demographic effects. We compare our findings with earlier artificial selection experiments conducted on fish populations with semelparous life histories.

### 4.1 Size‐Dependent Harvest Affects Life‐History Traits Both Phenotypically and Genetically

The effect of size‐dependent harvest largely coincided with expectations. Populations exposed to Positive harvest showed (1) a phenotypic decrease in length at maturation during both size‐dependent harvest phases together with a decrease in L50 in harvest phase 2, also observed in the common‐garden assessment in harvest phase 1, (2) a phenotypic increase in fecundity without the accompanying genetic change in early life fecundity, but with a higher lifetime fecundity in the common‐garden assessment, (3) a heritable faster juvenile growth followed by a shorter adult length, and (4) higher natural mortality in the populations but longer lifespan in the common‐garden assessment, relative to populations exposed to Negative size‐dependent harvest.

We acknowledge that our common‐garden assessments do not conclusively prove that some changes in the populations were genetic: second‐generation common garden experiments do not rule out the possibility of transgenerational plasticity or heritable epigenetics. Nevertheless, such experiments are widely used to determine whether variability in a trait is heritable and thereby has a genetic basis (Conover and Munch 2002; Renneville et al. 2020; Wootton et al. 2021; Reznick and Travis 2019). In guppies, it has been shown that common‐garden evidence is consistent with quantitative genetic evidence (Travis et al. 2023). An interesting future avenue will be to perform quantitative genetic analyses to quantify the relative contribution of genetic and non‐genetic sources of variation in our studied traits.

Populations exposed to Positive harvest showed higher probability of being mature by the end of the experiment and shorter length at maturation, relative to fish exposed to Negative harvest. On average, there was a difference of 12% and 8% in length above 16 mm and L50 between the Positive harvest lines relative to Negative harvest and Random harvest, respectively. Similarly, in other selection experiments the difference between the positively size‐dependent and the random populations ranged 1%–22% (Conover and Munch 2002; Amaral and Johnston 2012; van Wijk et al. 2013; Uusi‐Heikkilä et al. 2015; Renneville et al. 2020; Bouffet‐Halle et al. 2021). Interestingly, in Renneville et al. (2020) and Bouffet‐Halle et al. (2021)'s experiments, the lines exposed to positively size‐dependent harvest did not evolve and the observed differences were driven by the lines exposed to Negative harvest becoming larger. In the present study, the changes were sex‐specific, for instance the difference between Positive and Negative harvest was of 17% in females and 7% in males for length above 16 mm. Similar results were expected for harvest phase 1, but these were not clearly found. Yet, earlier analyses, only considering harvest phase 1 showed a significant increase in the probability of being mature in fish exposed to Positive harvest (Diaz Pauli et al. 2014; Diaz Pauli et al. 2017). These differences among harvest regimes in the probability of being mature are observed in the present study at the start of the recovery phase, which represents just 6 weeks after the end of harvest phase 1, indicating that fish exposed to Positive harvest did have higher probability of being mature and thus shorter L50 after the first size‐dependent harvest phase. Moreover, the common‐garden assessment during harvest phase 1 showed that fish exposed to Positive harvest had twice the odds of becoming mature and this change had a genetic basis, which further justifies our interpretation that harvest regimes differed phenotypically at the end of harvest phase 1. The decrease in length at maturity (L50) through harvest phase 1 is also evident from the significant phenotypic rate of change in males exposed to Positive and Random harvest. Qualitatively equivalent results are found for females, but the change was smaller. Faster change in males relative to females in life history traits in guppies have also been found in the wild due to predation‐induced size‐dependent mortality (Reznick and Bryga 1987; Reznick et al. 1990; Reznick and Travis 2019). The phenotypic rate of change in length at maturity estimated here for Positive harvest (e.g., −1.37 to −2.64 haldanes) are larger than those estimated by van Wijk et al. (2013; 0.3 haldanes) and Uusi‐Heikkilä et al. (2015; −0.116 haldanes), despite our lower harvest intensity. Our haldanes estimates are also higher than those observed in wild guppies (0.02–0.15; Reznick et al. 1997; Hendry and Kinnison 1999; Reznick and Ghalambor 2005). Notice though that in wild guppies haldanes represented a genetic rate of change, while here we estimated phenotypic rate of change. Nevertheless, our haldanes are within the range of rates of contemporary evolution and phenotypic change estimated for 23 fish stocks exposed to size‐dependent harvest (−2.2 to 0.9 haldanes; Devine et al. 2012). The phenotypic differences among harvest regimes were much larger at the end of harvest phase 2 relative to harvest phase 1. However, those phenotypic changes were not paralleled by heritable changes in the common‐garden assessment during the harvest phase 2. We believe that this might be due to our last common‐garden assessment not being performed at the end of the harvest phase 2, but midway in that phase. Moreover, not all phases are represented equally by our common‐garden assessments. Our last common garden, representing harvest phase 2, started after 74 weeks of size‐dependent harvest, while our second, third, and fourth common‐garden assessments pooled together represented harvest phase 1 and were started 54, 112, and 158 weeks after size‐dependent harvest. Our last common‐garden assessment represented the shortest period of exposure to size‐dependent harvest, but also containing fish exposed to the recovery phase for 200 weeks (Figure 1). Thus, the assessment in harvest phase 2 might mostly represent a longer‐term adaptation to the recovery phase (when all populations experienced Random harvest), rather than already an adaptation to the second phase of size‐dependent harvest.

Life‐history changes towards faster life histories, due to Positive size‐dependent harvest, are expected to indirectly increase natural mortality, as these individuals require more resources to sustain the faster life history and become at higher risk of mortality by predators (Jørgensen and Holt 2013; Montiglio et al. 2018b). Increases in natural mortality have been observed in the wild and are likely synergic to fishing mortality magnifying its effects (Jørgensen and Holt 2013; Jacobsen and Essington 2018). Our results, indeed, showed that populations exposed to Positive size‐dependent harvest had higher total natural mortality (i.e., non‐harvesting‐related) compared to populations exposed to Negative size‐dependent harvest. Our total “natural” mortality is certainly lower than natural mortality in the wild, as our tank lacked non‐conspecific predators and macro‐parasites. Our experimental setting, despite simple, allowed for competition for resources, cannibalism, and some sporadic disease. Interestingly and contrary to expectations, fish exposed to Positive harvest presented lower directly observed mortality. Moreover, females exposed to Positive harvest presented higher immunocompetence relative to Negative harvest when artificially infected by a parasite common in the wild, but not present in our experimental populations (Bartusevičiūtė et al. 2022). Classical theories for the evolution of senescence predict early intrinsic mortality when exposed to high extrinsic mortality (predation, disease and harvesting), due to early investment in maturation and reproduction being paid as low investment in maintenance or reproduction later in life (Williams 1957; Reznick et al. 2004). Yet, we observed lower natural mortality both in the phenotypic census and common‐garden assessments in individuals exposed to Positive harvest, contrary to expectations. Similar results were observed in wild guppy populations exposed to high predation mortality. Guppies from natural populations with more diverse and abundant predators and correspondingly higher natural mortality rates (across all size‐classes; Reznick, Butler, et al. 1996) had longer adult and reproductive lifespans (Reznick et al. 2004), but equal post‐reproductive life to guppies from low predation environments (Reznick et al. 2006). Reznick et al. (2004) reasoned that the indirect decrease in density could explain such unexpected results, as older individual would benefit more of the extra available resources and then longer lifespans would be favoured. Overall our results match those of wild guppies where higher predator‐driven mortality rate, despite being size‐independent, led to earlier maturation, higher lifetime fecundity and longer lifespans (Reznick and Travis 2019). However, in wild guppies, it was the higher density in the populations without large predators that drove the life‐history change. In the present study, populations exposed to Positive harvest maintained relatively high abundance but low biomass (Figure S1; Table S3b). Therefore, fish in Positive harvest might have experienced higher resource competition per capita, but more abundant resources per body weight and hence weaker food limitation, relative to Negative harvest (as food ration was equal among populations). The absence of strong food limitation in Positive harvest is also indicated by faster cohort growth relative to other harvest regimes (Table S9; Figure S5). Despite large initial differences in biomass, biomass remained relatively constant over the rest of the experiment and similar among harvest regimes. Moreover, length at maturity (L50) responded similarly (and largely insensitive) to biomass in all harvest regimes. Consequently, size‐dependent harvest was the main driver of life‐history changes in the lab populations (but see next section) and the only cost observed is in terms of higher natural total mortality.

### 4.2 Demographic Effects and Habituation to Laboratory Conditions

Over the course of the experiment, there were some trends that were in the same direction in all treatments. Specifically, there was an overall decrease in biomass, L50, and in natural intrinsic mortality across all treatments. The decrease in biomass was mainly due to the initiation of harvest, and later the increasing harvest proportion and directly observed natural mortality during harvest phase 1. This initial decrease in biomass was strongest in Negatively size‐dependent harvest due to its higher initial biomass and abundance. After that initial decrease, biomass remained similar (and equal among populations) during the recovery phase but increased during harvest phase 2, although it never returned to the initial high levels. By the end of the experiment, Negative harvest had a slightly larger biomass than Positive harvest, but neither differed from Random harvest. Overall, our harvest and equal food supply tended to equalize the biomass among populations and hence limited different demographic effects. The lower harvest proportion at the start of the experiment and in the recovery phase, and hence higher biomass, probably explained the higher directly observed natural mortality in these time points, even though the experimental harvest did not target individuals in lower health, as natural predators would do (Lopez and Duffy 2021).

There was also a reduction in average length above 16 mm and L50 between the start and the end of the experiment, but it was more pronounced for Positive and Random harvest than for Negative harvest. This reduction in length was mainly driven by an initial biomass decrease from the start of the experiment to the end of harvest phase 1, which was strongest in Negative harvest rather than in Positive and Random harvests. The biomass decrease during this time could have resulted in higher food availability, which could explain a higher maturation probability and thus a lower L50. However, the common decrease in L50 does not seem to be driven solely by a decrease in biomass or abundance, as such a decrease was strongest in Negative harvest, and the decrease in L50 was strongest in Positive harvest. Moreover, by the end of harvest phase 2, L50 in Positive harvest was even shorter, yet there was no change in biomass. Even though low biomass could partly explain the short L50 in Positive harvest by the end of harvest phase 1, this demographic response cannot explain the short L50 by the end of harvest phase 2. Therefore, the reduction in biomass could not be the sole driver of the changes in L50 and the effect of size‐dependent harvest could be detected above the effect of biomass.

The reduction in length above 16 mm between the start of the experiment and harvest phase 1 had a genetic basis, as it was also observed in the common‐garden assessment. However, there was not a decrease in Lp50 between the start and the end of the experiment common for all harvest regimes, as Lp50 changed differently among harvest regimes. We, therefore, suggest that these common trends in length represent a combination of plastic and demographic responses in the tanks after the harvest commenced, and or habituation to the laboratory conditions. But this was not directly driven by biomass alone and was experienced differently among the harvest regimes. Another trait that presented adaptation to laboratory conditions in our populations was body morphology, i.e., a change towards more streamlined shape (narrow caudal peduncle) probably due to steady swimming in the large laboratory tanks and absence of predators (Langerhans and Reznick 2010; Idris 2016). Despite the initial parallel change in body shape in all harvest regimes, by harvest phase 2 Positive harvest fish presented a more streamlined shape relative to fish exposed to Negative harvest, which presented deeper bodies and shorter caudal fins (Hallingstad 2023). Therefore, adaptation or habituation to laboratory conditions might have been driven by a combination of increased mortality, which initially was only weakly size‐dependent, reduction of biomass, open water habitat and/or absence of predators. But the effect of the different size‐dependent harvest in the expected directions was still visible over these other laboratory effects. Unintentional selection parallel to all lines was also reported in earlier studies, either by habituation to captivity or by unforeseen selection for high fecundity due to the experimental design (Conover et al. 2009; Salinas et al. 2012; Uusi‐Heikkilä et al. 2015; Renneville et al. 2020), which led to genetic changes (Uusi‐Heikkilä et al. 2017). Therefore, future experiments should take this into consideration.

### 4.3 Natural Versus Artificial Selection Experiments

The term “artificial selection experiment” refers to when the experimenter is the agent of selection by controlling which individual gets to breed each generation, while keeping the environmental conditions common among treatments. “Natural selection experiment” refers to when the experimenter only controls one environmental factor, whereas the selective pressures and who gets to breed are driven by the environment (see glossary; Garland 2003; Diaz Pauli and Heino 2014). Notice that according to this definition, also laboratory experiments, such as ours, can qualify as natural selection experiments.

Until recently, all selection experiments designed to study fisheries‐induced evolution have followed artificial selection experiment designs. The experiments entailed semelparous species (Conover and Munch 2002) or discrete‐generations experimental designs that forced iteroparous species to exhibit semelparity (Amaral and Johnston 2012; van Wijk et al. 2013; Uusi‐Heikkilä et al. 2015; Renneville et al. 2020; Wootton et al. 2021). Therefore, the trade‐offs between current and future investments were inhibited from unfolding naturally under dynamic demographic environments (Diaz Pauli and Heino 2014). The present study, together with the one by Bouffet‐Halle et al. (2021) that also allowed for iteroparous life history and the interplay between demographic and size‐dependent effects of harvesting, are exceptions to this tradition. However, Bouffet‐Halle et al.*'s* (2021) experiment was only minimally iteroparous: there were two age classes, which both could reproduce in the control treatment, whereas in the positively size‐dependent harvest treatment all 1+ fish were removed, thereby reducing the life history into semelparity (Bouffet‐Halle et al. 2021).

Natural selection experiment, such as Bouffet‐Halle et al.*'s* (2021) and the present study, allows us to assess the effect of size‐dependent harvest relative to the demographic changes also induced by harvesting. Interestingly, the difference between harvested and non‐harvested populations in Bouffet‐Halle et al.*'s* (2021) experiment was driven by density dependence favouring a larger body size in unharvested populations, rather than positively size‐dependent harvest favouring a smaller size. We also saw this density‐dependent effect on size, as a decrease in length above 16 mm in Negative harvest populations at the end of harvest phase 2, when biomass increased. Moreover, natural and artificial selection experiments may reach dissimilar conclusions about harvest yield, as pointed out elsewhere (Diaz Pauli and Heino 2014; Heino et al. 2015). Natural selection experiments showed that positively size‐dependent harvest led to higher biomass yield (Edley and Law 1988; Diaz Pauli and Heino 2014; Heino et al. 2015), as also observed in the present study during both harvest phases (Figure S2). In contrast, artificial selection experiments have concluded that positively size‐dependent harvest leads to lower biomass yield (Conover and Munch 2002; Heino et al. 2015).

In addition, natural selection experiments allow trade‐offs between current and future reproduction to unfold, contrary to artificial selection experiments that exhibit semelparity and thus have no future reproduction. When length or length at maturation is assessed, the two types of experiment show similar outcomes: most studies showed a decrease in length in the fish exposed to positively size‐dependent harvest (see section above). However, this might not be the case for other traits, such as juvenile growth and fecundity. For instance, the studies that assessed fecundity concluded that it was reduced in positively size‐dependent harvested populations (Walsh et al. 2006; Amaral and Johnston 2012), showed no difference relative to Negative harvest (Renneville et al. 2020), or presented mixed results Uusi‐Heikkilä et al. (2015) reported lower, while Sbragaglia et al. (2021) reported higher fecundity for the same lines. In the present study we saw an increased fecundity both in the phenotypic census and in the common‐garden assessment. Our results on fecundity partially agree with the expectation that Positive size‐dependent harvest resulting in early maturation should also entail higher fecundity (Charlesworth 1994; Reznick, Reznick et al. 1997; Heino et al. 2015; Montiglio et al. 2018b). But we also found no difference in the common‐garden assessment of early life fecundity and lower investment in reproduction, *r*, in Positive relative to Negative harvest, as also shown in a related study only considering harvest phase 1 (Diaz et al. 2017). Reproductive investment might not only be investment in gonads, but also in secondary sexual traits and sexual behaviours, which might explain the contradictory results. Therefore overall, our fecundity results partially agree with the increase fecundity expectation. In the other natural selection experiment, positively size‐dependent harvest populations had lower fecundity than non‐harvested populations (Bouffet‐Halle et al. 2021). However, Bouffet‐Halle et al. (2021)'s harvested populations (but not the controls) were forced to semelparity and this experimental set‐up difference might be the reason their fecundity results oppose ours. Therefore, the experiment by Bouffet‐Halle and colleagues might be closer to the artificial selection experiments than natural selection experiments.

Another important difference between natural and artificial selection experiments is whether Random harvest can be interpreted as a control treatment. In artificial selection experiments with semelparous life histories, terminal Random harvest is evolutionarily unselective and thus can act as a control, as far as it is properly executed (Heino et al. 2015). However, in natural selection experiments with iteroparity (such as ours), individuals are exposed to repeated Random harvest, which changes life expectancy of mature individuals and thus the value of their future reproduction. If the effect of Random harvest is to increase overall mortality among adults, it is expected to favour evolution of a faster life history, albeit at a slower pace than in a population exposed to Positive size‐dependent harvest (Stearns 1992; Heino et al. 2015).

Here, we presented at times clear differences among the three harvest regimes (e.g., L50 in harvest phase 2), where the individuals exposed to Random harvest presented intermediate values, while those exposed to Negative and Positive harvest present upper and lower extreme values. Still, populations exposed to Random harvest presented changes throughout the experiment. Between end of recovery and end of harvest phase 2, both Random and Positively size‐dependent harvest drove an increase in probability of being mature and decrease in L50, but the latter was steeper. But during that transition, biomass did not change in either Positive or Random harvest populations. Moreover, in the present study a lack of difference between Positive and Random harvested populations might not indicate lack of change, as in earlier selection experiments that forced semelparous life‐histories (e.g., Uusi‐Heikkilä et al. 2015; Renneville et al. 2020), but that the change in both harvest regimes was similar (e.g., female length above 16 mm at end harvest phase 1), probably due to habituation to laboratory conditions (Appendix S1: section 4.4).

It should be noted however, that experiments frequently exhibit unforeseen selective forces, even in the seemingly unselective treatments of artificial selection experiments. For instance, there was an unintended fecundity selection in Uusi‐Heikkilä et al. (2015) and Renneville et al. (2020) and selection linked with social interactions in Uusi‐Heikkilä et al. (2015). Moreover, forcing a naturally iteroparous species into a semelparous life history may hinder the trade‐off between early and late investments, leading to somewhat artificial results (Uusi‐Heikkilä et al. 2015). Therefore, differences between the present study and earlier selection experiments are not unexpected, given the large differences in experimental settings.

### 4.4 Evolutionary Recovery

The life‐history changes that occurred during harvest phase 1, e.g., shorter length above 16 mm in males and females, and shorter L50 in males exposed to Positive harvest relative to fish exposed to Negative harvest, were present in both the phenotypic and common‐garden assessments indicating a certain genetic basis to the change. The changes in L50 and Lp50 were maintained through the 154 weeks of the recovery phase (i.e., Random harvest in all populations), but not after the almost 1 year of break in harvest. Thus, the changes in size and size at maturation could be reversed, as observed elsewhere (Conover et al. 2009). The shorter generation time probably aided the recovery of Positive harvest populations. However, shorter length above 16 mm did not recover and was maintained in the common‐garden assessment from harvest phase 2. The lack of recovery in length and growth‐related genes also occurred in Uusi‐Heikkilä et al. (2015)'s experiment after 13 generations without harvest (Uusi‐Heikkilä et al. 2017; Roy et al. 2021), but there is no data on whether maturation or fecundity recovered. Therefore, different traits have different recovery rates (Salinas et al. 2012) and rates of change are sex‐specific, as also observed in the wild (Reznick et al. 1997). In addition, the different recovery rates depend on the unintentional selection that captive breeding may impose in the different experiments.

### 4.5 Concluding Remarks

Here we have presented results of a decade‐long experiment where iteroparous life histories, density‐dependent processes, semi‐natural selection, and trade‐offs among life‐history traits have been allowed. Positively size‐dependent harvest, i.e., when large individuals are targeted by the harvest, as is common in fisheries, presented evolution towards smaller size and faster life histories. Positive harvest also led to faster juvenile growth, but slower adult growth; these changes were most probably adaptive, rather than due to demographic processes, although some habituation the lab conditions initially occurred. In addition, Positive harvest resulted in maturation at smaller size and in higher lifetime fecundity, but unexpectedly, in longer lifespan. Thus, our experiment shows that harvested populations do adapt to size‐dependent mortality. Our results on length and maturation agree with earlier artificial selection experiments, but differences in the modelled life histories among experimental designs also led to different results in fecundity and juvenile growth. Most of the changes, but not all, were reversible after the recovery phase (the most notable exception being length). Populations exposed to Positive harvest presented higher natural total mortality, despite the lower intrinsic mortality, as the only obvious cost of adaptation. Future research should disentangle how this cost of adaptation to faster life history is paid, whether there is a lower competitive ability, lower metabolic scope or other trade‐off, that is driving the higher mortality rate. Moreover, the demographic and ecosystem consequences of these life‐history changes should be investigated to better understand the impact of size‐dependent mortality, and harvest pressure, on fish populations.

## Funding

This work was supported by Norges Forskningsråd, 214189, 275125.

## Ethics Statement

The experiment was performed at the fish facilities at the University of Bergen. The experiment followed the Norwegian and University of Bergen animal welfare regulations, and it was approved by the Norwegian Food Safety Authority (Decision regarding the use of animals in procedures—FOTS IDs: 1639, 2804, 4337, 5562, 6501, and 21,812).

## Conflicts of Interest

The authors declare no conflicts of interest.

## Supporting information


**Table S1:** Description of the populations in October 2010 when the harvest started. Sex and length come from a sample of 25% of individuals in the population. Mean length and standard deviation (SD) in milimeters, abundance is in number of individuals and biomass in grams.
**Table S2:** (a) Values of biphasic growth model parameters fixed in the final estimation of coefficient in the energy acquisition rate, *c*, and investment in reproduction, *r*. (b) Results from natural‐logged ([log]) weight‐length relationship model for males. Length was standardised to 16 mm (Length std). (c) Results from natural‐logged ([log]) weight‐length relationship model for females. Length was standardised to 16 mm (Length std).
**Figure S1:** Average (a) abundance and (b) biomass per harvest regime throughout the size‐selection experiment. Different harvest phases are shown with white areas, while the recovery phase is marked with a dark grey area, and the break phase with pale grey area. The three harvest regimes are Negative size‐dependent harvest (blue triangles), Random harvest (white circles), and Positive size‐dependent harvest (orange inverted triangles).
**Table S3:** (a) Results from the biomass model with Gaussian distribution. Estimates and confidence intervals (CI) in grams. (b) Results from the abundance model with Gaussian distribution. Estimates and confidence intervals (CI) in grams.
**Figure S2:** Total biomass yield and mean individual weight throughout the size‐selection experiment: at the start of the experiment (1st panel) and at the end of harvest phase 1 (2nd panel), recovery phase (3rd panel), and harvest phase 2 (4th panel) for the three harvest regimes, Negative size‐dependent harvest (blue triangles), Random harvest (white circles), and Positive size‐dependent harvest (orange inverted triangles).
**Table S4:** (a) Results from length above 16 mm model with Gaussian distribution in males. Estimates and confidence intervals (CI) in mm. (b) Results from length above 16 mm model with Gaussian distribution in females. Estimates and confidence intervals (CI) in mm. (c) Elasticity estimates for a change in average length above 16 mm in males and females relative to a change in biomass for three transitions: (1) from start of experiment (start) to end of harvest phase 1 (H1), (2) from end of recovery phase to end of harvest phase 2 (H2), and (3) from start of experiment to end of experiment (H2), for the three harvest regimes.
**Table S5:** (a) Results from total natural mortality model with Poisson distribution where total natural mortality *μ* is—Log‐estimate/6 weeks interval in week^−1^. The value from the intercept refers to the estimate in the Random harvest regime, while in Negative and Positive harvest indicate the difference in mortality rate with Random harvest. (b) Results from directly observed natural mortality model with gamma and zero inflated distribution. Note the zero‐inflated model shows probability of having zero mortality. (c) Harvest regime pairwise comparison of directly observed natural mortality. Pairwise comparison between beginning and end of harvest phase 1. Degrees of freedom = 1656. (d) Elasticity estimates for a change in directly observed natural mortality relative to a change in biomass for three transitions: (1) from start of experiment (start) to end of harvest phase 1 (H1), (2) from end of recovery phase to end of harvest phase 2 (H2), and (3) from start of experiment to end of experiment (H2), for the three harvest regimes.
**Figure S3:** Length at which the probability of being mature is 50% (L50), 25% (L25) and 75% (L75) for males (L50 = symbols, L25 = dashed lines, L75 = dotted lines) and females (L50 = symbols, L25 and L75 = whiskers; jittered to avoid overlap), for each experimental phase: harvest phase 1 is in the left panel, recovery phase in the central shaded panel, and harvest phase2 in the right panel; and harvest regime: Positive (orange inverted triangles), Random (circles), and Negative harvest (blue triangles).
**Figure S4:** Trends for mean length in mature males over time in harvest phase 1 (left panel), recovery phase (central panel), and harvest phase 2 (right panel). Change rate in haldanes within harvest phase 1 and harvest phase 2 shown in legends. Symbols refers to Positive (orange inverted triangles and dashed lines), Random (yellow circles and solid lines), and Negative harvest (blue triangles and dotted lines).
**Table S6:** (a) Results from the probability of being mature model with binomial distribution in males. (b) Harvest regime pairwise comparison of probability of being mature in males at the start and the end of each experimental phase: harvest phase 1, recovery phase and harvest phase 2. Degrees of freedom = 43,013. (c) Elasticity estimates for a change in L50 in males and females relative to a change in biomass for three transitions: (1) from start of experiment (start) to end of harvest phase 1 (H1), (2) from end of recovery phase to end of harvest phase 2 (H2), and (3) from start of experiment to end of experiment (H2), for the three harvest regimes.
**Table S7:** (a) Results from the probability of being mature model with binomial distribution in females. (b) Harvest regime pairwise comparison of probability of being mature in females at the 4 events assessed, three during harvest phase 1, and 1 during harvest phase 2. Degrees of freedom = 2583.
**Table S8:** Results from fecundity (embryo count) model with negative binomial and zero truncated distribution. Length is standardised by 16 mm. to Note the zero‐truncated model shows probability of having zero embryos.
**Table S9:** Results from the cohort length model with Gaussian distribution. Estimates and confidence intervals (CI) in mm. Age and biomass are standardized to zero mean and unity standard deviation. Mean and SD for age 4.3 ± 3.2 6‐weeks cycle (i.e., 27 weeks old), and biomass 78.6 ± 22.1 g.
**Figure S5:** Cohort growth curves form mark‐recapture fish in phenotypic assessment during harvest phase 1 for females (top panels) and males (bottom panels) and under high biomass (left panels) and low biomass (right panels).
**Table S10:** (a) Harvest regime pairwise comparison of length at 63 days old (mean age at maturation) in males at the 6 genotypic assessments. Degrees of freedom = 5417. (b) Harvest regime pairwise comparison of length at 141 days old (mean age at maturation) in females at the 6 genotypic assessments. Degrees of freedom =11,426.
**Figure S6:** Length and age at which the probability of becoming mature is 50% (Lp50), 25% (Lp25) and 75% (Lp75) for males (Lp50 = symbols, Lp25 = dashed lines, Lp75 = dotted lines), for each experimental phase: initial phase (before size‐selection) in the first pale‐shaded panel, harvest phase 1, recovery phase dark‐shaded panel, and harvest phase 2 in the right panel; and harvest regime: Positive (orange inverted triangles), Random (yellow circles), and Negative harvest (blue triangles).
**Table S11:** (a) Results from the probability of becoming mature model with binomial distribution in males. (b) Harvest regime pairwise comparison of probability of becoming mature in males (genetic assessment from 6 common garden experiments) during four phases: initial phase, harvest phase 1, recover, and harvest phase 2 for males of average age and length at maturation. Degrees of freedom = 4134.
**Table S12:** (a) Results from the probability of becoming mature model with binomial distribution in females. (b) Harvest regime pairwise comparison of probability of becoming mature in females (genetic assessment from 6 common garden experiments) during four phases: initial phase, harvest phase 1, recover, and harvest phase 2 for females of average age and length at maturation (comparison for all harvest phases). Degrees of freedom = 7717.
**Figure S7:** Length and age at which the probability of becoming mature is 50% (Lp50), 25% (Lp25) and 75% (Lp75) for females (Lp50 = symbols, Lp25 = dashed lines, Lp75 = dotted lines), for each experimental phase: initial phase (before size‐selection) in the first pale‐shaded panel, harvest phase 1, recovery phase dark‐shaded panel, and harvest phase 2 in the right panel; and harvest regime: Positive (orange inverted triangles), Random (yellow circles), and Negative harvest (blue triangles).
**Figure S8:** Male biphasic growth curves for males in the different phases of the experiment (panels) for Negative (dashed black line), Random (solid black line), and Positive (dotted black line) size‐selective harvest. Individual fish growth curves are shown in grey.
**Table S13:** (a) Male biphasic growth models estimation of coefficient in the energy acquisition rate, *c*, and reproductive investment, *r*, given the fixed parameters in Table S2a. SE refers to standard error and CI to confidence intervals. Results from harvest regime and phase comparisons are in Table S13b. (b) Comparison of male biphasic growth models parameters: coefficient in the energy acquisition rate, *c*, and reproductive investment, *r*, among phases in the experiment and Harvest regimes. SE refers to standard error. Parameters fixed in the biphasic model are given in Table S2a, and estimated values of *c* and *r* are given in Table S13a.
**Figure S9:** Female biphasic growth curves for males in the different phases of the experiment (panels) for Negative (dashed black line), Random (solid black line), and Positive (dotted black line) size‐selective harvest. Individual fish growth curves are shown in grey.
**Table S14:** (a) Female biphasic growth models estimation of coefficient in the energy acquisition rate, *c*, and reproductive investment, *r*, given the fixed parameters in Table S2a. SE refers to standard error and CI to confidence intervals. Results from harvest regime and phase comparisons are in Table S14b. (b) Comparison of female biphasic growth models parameters: coefficient in the energy acquisition rate, *c*, and reproductive investment, *r*, among phases in the experiment and Harvest regimes. SE refers to standard error. Parameters fixed in the biphasic model are given in Table S2a, and estimated values of *c* and *r* are given in Table S14a.
**Table S15:** Results from fecundity (a) offspring count of first two broods with Poisson distribution, where length is standardised by 16 mm, and (b) lifetime offspring count with negative binomial distribution.
**Figure S10:** (a) Adult, (b) Reproductive, and (c) Post‐reproductive lifespans for males (thicker symbol stroke, only in (a)) and females exposed to different harvest regimes: Negative (blue triangle), Random (circle), and Positive (orange inverted triangle) harvests.
**Table S16:** Pairwise comparison between harvest regimes for adult lifespan in males and females, and reproductive and post‐reproductive lifespans for females only. Estimates are in days. Degrees of freedom are 251 for adult lifespan, and 92 for reproductive and post‐reproductive lifespans.

## Data Availability

Data is available in Zenodo DOI https://doi.org/10.5281/zenodo.20171625.
