# Supplementary material for: Fast Phenotypic and Genetic Changes in Life History and Mortality of Experimental Populations of Guppies (Poecilia reticulata) Exposed to Size‐Dependent Harvest in a Decade‐Long Selection Experiment
Source: Evol Appl. 2026 May 18;19(5):e70253. doi: 10.1111/eva.70253 (PMC13183784; doi:10.1111/eva.70253)
Supplement: Supplementary file 1 — Table S1: Description of the populations in October 2010 when the harvest started. Sex and length come from a sample of 25% of individuals in the population. Mean length and standard deviation (SD) in milimeters, abundance is in number of individuals and biomass in grams. Table S2: (a) Values of biphasic growth model parameters fixed in the final estimation of coefficient in the energy acquisition rate, c, and investment in reproduction, r. (b) Results from natural‐logged ([log]) weight‐length relationship model for males. Length was standardised to 16 mm (Length std). (c) Results from natural‐logged ([log]) weight‐length relationship model for females. Length was standardised to 16 mm (Length std). Figure S1: Average (a) abundance and (b) biomass per harvest regime throughout the size‐selection experiment. Different harvest phases are shown with white areas, while the recovery phase is marked with a dark grey area, and the break phase with pale grey area. The three harvest regimes are Negative size‐dependent harvest (blue triangles), Random harvest (white circles), and Positive size‐dependent harvest (orange inverted triangles). Table S3: (a) Results from the biomass model with Gaussian distribution. Estimates and confidence intervals (CI) in grams. (b) Results from the abundance model with Gaussian distribution. Estimates and confidence intervals (CI) in grams. Figure S2: Total biomass yield and mean individual weight throughout the size‐selection experiment: at the start of the experiment (1st panel) and at the end of harvest phase 1 (2nd panel), recovery phase (3rd panel), and harvest phase 2 (4th panel) for the three harvest regimes, Negative size‐dependent harvest (blue triangles), Random harvest (white circles), and Positive size‐dependent harvest (orange inverted triangles). Table S4: (a) Results from length above 16 mm model with Gaussian distribution in males. Estimates and confidence intervals (CI) in mm. (b) Results from length above 16 mm model with Gau [file EVA-19-e70253-s001.docx]

**Supplementary material**

Fast phenotypic and genetic changes in life history and mortality of experimental populations of guppies (*Poecilia reticulata*) exposed to size-dependent harvest in a decade-long selection experiment.

[1. Important differences with earlier selection experiments 1](#_Toc221818266)

[1.1 Harvest rate 1](#_Toc221818267)

[1.2. Target of selection 2](#_Toc221818268)

[1.3 Criticisms to earlier studies 2](#_Toc221818269)

[2. Detailed material and methods 2](#_Toc221818270)

[2.1 Founder populations 2](#_Toc221818271)

[2.3 Harvesting protocol 4](#_Toc221818272)

[2.4 Phenotypic census: 4](#_Toc221818273)

[2.5 Common-garden assessment 5](#_Toc221818274)

[3. Statistical analyses 6](#_Toc221818275)

[3.1 List of variables 6](#_Toc221818276)

[3.2 Population demographics I: Biomass and abundance 7](#_Toc221818277)

[3.3 Population demographics II: Generation time and phenotypic rate of change 7](#_Toc221818278)

[3.5. Phenotypic census of life history I: L50 and probability of being mature 8](#_Toc221818279)

[3.7. Phenotypic census of life history III: Cohort somatic growth 9](#_Toc221818280)

[3.8. Common-garden assessment of life history I: Lp50 and probability of becoming mature 9](#_Toc221818281)

[3.9. Common-garden assessment of life history II: Individual growth, energy acquisition rate, c, and investment in reproduction, r 10](#_Toc221818282)

[3.10. Common-garden assessment of life history III: First-broods fecundity and lifetime fecundity 12](#_Toc221818283)

[3.11. Common-garden of life history IV: lifespan 12](#_Toc221818284)

[4. Results on detailed trends throughout the experiment 12](#_Toc221818285)

[4.1. Harvesting has direct demographic effects that depend on harvest regime 13](#_Toc221818286)

[4.2. Size-dependent harvest affects phenotypic traits differently under different harvest regimes 19](#_Toc221818287)

[4.3. Harvest-induced phenotypic [and demographic] changes are partially heritable and thus partially genetic 29](#_Toc221818288)

[5. References 42](#_Toc221818289)

# Important differences with earlier selection experiments

## 1.1 Harvest rate

The average harvest rate in the present experiment was 40%, with a maximum intensity of 60% and a minimum of 25%. This proportion would correspond to harvest proportion *P*/2 at the population level, assuming equal numbers of small and large fish. The harvest rate was modified depending on the average abundance per population. A steady increase in abundance indicated that the populations could sustain higher harvest rates and thus it was increased (Figure 1). Our aim was to avoid abundances larger than 800 fish and hence overcrowding, although this was not possible in three harvest, as we did not want to increase harvest rate above 60% due to earlier criticisms (Brown *et al.* 2008; Hilborn & Minte-Vera 2008). These harvest rates are within or below what wild populations are likely to experience (Reznick *et al.* 1996a), and are also in the range seen in commercial fisheries if we liken our 6-week harvest cycles to annual cycles in the wild. For example, natural and fishing mortality of Atlantic cod, *Gadus morhua*, from the coast of Skagerrak, Norway, were estimated to be 25% and 50% in one year, respectively (Olsen & Moland 2011). All earlier selection experiments in this field used more intensive fishing rates (82 ± 6 % mean ± SD from 6 experiments), except Amaral and Johnston (2012) who had an average fishing rate of 33% per harvest event, and a maximum of 54%.

## 1.2. Target of selection

Unlike earlier selection experiments our harvest events did not occur at a fixed age, (as in Conover and Munch, 2002; Amaral and Johnston, 2012; Renneville *et al.*, 2020b) or only on mature individuals (Amaral & Johnston 2012; Conover & Munch 2002; Renneville *et al.* 2020; van Wijk *et al.* 2013).

## 1.3 Criticisms to earlier studies

The main criticisms posed to the first selection experiment to study fisheries-induced evolution (Conover & Munch 2002) can be summarized as 1) the lack of direct genetic evidence for the observed phenotypic changes (Kuparinen & Merilä 2008), 2) exaggerated fishing selectivity compared to real fishing pressure (Brown *et al.* 2008), 3) unrealistic experimental conditions that do not resemble natural populations (Hilborn 2006), and 4) lack of considering the role of environmental drivers (Andersen & Brander 2009; Browman *et al.* 2008). The unrealistic experimental conditions refer to populations with discrete generations, absence of density feedback, lack of potential for natural selection, and use of model species different from typical commercially exploited fish. Experiments after Conover & Munch (2002) reduced harvests rate but did not address the other issues.

# Detailed material and methods

## Founder populations

All individuals in the experiment were descendants of a single batch of wild-caught guppies (79 females and 25 males) collected from the Upper section of Yarra River situated on the north slope of the Northern Range Mountains in Trinidad, West Indies in 2009. Our collection site (10.73º N, -61.31º E) represents a low-predation population, as the site lacked the large predator *Crenicichla alta* and other cichlids but contained the smaller omnivore *Anablepsoides hartii* (=*Rivulus hartii*)*.* (Reznick *et al.*, 1996; Reznick and Travis, 2019). The wild-caught females produced up to seven broods in the laboratory. Because female guppies can store sperm from multiple males in the folds of their ovaries for months (Constantz 1984), the founding population probably represented a greater paternal diversity than the number of collected males would suggest, as in (López-Sepulcre *et al.* 2013).

Nine replicate populations were created in March 2010 with 140 F1-offspring of the wild parents. In October 2010, we started the selection experiment when the replicate populations had over 200 individuals and showed a broad age and size distribution (Table S1). When size-dependent harvest started on average the populations had 360 ± 73 individuals, a biomass of 61± 13 grams, and a mature population of 193 ± 94 individuals or 52 ± 28 grams. Note that the proportion of matured individuals at the beginning of the experiment could only be estimated for males, and thus it assumes that the proportion of mature individuals is equal for males and females.

| Table S1. Description of the populations in October 2010 when the harvest started. Sex and length come from a sample of 25% of individuals in the population. Mean length and standard deviation (SD) in milimeters, abundance is in number of individuals and biomass in grams. | | | | | | | | |
| --- | --- | --- | --- | --- | --- | --- | --- | --- |
| Population | Total abundance | Total  biomass | Mature abundance | Sex | Abundance in sample | Mean length  in sample | SD length in sample | Treatment |
| 1 | 508 | 59 | 197 | Female | 29 | 20.2 | 5.1 | Positive harvest |
|  |  |  |  | Male | 33 | 18.4 | 2.4 |  |
|  |  |  |  | Unsexed juvenile | 38 | 13.7 | 1.6 |  |
| 2 | 372 | 63 | 260 | Female | 33 | 21.9 | 5.6 | Random harvest |
|  |  |  |  | Male | 28 | 18.0 | 2.9 |  |
|  |  |  |  | Unsexed juvenile | 5 | 10.8 | 1.4 |  |
| 3 | 316 | 70 | 175 | Female | 39 | 23.6 | 4.4 | Negative harvest |
|  |  |  |  | Male | 33 | 18.3 | 1.5 |  |
|  |  |  |  | Unsexed juvenile | 30 | 12.6 | 1.3 |  |
| 4 | 372 | 70 | 237 | Female | 33 | 23.1 | 6.2 | Negative harvest |
|  |  |  |  | Male | 23 | 19.5 | 2.5 |  |
|  |  |  |  | Unsexed juvenile | 12 | 11.6 | 0.7 |  |
| 5 | 280 | 55 | 126 | Female | 16 | 25.2 | 8.0 | Random harvest |
|  |  |  |  | Male | 27 | 18.6 | 2.7 |  |
|  |  |  |  | Unsexed juvenile | 50 | 12.1 | 1.5 |  |
| 6 | 304 | 35 | 40 | Female | 23 | 27.2 | 4.5 | Positive harvest |
|  |  |  |  | Male | 5 | 20.8 | 1.6 |  |
|  |  |  |  | Unsexed juvenile | 24 | 12.2 | 1.5 |  |
| 7 | 428 | 58 | 270 | Female | 35 | 18.7 | 4.9 | Negative harvest |
|  |  |  |  | Male | 44 | 18.0 | 2.4 |  |
|  |  |  |  | Unsexed juvenile | 20 | 12.4 | 1.2 |  |
| 8 | 432 | 84 | 337 | Female | 21 | 25.6 | 5.8 | Random harvest |
|  |  |  |  | Male | 24 | 19.5 | 2.4 |  |
|  |  |  |  | Unsexed juvenile | 11 | 9.5 | 2.0 |  |
| 9 | 224 | 58 | 96 | Female | 18 | 19.8 | 5.4 | Positive harvest |
|  |  |  |  | Male | 21 | 18.4 | 2.2 |  |
|  |  |  |  | Unsexed juvenile | 7 | 12.9 | 1.6 |  |

- 1. *Maintenance and feeding*

The nine populations were maintained in 400-litre aquaria in a flow-through system at a constant temperature of 25 ºC (± 0.5ºC) and 12:12 light regime. They were fed twice a day with newly hatched brine shrimp, *Artemia salina* (brine shrimp eggs, Silver Star Artemia), in the morning and flakes (TetraMin, Tetra) in the afternoon. Food was rationed and equal for all populations, as the aim was to have compensatory response in the populations, but it was adapted to the average abundance in the populations and thus not constant throughout time. On average, each population received 3 ± 0.66 ml of *Artemia*, in the morning, and 0.36 ± 0.02 g of flakes, in the afternoon. Food ratio was increased to 4 ml of *Artemia* and 0.68 g of flakes when populations on average grew over 600 individuals (i.e., 100 g). This feeding regime resulted in each fish receiving on average 6.3 ± 1.4 µl of *Artemia* which is equivalent to a medium compared to the high and low food ratios used in the same population (Diaz Pauli 2012; Diaz Pauli & Heino 2013).

## 2.3 Harvesting protocol

Harvesting was conducted by removing all fish from a tank with a large hand net and then sorting fish into two groups, larger and smaller than 16 mm of SL. Length was assessed with a measuring board. The two size groups were further split randomly to 4–8 buckets, such that removing one bucket from the population gave the target harvest proportion. For example, to implement *P*=25% harvest proportion, we would split the sample in four buckets and harvest one of them, returning the other buckets to the tank. The non-harvested size group in Negative and Positive harvest regimes was split similarly, one bucket was used for the population census, and all fish were returned into the tank.

We interpret our experimental set-up as lacking a real control, as we believe such a control does not really exist. A “no harvest” treatment was not an option, as it would result in overcrowded populations and thus adaptation to overcrowding. *Random harvest* is unselective in size, but the imposed mortality is still evolutionary selective, as it favours fast life histories, like *Positive harvest*, but on a lower scale. We try to imitate wild conditions with *Negative harvest*, i.e., high mortality in juveniles and low in adults. However, “natural selection” in the lab certainly differs from wild natural selection. We therefore used the initial populations as our reference point, against which the harvest-induced changes are compared.

## 2.4 Phenotypic census:

During harvest events, we sampled 25% of the individuals from each population, representing one bucket of large and small fish each (see above). We refer to these fish as the census fish. These data also allowed to estimate total abundance and biomass of each population. *Census* fish were anaesthetised with a solution of MS-222 (0.3 g/L), sexed, measured (SL to the closest 0.5 mm), weighed (to the closest 0.01 g), and–in males only–assessed for maturation based on gonopodial (modified anal fin) development (Turner 1941b); female maturation stage cannot be assessed reliably non-invasively. Individuals too young for their sex to be visually determined were considered immature.

Because determination of female maturity and fecundity requires sacrificing them, we needed to sample extra females in cases where the harvest did not provide representative samples that could be dissected. Thus, some extra fish were sacrified in the Positive and Negative harvest treatments three times during the harvest phase 1 (19/09/2011, 18/09/2012, and 28/10/2013), and once during harvest phase 2 (02/03/2020). All females were dissected under the stereomicroscope to determine their maturation stage according to the development of their ova. Females were immature when only small ova and early-yolked ova were found (Haynes 1995). The number of embryos was counted as a measurement of fecundity.

To create life tables to estimate generation time, we used cohort marking to establish an estimate of individual age. This was done only during the harvest phase 1, from which we have 28 marked cohorts per population, one from each harvest. Twenty individuals between 11–12 mm SL were marked subcutaneously with a visible implant elastomer (VIE, Northwest Marine Technology, Inc.) on the dorsal region every 6 weeks in each population. The exact age of these fish was unknown, but the narrow length range lets us assume that they were born roughly at the same time. Newly born guppies, from isolated females in the lab, are on average 6.6 ± 0.5 mm SL (mean ± SD; pers. obs.) and reach 11–12 mm SL on average 37.2 ± 11.4 days old (i.e., approximately 5 weeks old) when individually maintained in a 2-litre tank. Thus, we assumed marked individuals to be 5–6 weeks old.

Cohort marking during harvest phase 1, also allowed us to estimate cohort average somatic growth and how this was affected by population biomass and abundance. This was done to obtain an indication of food availability, as all populations were supplied the same amount of food, but this did not necessary translated to equal per capita or per fish gram food availability.

## 2.5 Common-garden assessment

Four females (F0 generation) were taken out of each population (at random) and reared in common garden conditions in six occasions during the selection experiment: 1) at the start of the selection experiment (October 2010), 2) three times during harvest phase 1 – after 10, 20 and 28 harvest events or 54, 112, and 158 weeks experiencing selection (October 2011, December 2012 and October 2013, respectively), 3) once during the recovery phase – after 89 weeks of experiencing size-independent selection (December 2015), and 4) once during harvest phase 2 – after 74 weeks experiencing size-selection (August 2019). Because of logistic limitations, not all phases are represented equally. Harvest phase 1 is represented by three genetic assessments pooled together. Recovery and harvest phase 2 are only represented by one assessment. It should be noted that the last assessment was performed after the shortest exposure to the size-dependent harvest but also was influenced by longest previous exposure size-independent selection (200 weeks; Figure 1). F0 females were reared in isolation (2 L tank) after removing them from the large populations, kept until we got at least 10 offspring from them, and fed ad-lib.

Guppy females mate multiple times, and their offspring are likely a mixture of full-sibs and half-sibs (Evans & Magurran 2000). Tanks were check for offspring twice a day and F1 offspring were removed from the mothers’ tank as soon as detected. All siblings from the same brood were kept together until their sex could be identified and then separated in single-sex groups (average 6 fish/ 2L tank) to avoid sibling interbreeding. F1 fish were checked every week, and as soon as individual could be sexed there were removed from the common sibling tank (25-30 days old). When F1 fish are 3-4 months old, at least 4 families were created by randomly crossing F1 males and F1 females within the same population, but not crossing siblings ­– i.e., from the 12 possible crossings, we selected 4 randomly due to lab limitations. All F1 fish were fed ad-lib.

The aim was to obtain at least 3 F2 males and 3 F2 females per family. Thus, on average we had 15 ± 7 females and 17 ± 9 males per population and assessment. These 24 F2 individuals were reared in isolation (since 2 weeks old) in 2 L tanks flow-through system at a constant temperature of 25 ºC (± 0.5ºC) and 12:12 light regime. As before, tanks were checked twice daily for new-borns. All individuals were measured for SL and weighted every 2 weeks. The first 2 measurements of length were obtained from the photographs (at 0 and 2 weeks old). The brood was photographed as a group at birth and maintained as a group for 2 weeks, when it was photographed again. After that fish were isolated. Males were checked weekly for their maturation stage (assessed by the development of the gonopodium; Turner, 1941; Kallman and Schreibman, 1973; Schreibman and Kallman, 1977). Females, once approaching maturity (at 6 weeks old), were hosted with a single male each (from afternoon to next morning) and checked daily for new-borns. Males used for mating F2 females were from stocks created with extra F1 males from the size-selected populations. Mating was done between individuals of the same population but avoiding mating with related fish. Time at maturation was considered as the first time we observed new-borns and the last stage of development in the gonopodium according to (Turner 1941a), in females and males respectively. Both males and females were measured and weighted at maturation. Female early fecundity was estimated as number of offspring at first and second broods. Food was rationed since birth, and we followed Diaz Pauli & Heino (2013) feeding protocol, increasing the food ratio every 2 weeks. From birth to 4 weeks old we fed 5 µl Artemia/individual. From 4 weeks old, the food was increased to 10µl Artemia/individual, at 14 weeks old it was increased to 20µl Artemia/individual, and again at 20 weeks was increased to 25µl Artemia/individual. All quantities were measured volumetrically with a Hamilton syringe to the nearest 0.5 µL and provided twice (morning and afternoon) daily during harvest phase 1. After that, the same daily amounts were given once daily (midday) for the remaining of the experiment.

In the 6 assessments, females were measured until they gave birth to their second brood (average until they reached 230 ± 100 days old), while males were measured until they reached maturation (on average until they reached 137 ± 116 days old). In the last assessment from harvest phase 1, males and females were maintained until they died to estimate differences in senescence, in this assessment we also estimated lifetime fecundity. In the last 3 assessments, we have data on growth after maturation for both males and females. Thus, the growth curves for males are only during the immature stage in the first 3 assessments, while we have male juvenile and adult growth from the last 3 assessments. In the 4^th^ genetic assessment only, we studied senescence and kept the fish until they died (*N* = 229 fish). On average the fish in that assessment died at 648 days old, the oldest fish that died was 1375 days old. For those fish, we also have the lifetime fecundity: total number of broods, total number of offspring, and the date of the last brood. For the 6^th^ genetic assessment, we also collected data on lifetime fecundity as we as we kept females 100 days longer than the average age at last brood (330 days old, based on both 4^th^ and 6^th^ assessment), but fish were euthanized after that and thus data on senescence is not available.

The common garden experiment is a classical approach that can help determine whether evolutionary (i.e., genetic) change drives documented phenotypic divergences in wild populations (Thompson et al 2025, Huxman et al 2022, de Villemereuil et al 2016).

In common garden experiments individuals from different populations are reared under the same environmental conditions from very early life stages and ideally across several generations. As individuals develop and mature under common conditions, phenotypic differences that persist in this context should reflect underlying genetic differentiations rather than plastic responses to environmental conditions (Thompson et al 2025; Lambert et al., 2021). The rationale behind this protocol is that by growing individuals from different populations in a common environment one controls for the effects of phenotypic plasticity and, to a certain extent, genotype-by-environment interactions (de Villemereuil et al 2016). Common garden experiments have been used extensively with plants, fish invertebrates, birds, and small mammals (see references in de Villemereuil et al 2016 and Lambert et al., 2021). Therefore, the practical standard is that differences maintained after two generations under common conditions are assumed to have a genetic basis (Reznick and Travis, 2019).

# Statistical analyses

## 3.1 List of variables

**Dependent variables:**

1. Population demographics:
   1. Biomass and abundance
   2. Generation time
   3. Total and directly observed natural mortality
2. Phenotypic census
   1. Probability of being mature and L50
   2. Fecundity
   3. Cohort somatic growth
3. Common-garden assessment
   1. Probability of becoming mature and Lp50
   2. Individual growth: energy acquisition rate, *c,* and investment in reproduction, *r*
   3. First-broods fecundity and lifetime fecundity
   4. Lifespan

**Independent variables**

- **Positive harvest**: Size-dependent harvest where individuals larger than 16 mm were removed from the population, mimicking fisheries-like size-dependent harvest.
- **Negative harvest**: Size-dependent harvest where individuals shorter than 16 mm were removed from the population, opposite to fisheries-like size-dependent harvest.
- **Random harves**t: Size-independent harvest, where both large and short individuals were removed from the population.
- **Harvest phase 1**: Populations were harvested following one of the three size-dependent harvest regimes. It started in October 2010, and it lasted for 167 weeks.
- **Recovery phase**: All populations were harvested with Random harvest. The aim was assessing whether life-history traits will recover to initial conditions. It followed harvest phase 1 and lasted for 200 weeks.
- **Harvest phase 2**: Populations were again harvested following one of the three size-dependent harvest regimes. It followed the recovery phase and lasted for 153 weeks.

## 3.2 Population demographics I: Biomass and abundance

Total population biomass (in grams) and abundance (number of individuals) were estimated using a generalized mixed model with gaussian distribution, where population was included as random effect, while harvest regime, phase in the experiment and weeks in the experiment, together with their 3-way interaction were included as fixed effects.

## 3.3 Population demographics II: Generation time and phenotypic rate of change

Each population’s generation time was estimated using the life table method (Molles Jr 2002) which is based on birth and death schedules. Generation time per cohort is the average age of mothers of new-born offspring in such cohort. It was estimated as:

*T* = (∑ *x* * *l_x_* * *m_x_*)/ (∑ *l_x_* * *m_x_*) (Eq. 2)

where *x* is an age class (in cycles of 6 weeks), l_x_ is the proportion of the original cohort surviving until age *x* and *m_x_* is the per capita fecundity (number of embryos) of individuals of age *x*.

During harvest phase 1, we marked and followed 28 cohorts. Age was expressed in terms of cycles of six weeks, where *age 1* corresponded with the marking of 10–12 mm individuals. Observed survival probabilities were also estimated with the recapture as the proportion of recapture at time *t* relative to time *t-1*. Per capita fecundity for each age was estimated with a length- and population-dependent fecundity obtained with the fecundity data of harvested fish in three times during this phase (19/09/2011, 18/09/2012, and 28/10/2013). Generation time was not estimated for the recovery phase and harvest phase 2, as we did not have data on survival probabilities during those phases.

Rate of phenotypic change in male length at maturation was estimated using haldanes (Haldane 1949) from our time series in the harvest phases 1 and 2 with the regression approach (Hendry & Kinnison 1999). We regressed log(*x)*/*s*_p_ values from each harvest against the number of generations (*g*) since the first sampling, where *x is* male mean length at maturation and *s*_p_ is the pooled standard deviation. The regression was carried out with a linear model for each harvest regime, with the slope of the regression (and its 95% confidence intervals) being the estimated haldanes. We assumed equal generation time for both harvest phases to calculate rate of change. However, we were not able to estimate rate of change for recovery phase, as we lacked generation time. For females the rate of change was estimated as the difference in logged length at maturation between the beginning and the end of harvest phase 1, standardised by *s*_p_*g* and its 95% confidence intervals were estimated from two samples *t*-tests also standardised by *s*_p_*g* (Hendry & Kinnison 1999).

*3.4 Population demographics III: Total and directly observed natural mortality*

Our populations did not present predators and large parasites, as in wild guppy populations. However, our populations certainly presented competition for resources, cannibalism, and diseases by bacterial and viral infections (although visibly sick individuals were very seldom observed) that could contribute to natural extrinsic mortality. The mortality by cannibalism is only partially accounted for in our data, as we marked fish of 10-12 mm and above, and juveniles above 12 mm are not normally cannibalised (Schröder *et al.* 2009). Therefore, the natural extrinsic mortality estimated here only applies to large juveniles and to adults.

From the mark-recapture data obtained in harvest phase 1, we estimated total natural mortality rate (µ), given that the number of individuals at time *t*+1 (*N_t_*_+1_) equals the number of individuals at time *t* (*N_t_*) times the probability of surviving harvest (*P*_hs_) and natural factors (*P*_ns_). If *P*_ns_ is expressed as rate, *N_t_*_+1_ follows:

*N_t_*_+1_= *e* ^-µΔt^ * *P*_hs_ * *N_t_* (eq. 3)

To estimate µ, we modelled equation 3 using a generalized mixed model with Poisson distribution with harvest regime and total population abundance as fixed effect, *P*_hs_ * *N_t_* as offset, and population as random effect, where the intercept specified explicitly equals **to -µΔt and thus** total natural mortality rate (µ) equals **-intercept / Δt**.

In addition, we estimated natural mortality based on direct observations of dead fish. All dead fish we found in the tanks were recorded (tank, date, and sex). These dead individuals were mostly adults, as dead juveniles were ingested or degraded faster than adults and hence were not commonly found. We believe this is primarily a measure of senescence or adult intrinsic mortality after natural extrinsic mortality in earlier life stages (cannibalism) and harvest-related mortality have taken place. Thus, our proxy for adult natural intrinsic mortality was the number of dead individuals found relative to the total number of fish in the population for each 6 weeks interval. This estimate of natural mortality was modelled using generalized mixed model with zero-inflated gamma distribution, phase, time in phase (weeks), harvest regime and the tree-way interactions time×phase×harvest as fixed effects, while population was a random effect.

## 3.5. Phenotypic census of life history I: L50 and probability of being mature

Length at maturation was estimated as the length at which the probability of being mature is 50% (also referred to as L50). For males this was done from the census data throughout the whole selection experiment. For females, data came from the four occasions (3 from harvest phase 1 and 1 from harvest phase 2) when we invasively assessed maturation stage and fecundity. The probability of being mature was estimated using a generalized mixed model with binomial distribution. For males, the fixed effect variables were length, phase, time in phase (weeks), harvest regime and the tree-way interactions length×phase×harvest and time×phase×harvest, while the random factor was population. For females, the fixed effects were length, harvest event (4 events as categorical variable), harvest regime, and the interactions length×harvest and harvest event×harvest. The L50 was estimated with equation 1, where *c*_0_, *c*_1_, *c*_2_, etc. are the intercept and regression parameters for the different factors (*l*, length, *p*, phase, *h*, harvest regime, etc.) of the final model:

$L50= {-\left( c_{0}+c_{2}p+c_{3}h\ldots\right)}/{c_{1}l}$ (Eq. 1)

3.6. Phenotypic census of life history II: Fecundity

The smallest female with eggs was 13 mm in standard length, while the average length of a female with embryos was 22 ± 4.5 mm ($\overline{x}$± SD). Therefore, only females above 13 mm were included in the analysis. The number of eggs (independent of developmental stage) was modelled using generalized mixed model with negative binomial distribution and zero truncated data, as the data contained 40% of zeros. In the model female length (standardised relative to 16 mm and natural log transformed), Harvest regime and Harvest phase (phase 1 and phase 2) were included as fixed factors. Note that population was not included as random factor, but length was included as dispersion parameter to to account for heteroscedastictity. This gave a non-significant Kolmogorov-Smirnov Goodness of Fit Test. The model including population as a random effect showed a low variance due to population (0.008) and qualitatively equal results as the model without random effect.

## 3.7. Phenotypic census of life history III: Cohort somatic growth

Cohort growth in length was estimated with a gaussian distribution model, where standard length was the response variable, while age, age quadratic, sex, harvest regime, and biomass were included as fixed effects, and population as random effect. In addition, the 4-way interaction of age (and age quadratic) with sex, harvest regime, and biomass were also included.

## 3.8. Common-garden assessment of life history I: Lp50 and probability of becoming mature

Length and age at maturation was estimated as the length at which the probability of becoming mature is 50% (also referred as Lp50) following (Heino *et al.* 2002) for males and females using data from our six common garden experiments. With repeated measurements per individual, we could tell apart individuals that just became mature from others already mature individuals. The six common garden experiments are grouped into the three different phases in the experiment: harvest phase 1, recovery phase and harvest phase 2. However, our first common garden experiment was done before the size-selective harvest started and hence we have a fourth initial phase.

The probability of becoming mature was tested using generalized mixed model with binomial distribution. For males, the fixed effect variables were length, age, phase, harvest regime, the tree-way interaction length-phase-harvest, and the time interval between measurements was logged and included as offset to control for differences in interval length, while the random factor was individual fish Id. For females, the same model structure was used, except that the tree-way interaction was age-phase-harvest. The Lp50 was estimated with equation 3, where *c*_0_, *c*_1_, *c*_2_, etc. are the intercept and regression parameters for the different factors (*l*, length, *a*, age, *p*, phase, *h*, harvest regime, etc.) of the final model:

$Lp50= {-\left( c_{0}+c_{2}a+c_{3}p+c_{4}h\ldots\right)}/{c_{1}l}$ (Eq. 4)

## 3.9. Common-garden assessment of life history II: Individual growth, energy acquisition rate, c, and investment in reproduction, r

We modelled individual growth for males and females separately using the Quince-Boukal biphasic growth model (Boukal *et al.* 2014; Quince *et al.* 2008) estimated with the ‘nlme’ R package (version 3.1-153; Pinheiro *et al.*, 2021). The model provides a mechanistic description of somatic growth pre- and post-maturation, based on the principles of allometry and energy allocation. Surplus energy acquisition rate, which is equal to maximal potential somatic growth, is related to somatic weight, *W*, by the coefficient, *c,* and the allometric exponent, β, as in equation 4:

$\frac{dW}{da}=cW^{\beta}$ (Eq. 5)

The allometric exponents β were estimated from age, *a*, and weight, *W*, data of immature individuals only. In addition, we limited the age range to the average age at maturation in males (63 ± 11 days old, $\overline{x}$± SD), while in females it was limited to 101 days old, which is the average age at birth of the first brood minus 40 days of gestation. Gestation time was estimated as the average inter-brood interval (40 ± 23 days, $\overline{x}$± SD; Auer, 2010). Both measures were done to ensure we estimated β with length and weight data from fish that presented the maximal potential somatic growth before they started investing in maturation.

The biphasic growth model assumes that juveniles allocate all surplus energy into growth (i.e., the investment in reproduction *r*_a_ = 0), and thus, the juvenile growth curve for length, at age, *a*, *L*_a_, follows equation 5.

$L_{a}= \sqrt[(1-\beta)\alpha]{L_{0}^{(1-\beta)\alpha}+ c(1-\beta)b^{-(1-\beta)}a}$ (Eq. 6)

While the adult growth rate considers the investment in reproduction, *r*, of the mature individuals, whose age is larger than their age at maturation (*a* > *a*_mat_) and the length-age growth curve follows equation 6:

$L_{a}= \sqrt[(1-\beta)\alpha]{R^{a-a_{mat}}(L_{0}^{(1-\beta)\alpha}+Ha_{mat})+\frac{RH}{1-R}(1-R^{a-a_{mat}})}$ (Eq. 7)

where *H* = *c*(1 − β) *b*^−(1 − β)^ and *R* = 1/[1 + (1 − β) *r*], assuming the conversion factor between somatic and gonadic investment, *q*, in (Boukal *et al.* 2014) to be 1 as in (Diaz Pauli *et al.* 2019; Minte-Vera *et al.* 2016). *L*_0_ is length at birth and equal to 6.5 mm (± 0.3 SD) for both males and females, as estimated average from our data. There were no differences in length at birth between sexes (*t* = 0.55, *P* = 0.584) and among harvest regimes (*t* = 0.91, *P* = 0.397).

The coefficient, *b*, and exponent, α, of the allometric relationship of weight, *W*, with length, *L*, were estimated with the natural-logged length and natural-logged weight data prior running the biphasic growth model. This was done for males and females separately to assess sex differences in allometry. For these parameters we tested whether harvest regime, phase in the experiment, and their interaction had an effect using a linear mixed effect model with logged length (standardised to 16 mm, i.e., size at selection threshold) and weight, and individual fish Id as random effect. Thus here, the estimated *b* refers to an individual of 16 mm, which is the size at selection. It should be noted that *b* and α could not be estimated for the initial phase as we did not have weight measurements for all individuals. Thus, we used for the initial phase the same values as those estimated for harvest phase 1, which gave a better fit of the data than if we had used the same values as those estimated for recovery phase. Phase in the experiment influenced the weight-length relationship affecting both *b* and α in males (Table S2b) and females (Table S2c). On the one hand, both males and females presented the highest values of *b* during harvest phase 1 and the lowest during harvest phase 2 (Table S2a). On the other hand, alpha was highest during the recovery phase and lowest for harvest phase 1 in males and females (Table S1a). Together this resulted in the recovery phase having the steepest length-weight relationship, and harvest phase 1 having the least steep curve. In addition, α was also affected by harvest regime but only in females (Table S1c), which resulted in females exposed to Positive harvest having larger α – thus steeper length-weight curves – than those exposed to Negative harvest (Table S2a; Estimate = -0.03, *t* value = -2.85, *P* value = 0.004).

Finally, the coefficient in the energy acquisition rate, *c*, and investment in reproduction, *r*, were estimated with the biphasic model using the observed repeated measurements per individual for length, *L*, age, *a*, age at maturation, *a*_mat_, and fixing the parameters estimated earlier, as it was not possible to estimate all parameters at the same time. **We tested whether harvest regime, phase in the experiment, and their interaction affected *c* and *r.*** Id was random effect for both parameters. Note that for females *a*_mat_ was age at first parturition minus 40 days of gestation, as explained above. For males and females, the fixed parameters are represented in Table S2a.

| Table S2a. Values of biphasic growth model parameters fixed in the final estimation of coefficient in the energy acquisition rate, *c*, and investment in reproduction, *r.* | | | |
| --- | --- | --- | --- |
| Parameter |  | Males | Females |
| *L*_0_  Length at birth |  | 6.5 mm | 6.5 mm |
| β  energy acquisition rate exponent |  | 0.70 | 0.30 |
| α  weight-length  allometric exponent | Initial | 2.95 | Random = 3.05, Negative = 3.03, Positive = 3.06 |
|  | Harvest 1 | 2.95 | Random = 3.05, Negative = 3.03, Positive = 3.06 |
|  | Recovery | 3.51 | Random = 3.35, Negative = 3.32, Positive = 3.36 |
|  | Harvest 2 | 3.13 | Random = 3.13, Negative = 3.11, Positive = 3.14 |
| *b*  weight-length  coefficient | Initial | 1.60 g mm^-b^ | 1.62 g mm^-b^ |
|  | Harvest 1 | 1.60 g mm^-b^ | 1.62 g mm^-b^ |
|  | Recovery | 1.59 g mm^-b^ | 1.44 g mm^-b^ |
|  | Harvest 2 | 1.42 g mm^-b^ | 1.38 g mm^-b^ |

| Table S2b. Results from natural-logged ([log]) weight-length relationship model for males. Length was standardised to 16 mm (Length std). | | | | |
| --- | --- | --- | --- | --- |
| Predictors | Estimates | CI | *t* value | *P* value |
| (Intercept):  Harvest 1, 16 mm | -2.3 | -2.31 – -2.29 | -445.54 | **<0.001** |
| Length std [log] | 2.95 | 2.91 – 2.99 | 156.8 | **<0.001** |
| Recovery | -0.02 | -0.03 – 0.00 | -1.54 | 0.124 |
| Harvest 2 | -0.12 | -0.13 – -0.10 | -15.79 | **<0.001** |
| Length std [log] * Recovery | 0.56 | 0.49 – 0.63 | 15.61 | **<0.001** |
| Length std [log] * Harvest 2 | 0.18 | 0.14 – 0.23 | 7.78 | **<0.001** |

| Table S2c. Results from natural-logged ([log]) weight-length relationship model for females. Length was standardised to 16 mm (Length std). | | | | |
| --- | --- | --- | --- | --- |
| Predictors | Estimates | CI | *t* value | *P* value |
| (Intercept):  *Random, Harvest 1, 16 mm* | -2.29 | -2.30 – -2.28 | -485.4 | **<0.001** |
| Length std [log] | 3.05 | 3.03 – 3.07 | 322.11 | **<0.001** |
| Negative | 0 | -0.01 – 0.01 | 0.23 | 0.82 |
| Positive | 0 | -0.01 – 0.01 | -0.64 | 0.523 |
| Recovery | -0.12 | -0.13 – -0.11 | -17.93 | **<0.001** |
| Harvest 2 | -0.16 | -0.17 – -0.15 | -31.9 | **<0.001** |
| Length std [log] * Negative | -0.02 | -0.04 – 0.00 | -1.92 | 0.055 |
| Length std [log] * Positive | 0.01 | -0.01 – 0.03 | 0.91 | 0.361 |
| Length std [log] * Recovery | 0.3 | 0.27 – 0.33 | 21.04 | **<0.001** |
| Length std [log] * Harvest 2 | 0.08 | 0.06 – 0.10 | 7.77 | **<0.001** |

## 3.10. Common-garden assessment of life history III: First-broods fecundity and lifetime fecundity

The number of offspring born in a female’s first and second brood were analysed together. We considered data from our six common garden experiments and each full model with Poisson distribution included female length at brood birth (natural logged and standardised relative to 16 mm), harvest regime, experimental phase, and their interaction as fixed effects, while population and brood number nested within population were included as random effects. We also recorded lifetime fecundity as the total number of offspring in all broods until death for two out of the six common garden experiments. These were common garden experiments number 4 and 6, representing harvest phase 1 and harvest phase 2, respectively. The full model had population as random effect and the same fixed as above, excluding length as fixed effect, as this was not available for the birth of all broods, but presented a negative binomial distribution, due to overdispersion with Poisson distribution.

## 3.11. Common-garden of life history IV: lifespan

The effect of harvest regime and sex on lifespan was tested from data of the 4^th^ common garden experiment, representing the end of harvest phase 1. We estimate four different lifespans: 1) age at death due to natural causes was used as a proxy for total lifespan, 2) adult life span was the time from maturation until death, 3) reproductive lifespan was the time between maturation and birth of last brood, and 4) post-reproductive lifespan was the time between birth of last brood and natural death. The first two models had Harvest regime, sex, and their interaction were included as fixed effects, while Population was included as random effect. The last two lifespans were only estimated for females for which we had data on age at parturition of their last brood. Therefore, in the models only Harvest regime was included as fixed effect. All models followed Gaussian distribution. Age at death was square-root-transformed to fulfil model requirements.

# Results on detailed trends throughout the experiment

## 4.1. Harvesting has direct demographic effects that depend on harvest regime

### 4.1.1. Biomass and abundance

Figure S1. Average a) abundance and b) biomass per harvest regime throughout the size-selection experiment. Different harvest phases are shown with white areas, while the recovery phase is marked with a dark grey area, and the break phase with pale grey area. The three harvest regimes are Negative size-dependent harvest (blue triangles), Random harvest (white circles), and Positive size-dependent harvest (orange inverted triangles).

| Table S3a*.* Results from the biomass model with Gaussian distribution. Estimates and confidence intervals (CI) in grams. | | | | |
| --- | --- | --- | --- | --- |
| Predictors | Estimates | CI | *z* value | *P* value |
| (Intercept): *Random, Harvest phase 1* | 82.65 | 77.81 – 87.49 | 33.47 | **<0.001** |
| Negative | 27.57 | 20.72 – 34.41 | 7.89 | **<0.001** |
| Positive | -4.01 | -10.85 – 2.83 | -1.15 | 0.251 |
| Recovery phase | -21.84 | -29.76 – -13.91 | -5.4 | **<0.001** |
| Harvest phase 2 | -54.12 | -61.11 – -47.12 | -15.16 | **<0.001** |
| Weeks in phase | -0.12 | -0.17 – -0.08 | -4.96 | **<0.001** |
| Negative * Recovery phase | -25.99 | -37.20 – -14.79 | -4.55 | **<0.001** |
| Positive * Recovery phase | 4.39 | -6.82 – 15.60 | 0.77 | 0.442 |
| Negative * Harvest phase 2 | -23.62 | -33.51 – -13.73 | -4.68 | **<0.001** |
| Positive * Harvest phase 2 | 5.88 | -4.01 – 15.77 | 1.16 | 0.244 |
| Negative * Weeks in phase | -0.2 | -0.27 – -0.13 | -5.48 | **<0.001** |
| Positive * Weeks in phase | -0.04 | -0.11 – 0.03 | -1.09 | 0.277 |
| Recovery phase * Weeks in phase | 0.1 | 0.02 – 0.18 | 2.47 | **0.013** |
| Harvest phase 2 * Weeks in phase | 0.34 | 0.26 – 0.42 | 8.22 | **<0.001** |
| Negative * Recovery phase * Weeks in phase | 0.2 | 0.09 – 0.31 | 3.5 | **<0.001** |
| Positive * Recovery phase * Weeks in phase | 0.05 | -0.06 – 0.16 | 0.85 | 0.394 |
| Negative * Harvest phase 2 * Weeks in phase | 0.23 | 0.11 – 0.34 | 3.83 | **<0.001** |
| Positive * Harvest phase 2 * Weeks in phase | 0.0040 | -0.11 – 0.12 | 0.07 | 0.941 |

| Table S3b*.* Results from the abundance model with Gaussian distribution. Estimates and confidence intervals (CI) in grams. | | | | |
| --- | --- | --- | --- | --- |
| Predictors | Estimates | CI | *z* value | *P* value |
| (Intercept): *Random, Harvest phase 1* | 316.38 | 204.44 – 428.32 | 5.54 | **<0.0001** |
| Negative | 350.75 | 192.44 – 509.06 | 4.34 | **<0.0001** |
| Positive | 183.39 | 25.08 – 341.70 | 2.27 | **0.0232** |
| Recovery phase | 202.93 | 128.89 – 276.97 | 5.37 | **<0.0001** |
| Harvest phase 2 | 72.36 | 7.01 – 137.71 | 2.17 | **0.03** |
| Weeks in phase | 0.98 | 0.52 – 1.44 | 4.17 | **<0.0001** |
| Negative * Recovery phase | -555.41 | -660.12 – -450.70 | -10.4 | **<0.0001** |
| Positive * Recovery phase | -223.19 | -327.90 – -118.48 | -4.18 | **<0.0001** |
| Negative * Harvest phase 2 | -320.04 | -412.45 – -227.62 | -6.79 | **<0.0001** |
| Positive * Harvest phase 2 | -155.98 | -248.39 – -63.56 | -3.31 | **0.0009** |
| Negative * Weeks in phase | -3.07 | -3.72 – -2.42 | -9.22 | **<0.0001** |
| Positive * Weeks in phase | -1.37 | -2.02 – -0.72 | -4.12 | **<0.0001** |
| Recovery phase * Weeks in phase | -1.2 | -1.94 – -0.46 | -3.18 | **0.0015** |
| Harvest phase 2 * Weeks in phase | 2.12 | 1.35 – 2.88 | 5.44 | **<0.0001** |
| Negative * Recovery phase * Weeks in phase | 4.12 | 3.07 – 5.17 | 7.71 | **<0.0001** |
| Positive * Recovery phase * Weeks in phase | 2.32 | 1.28 – 3.37 | 4.35 | **<0.0001** |
| Negative * Harvest phase 2 * Weeks in phase | 1.68 | 0.60 – 2.76 | 3.05 | **0.0023** |
| Positive * Harvest phase 2 * Weeks in phase | 2.45 | 1.37 – 3.53 | 4.45 | **<0.0001** |

Figure S2. Total biomass yield and mean individual weight throughout the size-selection experiment: at the start of the experiment (1^st^ panel) and at the end of harvest phase 1 (2^nd^ panel), recovery phase (3^rd^ panel), and harvest phase 2 (4^th^ panel) for the three harvest regimes, Negative size-dependent harvest (blue triangles), Random harvest (white circles), and Positive size-dependent harvest (orange inverted triangles).

### 4.1.2. Truncation of size structure

| Table S4a*.* Results from length above 16 mm model with Gaussian distribution in males. Estimates and confidence intervals (CI) in mm. | | | | |
| --- | --- | --- | --- | --- |
| Predictors | Estimates | CI | *z*-value | *P*-value |
| (Intercept): *Random, Harvest phase 1, 0 weeks* | 18.90 | 18.64 – 19.16 | 143.11 | **<0.0001** |
| Negative | -0.81 | -1.05 – -0.56 | -6.46 | **<0.0001** |
| Positive | -0.76 | -1.17 – -0.35 | -3.65 | **0.0003** |
| Recovery phase | -0.99 | -1.17 – -0.81 | -10.58 | **<0.0001** |
| Harvest phase 2 | -1.81 | -1.98 – -1.63 | -20.10 | **<0.0001** |
| Weeks in phase | -0.005 | -0.006 – -0.003 | -7.45 | **<0.0001** |
| Negative * Recovery phase | 1.74 | 1.48 – 1.999 | 13.14 | **<0.0001** |
| Positive * Recovery phase | 0.75 | 0.46 – 0.97 | 5.52 | **<0.0001** |
| Negative * Harvest phase 2 | 0.82 | 0.58 – 1.054 | 6.68 | **<0.0001** |
| Positive * Harvest phase 2 | 0.35 | 0.10 – 0.595 | 2.78 | **0.0054** |
| Negative * Weeks in phase | 0.005 | 0.003 – 0.007 | 5.72 | **<0.0001** |
| Positive * Weeks in phase | 0.002 | -0.000 – 0.003 | 1.79 | 0.0732 |
| Recovery phase * Weeks in phase | 0.004 | 0.002 – 0.006 | 4.03 | **0.0001** |
| Harvest phase 2 * Weeks in phase | 0.004 | 0.001 – 0.006 | 3.36 | **0.0008** |
| Negative * Recovery phase * Weeks in phase | -0.01 | -0.013 – -0.007 | -7.54 | **<0.0001** |
| Positive * Recovery phase * Weeks in phase | -0.003 | -0.006 – -0.000 | -2.27 | **0.0232** |
| Negative * Harvest phase 2 * Weeks in phase | -0.002 | -0.005 – 0.001 | -1.35 | 0.1774 |
| Positive* Harvest phase 2 * Weeks in phase | -0.004 | -0.007 – -0.001 | -2.64 | **0.0084** |

| Table S4b*.* Results from length above 16 mm model with Gaussian distribution in females. Estimates and confidence intervals (CI) in mm | | | | |
| --- | --- | --- | --- | --- |
| Predictors | Estimates | CI | *z*-value | *P*-value |
| (Intercept): *Random, Harvest phase 1, 0 weeks* | 25.59 | 24.67 – 26.52 | 53.98 | **<0.0001** |
| Negative | -3.73 | -4.32 – -3.14 | -12.38 | **<0.0001** |
| Positive | -2.95 | -4.50 – -1.40 | -3.73 | **0.0002** |
| Recovery phase | -4.11 | -4.53 – -3.70 | -19.30 | **<0.0001** |
| Harvest phase 2 | -6.16 | -6.56 – -5.76 | -30.20 | **<0.0001** |
| Weeks in phase | -0.02 | -0.02 – -0.02 | -13.11 | **<0.0001** |
| Negative * Recovery phase | 6.65 | 6.06 – 7.23 | 22.17 | **<0.0001** |
| Positive * Recovery phase | 3.76 | 3.18 – 4.35 | 12.57 | **<0.0001** |
| Negative * Harvest phase 2 | 3.01 | 2.49 – 3.53 | 11.29 | **<0.0001** |
| Positive * Harvest phase 2 | 2.13 | 1.56 – 2.69 | 7.37 | **<0.0001** |
| Negative * Weeks in phase | 0.03 | 0.03 – 0.03 | 15.09 | **<0.0001** |
| Positive * Weeks in phase | 0.02 | 0.01 – 0.02 | 5.81 | **<0.0001** |
| Recovery phase * Weeks in phase | 0.02 | 0.01 – 0.02 | 8.73 | **<0.0001** |
| Harvest phase 2 * Weeks in phase | 0.02 | 0.02 – 0.03 | 8.19 | **<0.0001** |
| Negative * Recovery phase * Weeks in phase | -0.05 | -0.05 – -0.04 | -15.77 | **<0.0001** |
| Positive * Recovery phase * Weeks in phase | -0.02 | -0.03 – -0.02 | -6.94 | **<0.0001** |
| Negative * Harvest phase 2 * Weeks in phase | -0.02 | -0.02 – -0.01 | -5.74 | **<0.0001** |
| Positive* Harvest phase 2 * Weeks in phase | -0.02 | -0.03 – -0.02 | -6.57 | **<0.0001** |

| Table S4c. Elasticity estimates for a change in average length above 16 mm in males and females relative to a change in biomass for three transitions: 1) from start of experiment (start) to end of harvest phase 1 (H1), 2) from end of recovery phase to end of harvest phase 2 (H2), and 3) from start of experiment to end of experiment (H2), for the three harvest regimes. | | | | |
| --- | --- | --- | --- | --- |
|  | Transition | Negative | Random | Positive |
| Length above 16mm Males | Start – H1 | -0.008 | 0.161 | 0.081 |
|  | Recovery – H2 | -0.065 | -0.418 | -14.976 |
|  | Start – H2 | 0.104 | 0.413 | 0.425 |
| Length above 16mm Females | Start – H1 | -0.176 | 0.491 | 0.148 |
|  | Recovery – H2 | -0.055 | -0.786 | -33.254 |
|  | Start – H2 | 0.151 | 0.939 | 0.941 |

### *4.1.3*. *Non-harvest mortality:* Total and directly observed natural mortality

| Table S5a. Results from total natural mortality model with Poisson distribution where total natural mortality µ is - Log-estimate / 6 weeks interval in week^-1^. The value from the intercept refers to the estimate in the Random harvest regime, while in Negative and Positive harvest indicate the difference in mortality rate with Random harvest | | | | | |
| --- | --- | --- | --- | --- | --- |
| Predictors | Log-estimate | CI | *z* value | *P* value |  |
| (Intercept): *Random* | -0.25 | -0.29 – -0.22 | -15 | **<0.0001** |  |
| Negative | 0.01 | -0.03 – 0.06 | 0.58 | 0.5649 |  |
| Positive | -0.18 | -0.23 – -0.12 | -6.63 | **<0.0001** |  |
| Abundance | 0.01 | -0.01 – 0.03 | 0.8 | 0.4215 |  |

| Table S5b. Results from directly observed natural mortality model with gamma and zero inflated distribution. Note the zero-inflated model shows probability of having zero mortality |
| --- |

| Predictors | Estimates | CI | *z* value | *P* value |
| --- | --- | --- | --- | --- |
| Count Model |  |  |  |  |
| (Intercept): *Random, Harvest phase 1, 0 weeks in phase* | 0.01 | 0.006 – 0.008 | -61.78 | **<0.0001** |
| Negative | 1.03 | 0.88 – 1.21 | 0.35 | 0.726 |
| Positive | 0.71 | 0.59 – 0.84 | -3.90 | **0.000** |
| Recovery | 0.25 | 0.19 – 0.32 | -10.27 | **<0.0001** |
| Harvest 2 | 0.34 | 0.26 – 0.44 | -7.92 | **<0.0001** |
| Weeks in phase | 1.00 | 0.995 – 0.997 | -6.02 | **<0.0001** |
| Negative *Recovery | 1.42 | 1.05 – 1.92 | 2.25 | **0.025** |
| Positive *Recovery | 2.04 | 1.51 – 2.76 | 4.63 | **<0.0001** |
| Negative * Harvest 2 | 2.92 | 2.23 – 3.83 | 7.77 | **<0.0001** |
| Positive * Harvest 2 | 2.48 | 1.88 – 3.27 | 6.40 | **<0.0001** |
| Recovery * Weeks in phase | 1.01 | 1.01 – 1.01 | 8.12 | **<0.0001** |
| Harvest 2 * Weeks in phase | 1.00 | 0.998 – 1.003 | 0.31 | 0.757 |
| (Intercept) | 1.99 | 1.93 – 2.05 |  |  |
| Zero-Inflated Model |  |  |  |  |
| (Intercept): *Random, Harvest phase 1, 0 weeks in phase* | 0.51 | 0.33 – 0.77 | -3.18 | **0.002** |
| Negative | 0.72 | 0.45 – 1.16 | -1.34 | 0.181 |
| Positive | 1.31 | 0.81 – 2.12 | 1.11 | 0.266 |
| Recovery | 1.24 | 0.68 – 2.25 | 0.71 | 0.478 |
| Harvest 2 | 9.28 | 5.28 – 16.33 | 7.73 | **<0.0001** |
| Weeks in phase | 1.01 | 1.005 – 1.012 | 5.45 | **<0.0001** |
| Negative *Recovery | 1.78 | 0.95 – 3.32 | 1.81 | 0.070 |
| Positive *Recovery | 0.75 | 0.4 – 1.39 | -0.93 | 0.355 |
| Negative * Harvest 2 | 0.52 | 0.29 – 0.93 | -2.22 | **0.026** |
| Positive * Harvest 2 | 0.32 | 0.18 – 0.56 | -3.95 | **0.000** |
| Recovery * Weeks in phase | 1.00 | 0.99 – 1 | -0.74 | 0.458 |
| Harvest 2 * Weeks in phase | 0.98 | 0.97 – 0.98 | -8.35 | **<0.0001** |

| Table S5c. Harvest regime pairwise comparison of directly observed natural mortality. Pairwise comparison between beginning and end of harvest phase 1. Degrees of freedom = 1656. | | | | |
| --- | --- | --- | --- | --- |
| Contrast | Ratio | SE | *t* ratio | *P* value |
| Start harvest phase 1 | | | | |
| Random / Negative | 1.41 | 0.13 | 3.90 | **0.0001** |
| Random / Positive | 0.97 | 0.08 | -0.35 | 0.726 |
| Negative / Positive | 0.69 | 0.06 | -4.37 | **<0.0001** |
| End harvest phase 1 | | | | |
| Random / Negative | 1.41 | 0.13 | 3.90 | **0.0001** |
| Random / Positive | 0.97 | 0.08 | -0.35 | 0.726 |
| Negative / Positive | 0.69 | 0.06 | -4.37 | **<0.0001** |
| End recovery phase | | | | |
| Random / Negative | 0.69 | 0.09 | -2.92 | **0.004** |
| Random / Positive | 0.69 | 0.09 | -2.88 | **0.004** |
| Negative / Positive | 0.99 | 0.13 | -0.07 | 0.946 |
| End harvest phase 2 | | | | |
| Random / Negative | 0.57 | 0.06 | -5.07 | **<0.0001** |
| Random / Positive | 0.33 | 0.04 | -9.99 | **<0.0001** |
| Negative / Positive | 0.58 | 0.06 | -5.56 | **<0.0001** |
| Harvest phase 1 | | | | |
| Start/end | 1.97 | 0.22 | 6.02 | **<0.0001** |

| Table S5d. Elasticity estimates for a change in directly observed natural mortality relative to a change in biomass for three transitions: 1) from start of experiment (start) to end of harvest phase 1 (H1), 2) from end of recovery phase to end of harvest phase 2 (H2), and 3) from start of experiment to end of experiment (H2), for the three harvest regimes. | | | |
| --- | --- | --- | --- |
| Transition | Negative | Random | Positive |
| Start – H1 | 1.487 | 2.973 | 2.222 |
| Recovery – H2 | 5.033 | -3.299 | -6.084 |
| Start – H2 | 1.022 | 3.245 | 1.403 |

## 4.2. Size-dependent harvest affects phenotypic traits differently under different harvest regimes

### 4.2.1. Mean size at maturation: L50

Length at which 50% of individuals were mature (L50) changed throughout the course of the experiment, indicating that the probability of maturing has changed over time in both sexes (Figure S3, Table S6a and S7a). The patterns of changed can be better described for males, for which we have more data. L50 decreased in males for all harvest regimes in harvest phase 1 and recovery phase. During harvest phase 2, we observed differences among the harvest regimes. Males exposed to Positive harvest decreased the length at 50% maturation with time exposed to harvest phase 2, while males exposed to Negative Harvest presented increasing length at 50% maturation with time.

Throughout harvest phase 1, Negative and Positive harvest did not differ in the odds of maturation in either sex. Only Random harvest for males initially differed from the other two in maturation probability, but by midway in the phase, it equalized with the rest (Table S6b). This initial difference is most likely due to arbitrary differences in the founder populations. Females exposed to Negative harvest had higher probability of maturing by week 48 (Table S8b), but again this difference disappeared by the end of harvest phase 1. During harvest phase 1, the different harvest regime did not seem to affect the probability of maturation differently, instead other selective forces seem to reduce the probability of maturation in all populations and equalize them. Biomass and abundance decreased throughout harvest phase 1 (Table S3a-b), which could have led to more available resources, higher probability of being mature, and thus lower L50. However, the decrease in biomass was steepest in Negative harvest and slowest in Positive harvest, and both harvest regimes experienced a similar decrease in L50. Moreover, elasticity estimates indicated that L50 for males and females was not sensitive to biomass (e~0) and that all harvest regimes responded similarly to it (Table S6c). Therefore, biomass does not seem the sole driver of L50 decrease in males for both harvest regimes. Such selective forces are probably acclimatation to the laboratory conditions. Thus, it seems that the 4 months of acclimation we allowed (from creation of populations to the start of the harvest regimes) was not enough. Despite that, Positive and Random harvest presented a significant phenotypic rate of decrease in male length at maturation of *h*_p (0.73)_ = -1.37[-2.70, -0.04; *P*-value = 0.044], and *h*_p (0.58)_ = -3.17 [-4.46, -1.87; *P*-value < 0.001], respectively, while there was not a significant change for the Negative harvest *h*_p (0.56)_ = 0.33 [-1.81, 2.46; *P*-value = 0.756] (Figure S4).

During the recovery phase, when all populations were exposed to the same size-independent harvest (Random harvest), all populations presented an increase in probability of maturing. The Random harvest lines presented the highest increment in odds ratio per time step in the recovery phase (Odds ratio between time step = 0.982), while the Negative harvest lines presented the lowest increase in maturation probability (Odds ratio between time step = 0.996), but very similar to the Positive harvest lines (Odds ratio between time step = 0.997). The Random harvest lines did not experience a change in harvest regime between harvest phase 1 and recovery phase, while the other lines did. During most of this phase Random and Positive harvest did not differ from each other but presented higher odds of maturing than Negative harvest (Table S6a).

Finally, at the beginning of the harvest phase 2 harvest regimes did not differ from each other. At midway during the experimental phase, males from Positive harvest presented highest odds of maturation, represented by the lowest length at 50% maturation, while Negative harvest presented the lowest odds of maturation (Table S6b). By the end of the experiment males exposed to Positive harvest had odds of maturing 100 times that of males exposed to Negative harvest (Table S6b), while they had 13 times the odds of maturing of males exposed to Random harvest. The odds of maturing in a male at the end of the harvest phase 2 compared to the beginning were 5 times more for the Positive harvest line, resulting in a shorter L50 (Figure S3) and a phenotypic rate of decrease of mean length of mature males of h_p(0.71)_ = -2.64 [-3.90, -1.39; *P*-value = 0.0003; Figure S4]. In the negative harvest, males at the end of the harvest phase 2 had 11 times less odds of maturing that those at the beginning, this resulted in an increase in L50 (Figure S3) and a phenotypic rate of change of mean length of mature males of h_p(0.47)_ = 2.86 [0.44, 5.27; *P*-value = 0.023]. The Random harvest line did not show a significant phenotypic change for mean length of mature males (h_p(0.53)_ = -0.28 [-2.29, 1.73; *P*-value = 0.773]; Figure S4), but the odds of maturing between the end and beginning of the phase were 1.7 times less and hence an increase in L50 (Figure S2). Male L50 was only sensitive to biomass change at the end of the experiment relative to the Recovery phase for Positive harvest. In all other cases, male L50 was insensitive to biomass (Table S6c). Between recovery and harvest phase 2, the increase in biomass was linked to a decrease in L50. However, it should be noted that biomass between end of recovery and harvest 2 phases did not significantly differ in populations exposed to Positive harvest (Table S3a). Therefore, such insignificant biomass change is unlikely the driver of the observed significant decrease in L50 and the odd of maturing 50 times larger by the end of harvest phase 2 relative to end of recovery phase (Figure S3; Table S6b). Females exposed to Positive harvest at the end of harvest phase 2 presented 12 and 6 times the odds of maturing relative to females exposed to Negative harvest and Random Harvest, respectively, as observed by lower L50 in females exposed to Positive harvest (Figure S3; Table S7b). Female L50 was elastic relative to biomass change at the end of the experiment relative to the start of the experiment only for Positive harvest, while all other transitions were insensitive (Table S6c). Between the start and the end of the experiment a decrease in biomass of 20 g was linked with a decrease in L50 of 4 mm (Figure 3). However, a larger biomass decrease (49 g) in Negative harvest during the same transition (Table S3a) was not linked to a similar decrease in L50. Therefore, biomass might not be the sole driver of change in L50, and it affected differently the harvest regimes.

|  |
| --- |
| Figure S3. Length at which the probability of being mature is 50% (L50), 25% (L25) and 75% (L75) for males (L50=symbols, L25=dashed lines, L75=dotted lines) and females (L50=symbols, L25 and L75=whiskers; jittered to avoid overlap), for each experimental phase: harvest phase 1 is in the left panel, recovery phase in the central shaded panel, and harvest phase2 in the right panel; and harvest regime: Positive (orange inverted triangles), Random (circles), and Negative harvest (blue triangles).     \|  \| \| --- \| \| Figure S4. Trends for mean length in mature males over time in harvest phase 1 (left panel), recovery phase (central panel), and harvest phase 2 (right panel). Change rate in haldanes within harvest phase 1 and harvest phase 2 shown in legends. Symbols refers to Positive (orange inverted triangles and dashed lines), Random (yellow circles and solid lines), and Negative harvest (blue triangles and dotted lines) \| |

| Table S6a*.* Results from the probability of being mature model with binomial distribution in males | | | | |
| --- | --- | --- | --- | --- |
| Predictors | Odds Ratios | CI | *z* value | *P* value |
| (Intercept): *Random, Harvest phase 1, Mean length* | 2.48 | 1.53–4.04 | 3.66 | **0.0003** |
| Mean length +1 SD | 15.34 | 12.7–18.52 | 28.33 | **<0.0001** |
| Negative | 3.35 | 1.97–5.68 | 4.48 | **<0.0001** |
| Positive | 2.64 | 1.25–5.56 | 2.55 | **0.011** |
| Recovery phase | 7.05 | 4.51–11.01 | 8.57 | **<0.0001** |
| Harvest phase 2 | 89.69 | 51.96–154.83 | 16.14 | **<0.0001** |
| Weeks in phase | 1.01 | 1.01–1.02 | 8.22 | **<0.0001** |
| Mean length +1 SD * Negative | 0.93 | 0.72–1.19 | -0.59 | 0.558 |
| Mean length +1 SD * Positive | 0.78 | 0.61–0.98 | -2.13 | **0.034** |
| Mean length +1 SD * Recovery phase | 1.21 | 0.86–1.71 | 1.09 | 0.277 |
| Mean length +1 SD * Harvest phase 2 | 2.57 | 1.79–3.71 | 5.08 | **<0.0001** |
| Negative * Recovery phase | 0.08 | 0.04–0.14 | -8.26 | **<0.0001** |
| Positive * Recovery phase | 0.23 | 0.13–0.42 | -4.91 | **<0.0001** |
| Negative * Harvest phase 2 | 0.39 | 0.18–0.87 | -2.30 | **0.022** |
| Positive * Harvest phase 2 | 0.54 | 0.27–1.09 | -1.72 | 0.085 |
| Negative * Weeks in phase | 0.99 | 0.99–1 | -3.73 | **0.000** |
| Positive * Weeks in phase | 0.99 | 0.99–1 | -4.19 | **<0.0001** |
| Recovery phase * Weeks in phase | 0.99 | 0.99–1 | -4.24 | **<0.0001** |
| Harvest phase 2 * Weeks in phase | 0.99 | 0.98–0.99 | -7.17 | **<0.0001** |
| Negative * Recovery phase *Mean length +1 SD | 0.63 | 0.41–0.98 | -2.04 | **0.041** |
| Positive * Recovery phase *Mean length +1 SD | 1.08 | 0.7–1.66 | 0.33 | 0.742 |
| Negative * Harvest phase 2 * Mean length +1 SD | 1.17 | 0.69–1.99 | 0.59 | 0.554 |
| Positive * Harvest phase 2 * Mean length +1 SD | 1.10 | 0.71–1.71 | 0.41 | 0.682 |
| Negative * Recovery phase * Weeks in phase | 1.01 | 1.01–1.02 | 3.93 | **0.000** |
| Positive * Recovery phase * Weeks in phase | 1.01 | 1.01–1.02 | 4.22 | **<0.0001** |
| Negative * Harvest phase 2 * Weeks in phase | 1.00 | 0.99–1 | -1.46 | 0.145 |
| Positive* Harvest phase 2 * Weeks in phase | 1.02 | 1.02–1.03 | 8.07 | **<0.0001** |

| Table S6b. Harvest regime pairwise comparison of probability of being mature in males at the start and the end of each experimental phase: harvest phase 1, recovery phase and harvest phase 2. Degrees of freedom = 43013. | | | | |
| --- | --- | --- | --- | --- |
| Contrasts | Odds Ratio | SE | *t* ratio | *P* value |
| Start of Harvest phase 1: | | | | |
| Random / Negative | 0.30 | 0.08 | -4.48 | **<0.0001** |
| Random / Positive | 0.38 | 0.14 | -2.55 | **0.011** |
| Negative /Positive | 1.27 | 0.48 | 0.63 | 0.530 |
| Start of Recovery phase: | | | | |
| Random / Negative | 3.81 | 1.11 | 4.60 | **<0.0001** |
| Random / Positive | 1.63 | 0.68 | 1.16 | 0.244 |
| Negative /Positive | 0.43 | 0.17 | -2.15 | **0.032** |
| Start of Harvest phase 2: | | | | |
| Random / Negative | 0.77 | 0.32 | -0.63 | 0.528 |
| Random / Positive | 0.70 | 0.32 | -0.78 | 0.436 |
| Negative /Positive | 0.91 | 0.43 | -0.20 | 0.846 |
| End of Harvest phase 1: | | | | |
| Random / Negative | 1.03 | 0.31 | 0.10 | 0.922 |
| Random / Positive | 1.31 | 0.52 | 0.70 | 0.486 |
| Negative /Positive | 1.28 | 0.51 | 0.62 | 0.538 |
| End of Recovery phase: | | | | |
| Random / Negative | 1.93 | 0.69 | 1.85 | 0.065 |
| Random / Positive | 0.87 | 0.40 | -0.30 | 0.763 |
| Negative /Positive | 0.45 | 0.20 | -1.83 | 0.068 |
| End of Harvest phase 2: | | | | |
| Random / Negative | 5.25 | 1.93 | 4.51 | **<0.0001** |
| Random / Positive | 0.08 | 0.04 | -5.43 | **<0.0001** |
| Negative /Positive | 0.01 | 0.01 | -9.40 | **<0.0001** |
| Difference between start and end of experiment (start/end) | | | | |
| In Random harvest | 0.019 | 0.005 | -14.46 | **<0.0001** |
| In Negative harvest | 0.338 | 0.075 | -4.86 | **<0.0001** |
| In Positive harvest | 0.004 | 0.001 | -21.93 | **<0.0001** |
| Different between start and end of harvest phase 2 | | |  |  |
| In Random harvest | 1.72 | 0.42 | 2.22 | **0.0267** |
| In Negative harvest | 0.55 | 0.13 | -2.57 | **0.0101** |
| In Positive harvest | 0.19 | 0.04 | -8.76 | **<0.0001** |
| Difference between end of phases |  |  |  |  |
| In Random harvest |  |  |  |  |
| Harvest1/ Recovery | 0.51 | 0.12 | -2.80 | **0.005** |
| Harvest1/ Harvest2 | 0.12 | 0.03 | -7.51 | **<0.0001** |
| Recovery/ Harvest2 | 0.24 | 0.07 | -4.65 | **<0.0001** |
| In Negative harvest |  |  |  |  |
| Harvest1/ Recovery | 1.17 | 0.24 | 0.76 | 0.447 |
| Harvest1/ Harvest2 | 0.64 | 0.16 | -1.82 | 0.069 |
| Recovery/ Harvest2 | 0.55 | 0.13 | -2.57 | **0.010** |
| In Positive harvest |  |  |  |  |
| Harvest1/ Recovery | 0.41 | 0.08 | -4.41 | **<0.0001** |
| Harvest1/ Harvest2 | 0.01 | 0.00 | -19.11 | **<0.0001** |
| Recovery/ Harvest2 | 0.02 | 0.01 | -14.28 | **<0.0001** |

| Table S6c. Elasticity estimates for a change in L50 in males and females relative to a change in biomass for three transitions: 1) from start of experiment (start) to end of harvest phase 1 (H1), 2) from end of recovery phase to end of harvest phase 2 (H2), and 3) from start of experiment to end of experiment (H2), for the three harvest regimes. | | | | |
| --- | --- | --- | --- | --- |
|  | Transition | Negative | Random | Positive |
| L50 Males | Start – H1 | 0.06 | 0.306 | 0.089 |
|  | Recovery – H2 | 0.224 | -0.104 | -19.844 |
|  | Start – H2 | 0.018 | 0.438 | 0.619 |
| L50 Females | Start – H1 | 0.008 | 0.55 | 0.211 |
|  | Recovery – H2 | NA | NA | NA |
|  | Start – H2 | 0.213 | 0.796 | 1.064 |

| Table S7a. Results from the probability of being mature model with binomial distribution in females | | | | |
| --- | --- | --- | --- | --- |
| Predictors | Odds Ratios | CI | z value | P value |
| (Intercept):  Random, Harvest 1 (week 48), Mean length | 2.96 | 1.42 – 6.19 | 2.89 | **0.004** |
| Mean length +1 SD | 28.2 | 16.57 – 48.01 | 12.3 | **<0.001** |
| Negative | 1.29 | 0.55 – 3.04 | 0.58 | 0.56 |
| Positive | 3.23 | 1.18 – 8.88 | 2.28 | **0.023** |
| Harvest 1 (week 100) | 4.26 | 1.66 – 10.92 | 3.02 | **0.003** |
| Harvest 1 (week 158) | 7.82 | 2.73 – 22.36 | 3.83 | **<0.001** |
| Harvest 2 (week 104) | 19.3 | 7.61 – 48.98 | 6.23 | **<0.001** |
| Mean length +1 SD * Negative | 0.43 | 0.23 – 0.81 | -2.63 | **0.009** |
| Mean length +1 SD * Positive | 1.71 | 0.76 – 3.88 | 1.29 | 0.196 |
| Negative * Harvest 1 (week 100) | 0.22 | 0.07 – 0.70 | -2.56 | **0.01** |
| Positive * Harvest 1 (week 100) | 0.63 | 0.18 – 2.17 | -0.73 | 0.467 |
| Negative * Harvest 1 (week 158) | 0.13 | 0.03 – 0.60 | -2.64 | **0.008** |
| Positive * Harvest 1 (week 158) | 0.42 | 0.11 – 1.67 | -1.23 | 0.219 |
| Negative * Harvest 2 (week 104) | 0.12 | 0.04 – 0.35 | -3.8 | **<0.001** |
| Positive * Harvest 2 (week 104) | 4.28 | 1.16 – 15.81 | 2.18 | **0.029** |

| Table S7b. Harvest regime pairwise comparison of probability of being mature in females at the 4 events assessed, three during harvest phase 1, and 1 during harvest phase 2. Degrees of freedom = 2583. | | | | |  |
| --- | --- | --- | --- | --- | --- |
| Contrasts | Odds Ratio | SE | *t* ratio | *P* value | |
| Harvest 1 (week 48) | | | | |  |
| Random / Negative | 0.23 | 0.12 | -2.76 | **0.006** | |
| Random / Positive | 0.67 | 0.41 | -0.66 | 0.509 | |
| Negative / Positive | 2.90 | 1.36 | 2.28 | **0.023** | |
| Harvest 1 (week 100) | | | | |  |
| Random / Negative | 1.02 | 0.46 | 0.05 | 0.958 | |
| Random / Positive | 1.06 | 0.46 | 0.13 | 0.900 | |
| Negative / Positive | 1.03 | 0.44 | 0.07 | 0.941 | |
| Harvest 1 (week 158) | | | | |  |
| Random / Negative | 1.73 | 1.12 | 0.85 | 0.397 | |
| Random / Positive | 1.59 | 0.86 | 0.86 | 0.391 | |
| Negative / Positive | 0.92 | 0.59 | -0.13 | 0.898 | |
| Harvest 2 (week 104) | | | | |  |
| Random / Negative | 1.98 | 0.68 | 1.99 | **0.047** | |
| Random / Positive | 0.16 | 0.05 | -5.92 | **<0.0001** | |
| Negative / Positive | 0.08 | 0.03 | -7.63 | **<0.0001** | |

### *4.2.2.* Fecundity

We assessed fecundity in females ranging the whole length distribution (min = 6 mm, max = 37 mm, mean ± SD = 15.5 ± 6.5 mm). The shortest female found carrying embryos was 13 mm, and thus the mean length of females above that 13 mm threshold was 19.56 ± 4.9 mm. Finally, the average length of a female with embryos was 22.1 ± 4.5 mm. Female fecundity (above 13 mm), both estimated as number of embryos and probability of having embryos, increased with increasing body length (Table S8). A female of average length above the 13 mm threshold exposed to Positive harvest would have on average 1.95 embryos and thus higher fecundity than a female exposed to Negative harvest, which would have 1.52 embryos. Such female (mean length above 13 mm) would have almost 2 embryos more during harvest phase 1 relative to harvest phase 2 (Table S8).

| Table S8. Results from fecundity (embryo count) model with negative binomial and zero truncated distribution. Length is standardised by 16 mm. to Note the zero-truncated model shows probability of having zero embryos. | | | | | |
| --- | --- | --- | --- | --- | --- |
| Predictors | Estimate | Log-Estimate | CI | *z* value | *P* value |
| Count Model | | | | | |
| (Intercept):  *Random, Harvest phase 1, Length=16* | 1.33 | 0.28 | 0.14 – 0.43 | 3.93 | **0.000** |
| log(Length) | 25.30 | 3.23 | 2.96 – 3.50 | 23.49 | **<0.0001** |
| Negative | 0.81 | -0.21 | -0.31 – -0.11 | -4.15 | **<0.0001** |
| Positive | 1.04 | 0.04 | -0.06 – 0.13 | 0.78 | 0.436 |
| Harvest phase 2 | 0.54 | -0.61 | -0.77 – -0.45 | -7.55 | **<0.0001** |
| Zero-Inflated Model | | | | | |
| (Intercept):  *Random, Harvest phase 1, Length=16* | 6.15 | 1.82 | 1.412 – 2.22 | 8.94 | **<0.0001** |
| log(Length) | 0.000009 | -11.61 | -12.76 – -10.45 | -19.65 | **<0.0001** |
| Negative | 1.57 | 0.45 | -0.035 – 0.93 | 1.82 | 0.069 |
| Positive | 0.75 | -0.28 | -0.75 – 0.19 | -1.18 | 0.238 |
| Harvest phase 2 | 0.20 | -1.60 | -2.17 – -1.03 | -5.51 | **<0.0001** |
| Negative * Harvest phase 2 | 2.02 | 0.70 | -0.11 – 1.52 | 1.70 | 0.090 |
| Positive * Harvest phase 2 | 0.21 | -1.57 | -2.34 – -0.80 | -3.98 | **0.000** |
| Dispersion model |  |  |  |  |  |
| (Intercept): *Length=16* | 492.26 | 6.20 | 3.38 – 9.02 | 4.31 | **<0.0001** |
| log(Length) | 0.002 | -6.19 | -10.67 – -1.70 | -2.70 | **0.007** |

### 4.2.3. Cohort somatic growth

Fish exposed to Positive harvest grew faster during the early ages than those exposed to Random and Negative harvest, growing the latter the slowest, (Table S9, Figure S5). These results agreed with those from the individual growth model from the genetic assessment (Section 4.3.3*.*). After age 6 (i.e., 36 weeks old), female growth in Positive harvest decelerates. Females grew faster than males in all cases (Table S9, Figure S5). Growth here refers to an increase in length per increase in age by 1 SD. Overall, fish had shorter average length (at average age) under high biomass compared to low biomass, except fish exposed to Random harvest and males exposed to Positive harvest where the opposite is true, and there is no difference in growth, respectively (Figure S4; Random female: Estimated difference = -1.67 mm, SE = 0.11, df = 15628, *t* ratio = -14.78; P value < 0.0001; Positive male: Estimated difference = -0.19 mm, SE = 0.12, df = 15628, *t* ratio = -1.53; P value = 0.126). Therefore, food was not limited in a way that hinder growth even at high biomass. The range of growth rate here (0.051 mm/day and 0.043 mm/day at low biomass for Positive and Negative harvest, respectively) were within the range of earlier published adult growth rates where guppies were exposed to intraspecific competition 0.009-0.14 mm/day (Potter *et al.* 2019), 0.002-0.026 mm/day (Reznick & Bryant 2007), 0.014–0.085 mm/day (Reznick *et al.* 2012), despite differences in the growth model used. At age 1 when all marked fish were immature, Random harvested fish were longer at high biomass, Negative harvested fish were shorter, and there was no difference in growth between high and low biomass in Positive harvested fish. Therefore, juveniles exposed to negatively size-dependent harvest grew slower under high relative low biomass. The opposite seemed to have occurred in medaka in an earlier size-selection experiment with semelparous life history (Evangelista *et al.* 2021).

| Table S9. Results from the cohort length model with Gaussian distribution. Estimates and confidence intervals (CI) in mm. Age and biomass are standardized to zero mean and unity standard deviation. Mean and SD for age 4.3 ± 3.2 6-weeks cycle (i.e. 27 weeks old), and biomass 78.6 ± 22.1 grams. | | | | |
| --- | --- | --- | --- | --- |
| Predictors | Estimates | CI | Statistic | p |
| (Intercept):  *Mean age, Mean biomass, Female, Random* | 23.07 | 22.37 – 23.77 | 64.64 | **<0.0001** |
| Age | 7.27 | 7.13 – 7.41 | 102.24 | **<0.0001** |
| Biomass | 1.42 | 1.24 – 1.61 | 14.77 | **<0.0001** |
| Negative | -1.77 | -2.75 – -0.78 | -3.51 | **0.0004** |
| Positive | -0.11 | -1.11 – 0.88 | -0.22 | 0.8253 |
| Male | -5.08 | -5.26 – -4.91 | -56.44 | **<0.0001** |
| Age^2^ | -1.91 | -2.01 – -1.82 | -40.04 | **<0.0001** |
| Age × Biomass | 0.49 | 0.29 – 0.70 | 4.71 | **<0.0001** |
| Age × Negative | -1.59 | -1.76 – -1.41 | -17.77 | **<0.0001** |
| Age × Positive | -0.63 | -0.87 – -0.39 | -5.17 | **<0.0001** |
| Biomass × Negative | -1.95 | -2.15 – -1.75 | -18.89 | **<0.0001** |
| Biomass × Positive | -2.13 | -2.41 – -1.84 | -14.58 | **<0.0001** |
| Age × Male | -6.01 | -6.25 – -5.77 | -49.65 | **<0.0001** |
| Biomass × Male | -1.06 | -1.32 – -0.80 | -7.99 | **<0.0001** |
| Negative × Male | 1.51 | 1.27 – 1.74 | 12.65 | **<0.0001** |
| Positive × Male | -0.25 | -0.56 – 0.05 | -1.62 | 0.1048 |
| Biomass × Age^2^ | -0.54 | -0.68 – -0.40 | -7.7 | **<0.0001** |
| Negative × Age^2^ | 0.54 | 0.44 – 0.65 | 10.41 | **<0.0001** |
| Positive × Age^2^ | -1.28 | -1.54 – -1.03 | -9.75 | **<0.0001** |
| Male × Age^2^ | 1.56 | 1.42 – 1.70 | 22.05 | **<0.0001** |
| Age × Biomass × Negative | -0.78 | -1.00 – -0.56 | -6.92 | **<0.0001** |
| Age × Biomass × Positive | -1.02 | -1.35 – -0.69 | -6.04 | **<0.0001** |
| Age × Biomass × Male | -1.02 | -1.35 – -0.68 | -5.9 | **<0.0001** |
| Age × Negative × Male | 1.72 | 1.40 – 2.03 | 10.76 | **<0.0001** |
| Age × Positive × Male | 0.73 | 0.33 – 1.12 | 3.56 | **0.0004** |
| Biomass × Negative x Male | 1.32 | 1.04 – 1.61 | 9.13 | **<0.0001** |
| Biomass × Positive x Male | 1.92 | 1.53 – 2.32 | 9.59 | **<0.0001** |
| Biomass × Negative × Age^2^ | 0.13 | -0.01 – 0.28 | 1.83 | 0.0667 |
| Biomass × Positive × Age^2^ | 1.04 | 0.68 – 1.40 | 5.67 | **<0.0001** |
| Biomass × Male × Age^2^ | 0.53 | 0.33 – 0.72 | 5.3 | **<0.0001** |
| Negative × Male × Age^2^ | -0.52 | -0.69 – -0.36 | -6.3 | **<0.0001** |
| Positive × Male × Age^2^ | 1.23 | 0.84 – 1.61 | 6.21 | **<0.0001** |
| Age × Biomass × Negative x Male | 1.28 | 0.90 – 1.65 | 6.67 | **<0.0001** |
| Age × Biomass × Positive x Male | 1.84 | 1.32 – 2.35 | 6.97 | **<0.0001** |
| Biomass × Negative x Male × Age^2^ | -0.17 | -0.39 – 0.04 | -1.59 | 0.1112 |
| Biomass × Positive x Male × Age^2^ | -0.89 | -1.38 – -0.40 | -3.55 | **0.0004** |

Figure S5. Cohort growth curves form mark-recapture fish in phenotypic assessment during harvest phase 1 for females (top panels) and males (bottom panels) and under high biomass (left panels) and low biomass (right panels).

## 4.3. Harvest-induced phenotypic [and demographic] changes are partially heritable and thus partially genetic

### 4.3.1. Length at maturation

| Table S10a*.* Harvest regime pairwise comparison of length at 63 days old (mean age at maturation) in males at the 6 genotypic assessments. Degrees of freedom = 5417 | | | | |
| --- | --- | --- | --- | --- |
| Contrast | Estimate | SE | *t* ratio | *P* value |
| Initial phase |  |  |  |  |
| Random - Negative | -0.32 | 0.41 | -0.79 | 0.429 |
| Random - Positive | -0.11 | 0.40 | -0.28 | 0.780 |
| Negative - Positive | 0.21 | 0.41 | 0.51 | 0.608 |
| Harvest phase 1 |  |  |  |  |
| Random - Negative | -0.15 | 0.11 | -1.34 | 0.180 |
| Random - Positive | 0.01 | 0.11 | 0.09 | 0.931 |
| Negative - Positive | 0.16 | 0.11 | 1.42 | 0.155 |
| Recovery phase |  |  |  |  |
| Random - Negative | -0.24 | 0.17 | -1.39 | 0.165 |
| Random - Positive | -0.19 | 0.18 | -1.10 | 0.270 |
| Negative - Positive | 0.05 | 0.18 | 0.26 | 0.796 |
| Harvest pase 2 |  |  |  |  |
| Random - Negative | -0.26 | 0.15 | -1.72 | 0.085 |
| Random - Positive | 0.18 | 0.14 | 1.30 | 0.192 |
| Negative - Positive | 0.44 | 0.14 | 3.12 | **0.002** |
| Random |  |  |  |  |
| Start - Harvest1 | 5.42 | 0.29 | **18.41** | **<0.0001** |
| Start - Recovery | 5.54 | 0.31 | **17.92** | **<0.0001** |
| Start - Harvest2 | 4.92 | 0.30 | **16.26** | **<0.0001** |
| Harvest1 - Recovery | 0.13 | 0.15 | 0.87 | 0.386 |
| Harvest1 - Harvest2 | -0.50 | 0.13 | -3.81 | **0.0001** |
| Recovery - Harvest2 | -0.62 | 0.16 | -3.85 | **0.0001** |
| Negative |  |  |  |  |
| Start - Harvest1 | 5.59 | 0.31 | 18.25 | **<0.0001** |
| Start - Recovery | 5.62 | 0.32 | 17.59 | **<0.0001** |
| Start - Harvest2 | 4.99 | 0.31 | 15.91 | **<0.0001** |
| Harvest1 - Recovery | 0.03 | 0.15 | 0.24 | 0.812 |
| Harvest1 - Harvest2 | -0.60 | 0.13 | -4.56 | **<0.0001** |
| Recovery - Harvest2 | -0.64 | 0.16 | -3.96 | **0.0001** |
| Positive |  |  |  |  |
| Start - Harvest1 | 5.54 | 0.30 | 18.67 | **<0.0001** |
| Start - Recovery | 5.46 | 0.31 | 17.42 | **<0.0001** |
| Start - Harvest2 | 5.22 | 0.30 | 17.30 | **<0.0001** |
| Harvest1 - Recovery | -0.08 | 0.15 | -0.53 | 0.598 |
| Harvest1 - Harvest2 | -0.32 | 0.12 | -2.63 | **0.009** |
| Recovery - Harvest2 | -0.24 | 0.16 | -1.53 | 0.126 |

| Table S10b. Harvest regime pairwise comparison of length at 141 days old (mean age at maturation) in females at the 6 genotypic assessments. Degrees of freedom =11426 | | | | |
| --- | --- | --- | --- | --- |
| Contrast | Estimate | SE | *t* ratio | *P* value |
| Start |  |  |  |  |
| Random - Negative | -0.04 | 0.23 | -0.16 | 0.875 |
| Random - Positive | 0.03 | 0.23 | 0.12 | 0.904 |
| Negative - Positive | 0.06 | 0.24 | 0.27 | 0.789 |
| Harvest1 |  |  |  |  |
| Random - Negative | -0.19 | 0.12 | -1.58 | 0.114 |
| Random - Positive | 0.14 | 0.12 | 1.12 | 0.264 |
| Negative - Positive | 0.33 | 0.12 | 2.71 | **0.007** |
| Recovery |  |  |  |  |
| Random - Negative | -0.85 | 0.22 | -3.82 | **0.0001** |
| Random - Positive | -1.28 | 0.23 | -5.67 | **<0.0001** |
| Negative - Positive | -0.43 | 0.23 | -1.86 | 0.063 |
| Harvest2 |  |  |  |  |
| Random - Negative | -0.18 | 0.16 | -1.15 | 0.249 |
| Random - Positive | 0.26 | 0.15 | 1.75 | 0.081 |
| Negative - Positive | 0.44 | 0.15 | 2.90 | **0.004** |
| Random |  |  |  |  |
| Start - Harvest1 | 2.46 | 0.18 | 13.71 | **<0.0001** |
| Start - Recovery | 2.63 | 0.22 | 11.91 | **<0.0001** |
| Start - Harvest2 | 4.64 | 0.19 | 24.26 | **<0.0001** |
| Harvest1 - Recovery | 0.18 | 0.18 | 0.98 | 0.327 |
| Harvest1 - Harvest2 | 2.18 | 0.14 | 15.60 | **<0.0001** |
| Recovery - Harvest2 | 2.01 | 0.19 | 10.53 | **<0.0001** |
| Negative |  |  |  |  |
| Start - Harvest1 | 2.30 | 0.19 | 12.08 | **<0.0001** |
| Start - Recovery | 1.81 | 0.23 | 7.74 | **<0.0001** |
| Start - Harvest2 | 4.49 | 0.21 | 21.90 | **<0.0001** |
| Harvest1 - Recovery | -0.49 | 0.18 | -2.69 | **0.007** |
| Harvest1 - Harvest2 | 2.19 | 0.14 | 15.45 | **<0.0001** |
| Recovery - Harvest2 | 2.68 | 0.20 | 13.61 | **<0.0001** |
| Positive |  |  |  |  |
| Start - Harvest1 | 2.57 | 0.19 | 13.65 | **<0.0001** |
| Start - Recovery | 1.33 | 0.23 | 5.68 | **<0.0001** |
| Start - Harvest2 | 4.87 | 0.20 | 24.92 | **<0.0001** |
| Harvest1 - Recovery | -1.24 | 0.19 | -6.70 | **<0.0001** |
| Harvest1 - Harvest2 | 2.31 | 0.14 | 17.05 | **<0.0001** |
| Recovery - Harvest2 | 3.55 | 0.19 | 18.38 | **<0.0001** |

### 4.3.2. Probability of becoming mature

There were no differences among harvesting regimes in length and age at which 50% of males became mature (Lp50) during the initial phase before any size-selective harvest started (Figure S6; Table S11a). However, in harvest phase 1, males exposed to Positive harvest had twice the odds of maturing relative to all other harvest regimes, being the difference larger between males exposed to Positive and Random harvest (Figure S6; Table S11a). This difference between males exposed to Positive and Random harvest was shifted in the recovery phase, there having males exposed to Random harvest almost 4 times the odds of maturing than Positive (Figure S6; Table S11b). Males exposed to random harvest also seemed to have 3 times more odds of maturing than males exposed to Negative harvest, but this difference was not significant (P-value = 0.0510; Table S11b). In addition, during the recovery phase, males exposed to Positive and Negative harvest did no longer differ in probability of becoming mature. Finally, in harvest phase 2 there were no differences in probability of becoming mature among harvest regimes.

For females, there were no differences among harvesting regimes in length and age at which 50% of them became mature (Lp50) during the initial phase and the final harvest phase 2, as in the case of males. During harvest phase 1 and recovery phase, females exposed to Positive harvest had twice and 3 times the odds of becoming mature than females exposed to Negative harvest, respectively (Table S12b). Females exposed to Positive harvest did not differ from females exposed to Random harvest in none of the experiment phases. These results occurred when considering females of average length (19.8 ± 2.04 mm, SL) and age (140 ± 45 days old) at maturation (Figure S7; Table S12a). The lack of stronger differences could be due to the much larger variance in length but mainly in age at maturation in females (i.e., time of first brood birth). There is less certainty in female time of first parturition, as some females might give birth to only one offspring and cannibalise on it before we can note it.

Figure S6. Length and age at which the probability of becoming mature is 50% (Lp50), 25% (Lp25) and 75%

(Lp75) for males (Lp50=symbols, Lp25=dashed lines, Lp75=dotted lines), for each experimental phase: initial phase (before size-selection) in the first pale-shaded panel, harvest phase 1, recovery phase dark-shaded panel, and harvest phase 2 in the right panel; and harvest regime: Positive (orange inverted triangles), Random (yellow circles), and Negative harvest (blue triangles).

| Table S11a*.* Results from the probability of becoming mature model with binomial distribution in males | | | | |
| --- | --- | --- | --- | --- |
| Predictors | Odds Ratios | CI | z value | P value |
| (Intercept):  *Random, Initial, Mean length and age* | 0.24 | 0.11 – 0.50 | -3.75 | **<0.001** |
| Mean length +1 SD | 3.62 | 2.11 – 6.21 | 4.67 | **<0.001** |
| Negative | 1.2 | 0.43 – 3.40 | 0.35 | 0.726 |
| Positive | 1 | 0.35 – 2.86 | 0 | 0.999 |
| Harvest 1 | 2.01 | 0.89 – 4.54 | 1.68 | 0.092 |
| Recovery | 31.19 | 9.25 – 105.21 | 5.55 | **<0.001** |
| Harvest 2 | 4.84 | 1.88 – 12.46 | 3.26 | **0.001** |
| Mean age +1 SD | 1.26 | 1.14 – 1.40 | 4.37 | **<0.001** |
| Mean length +1 SD * Negative | 0.96 | 0.46 – 2.02 | -0.11 | 0.916 |
| Mean length +1 SD * Positive | 1.1 | 0.51 – 2.39 | 0.25 | 0.803 |
| Mean length +1 SD * Harvest 1 | 0.45 | 0.26 – 0.79 | -2.81 | **0.005** |
| Mean length +1 SD * Recovery | 2.45 | 0.97 – 6.16 | 1.9 | 0.057 |
| Mean length +1 SD * Harvest 2 | 0.91 | 0.47 – 1.75 | -0.29 | 0.775 |
| Negative * Harvest 1 | 0.94 | 0.31 – 2.87 | -0.11 | 0.912 |
| Positive * Harvest 1 | 2.36 | 0.76 – 7.36 | 1.48 | 0.139 |
| Negative * Recovery | 0.27 | 0.06 – 1.25 | -1.68 | 0.093 |
| Positive * Recovery | 0.26 | 0.05 – 1.23 | -1.7 | 0.09 |
| Negative * Harvest 2 | 0.6 | 0.17 – 2.17 | -0.77 | 0.439 |
| Positive * Harvest 2 | 1.02 | 0.28 – 3.75 | 0.03 | 0.975 |
| Mean length +1 SD * Negative * Harvest 1 | 1.24 | 0.57 – 2.69 | 0.55 | 0.579 |
| Mean length +1 SD * Positive * Harvest 1 | 3.03 | 1.29 – 7.12 | 2.54 | **0.011** |
| Mean length +1 SD * Negative * Recovery | 0.92 | 0.26 – 3.23 | -0.13 | 0.898 |
| Mean length +1 SD * Positive * Recovery | 0.47 | 0.14 – 1.57 | -1.22 | 0.221 |
| Mean length +1 SD * Negative * Harvest 2 | 0.91 | 0.37 – 2.25 | -0.2 | 0.843 |
| Mean length +1 SD * Positive * Harvest 2 | 0.85 | 0.34 – 2.12 | -0.36 | 0.721 |

| Table S11b. Harvest regime pairwise comparison of probability of becoming mature in males (genetic assessment from 6 common garden experiments) during four phases: initial phase, harvest phase 1, recover, and harvest phase 2 for males of average age and length at maturation. Degrees of freedom = 4134. | | | | |
| --- | --- | --- | --- | --- |
| Contrast | Odds Ratio | SE | t ratio | P value |
| Initial phase | | | | |
| Random/Negative | 0.83 | 0.44 | -0.35 | 0.727 |
| Random/Positive | 1.00 | 0.54 | 0.00 | 0.999 |
| Negative/Positive | 1.20 | 0.64 | 0.35 | 0.728 |
| Harvest phase 1 | | | | |
| Random/Negative | 0.89 | 0.19 | -0.58 | 0.563 |
| Random/Positive | 0.42 | 0.09 | -3.86 | **0.0001** |
| Negative/Positive | 0.48 | 0.11 | -3.28 | **0.001** |
| Recovery phase | | | | |
| Random/Negative | 3.12 | 1.82 | 1.95 | *0.051* |
| Random/Positive | 3.86 | 2.28 | 2.29 | **0.022** |
| Negative/Positive | 1.24 | 0.59 | 0.44 | 0.659 |
| Harvest phase 2 | | | | |
| Random/Negative | 1.38 | 0.53 | 0.84 | 0.404 |
| Random/Positive | 0.98 | 0.38 | -0.06 | 0.956 |
| Negative/Positive | 0.71 | 0.26 | -0.92 | 0.358 |
| Random |  |  |  |  |
| Start / Harvest1 | 0.50 | 0.21 | -1.69 | 0.092 |
| Start / Recovery | 0.03 | 0.02 | -5.55 | **<0.0001** |
| Start / Harvest2 | 0.21 | 0.10 | -3.26 | **0.001** |
| Harvest1 / Recovery | 0.06 | 0.03 | -5.46 | **<0.0001** |
| Harvest1 / Harvest2 | 0.42 | 0.13 | -2.73 | **0.006** |
| Recovery / Harvest2 | 6.45 | 3.60 | 3.34 | **0.001** |
| Negative |  |  |  |  |
| Start / Harvest1 | 0.53 | 0.22 | -1.56 | 0.118 |
| Start / Recovery | 0.12 | 0.06 | -4.21 | **<0.0001** |
| Start / Harvest2 | 0.34 | 0.16 | -2.35 | **0.019** |
| Harvest1 / Recovery | 0.23 | 0.08 | -4.06 | **0.0001** |
| Harvest1 / Harvest2 | 0.65 | 0.19 | -1.46 | 0.145 |
| Recovery / Harvest2 | 2.84 | 1.19 | 2.49 | **0.013** |
| Positive |  |  |  |  |
| Start / Harvest1 | 0.21 | 0.09 | -3.73 | **0.0002** |
| Start / Recovery | 0.12 | 0.06 | -4.03 | **0.0001** |
| Start / Harvest2 | 0.20 | 0.10 | -3.37 | **0.001** |
| Harvest1 / Recovery | 0.59 | 0.22 | -1.39 | 0.165 |
| Harvest1 / Harvest2 | 0.96 | 0.31 | -0.12 | 0.904 |
| Recovery / Harvest2 | 1.64 | 0.71 | 1.13 | 0.260 |

| Table S12a*.* Results from the probability of becoming mature model with binomial distribution in females | | | | |
| --- | --- | --- | --- | --- |
| Predictors | Odds Ratios | CI | Statistic | p |
| (Intercept):  Random, Initial, Mean length/age | 0.55 | 0.30 – 1.03 | -1.86 | 0.062 |
| Mean age +1 SD | 1.35 | 0.81 – 2.25 | 1.15 | 0.25 |
| Negative | 1.75 | 0.72 – 4.25 | 1.23 | 0.218 |
| Positive | 1.55 | 0.64 – 3.75 | 0.97 | 0.334 |
| Harvest 1 | 1.64 | 0.82 – 3.31 | 1.39 | 0.164 |
| Recovery | 4.06 | 1.66 – 9.93 | 3.07 | **0.002** |
| Harvest 2 | 1.48 | 0.65 – 3.39 | 0.93 | 0.351 |
| Mean length + 1 SD | 3.19 | 2.60 – 3.91 | 11.09 | **<0.001** |
| Mean age +1 SD * Negative | 1.34 | 0.68 – 2.68 | 0.84 | 0.399 |
| Mean age +1 SD * Positive | 1.05 | 0.53 – 2.06 | 0.14 | 0.891 |
| Mean age +1 SD * Harvest 1 | 1.24 | 0.71 – 2.16 | 0.77 | 0.443 |
| Mean age +1 SD * Recovery | 1 | 0.53 – 1.87 | -0.01 | 0.989 |
| Mean age +1 SD * Harvest 2 | 0.83 | 0.49 – 1.39 | -0.71 | 0.475 |
| Negative * Harvest 1 | 0.4 | 0.15 – 1.10 | -1.77 | 0.076 |
| Positive * Harvest 1 | 0.93 | 0.34 – 2.59 | -0.13 | 0.893 |
| Negative * Recovery | 0.47 | 0.13 – 1.67 | -1.17 | 0.243 |
| Positive * Recovery | 1.75 | 0.45 – 6.84 | 0.8 | 0.422 |
| Negative * Harvest 2 | 0.46 | 0.15 – 1.36 | -1.41 | 0.16 |
| Positive * Harvest 2 | 0.48 | 0.16 – 1.41 | -1.34 | 0.181 |
| Mean age +1 SD * Negative *Harvest 1 | 0.61 | 0.27 – 1.35 | -1.22 | 0.222 |
| Mean age +1 SD * Positive* Harvest 1 | 1.01 | 0.45 – 2.28 | 0.03 | 0.972 |
| Mean age +1 SD * Negative * Recovery | 0.92 | 0.35 – 2.43 | -0.17 | 0.867 |
| Mean age +1 SD * Positive * Recovery | 4.16 | 1.22 – 14.15 | 2.28 | **0.022** |
| Mean age +1 SD * Negative *Harvest 2 | 0.77 | 0.36 – 1.63 | -0.69 | 0.491 |
| Mean age +1 SD * Positive * Harvest 2 | 0.81 | 0.39 – 1.70 | -0.55 | 0.584 |

| Table S12b. Harvest regime pairwise comparison of probability of becoming mature in females (genetic assessment from 6 common garden experiments) during four phases: initial phase, harvest phase 1, recover, and harvest phase 2 for females of average age and length at maturation (comparison for all harvest phases). Degrees of freedom = 7717. | | | | | |
| --- | --- | --- | --- | --- | --- |
| Contrast | Odds Ratio | SE | *t* ratio | *P* value |  |
| Initial, Mean age | | | | | |
| Random/Negative | 0.57 | 0.26 | -1.23 | 0.218 |  |
| Random/Positive | 0.65 | 0.29 | -0.97 | 0.334 |  |
| Negative/Positive | 1.13 | 0.53 | 0.26 | 0.794 |  |
| Harvest 1, Mean age | | | | | |
| Random/Negative | 1.43 | 0.35 | 1.45 | 0.147 |  |
| Random/Positive | 0.69 | 0.18 | -1.40 | 0.161 |  |
| Negative/Positive | 0.49 | 0.13 | -2.80 | **0.005** |  |
| Recovery, Mean age | | | | | |
| Random/Negative | 1.22 | 0.56 | 0.43 | 0.671 |  |
| Random/Positive | 0.37 | 0.20 | -1.88 | 0.060 |  |
| Negative/Positive | 0.30 | 0.16 | -2.23 | **0.026** |  |
| Harvest 2, Mean age | | | | | |
| Random/Negative | 1.25 | 0.41 | 0.70 | 0.486 |  |
| Random/Positive | 1.35 | 0.42 | 0.95 | 0.341 |  |
| Negative/Positive | 1.08 | 0.35 | 0.23 | 0.820 |  |
| Random |  |  |  |  |  |
| Start / Harvest1 | 0.61 | 0.22 | -1.39 | 0.164 |  |
| Start / Recovery | 0.25 | 0.11 | -3.07 | **0.002** |  |
| Start / Harvest2 | 0.67 | 0.28 | -0.93 | 0.351 |  |
| Harvest1 / Recovery | 0.41 | 0.15 | -2.43 | **0.015** |  |
| Harvest1 / Harvest2 | 1.11 | 0.36 | 0.32 | 0.749 |  |
| Recovery / Harvest2 | 2.74 | 1.10 | 2.51 | **0.012** |  |
| Negative |  |  |  |  |  |
| Start / Harvest1 | 1.52 | 0.57 | 1.12 | 0.264 |  |
| Start / Recovery | 0.52 | 0.25 | -1.38 | 0.168 |  |
| Start / Harvest2 | 1.48 | 0.65 | 0.88 | 0.377 |  |
| Harvest1 / Recovery | 0.34 | 0.13 | -2.84 | **0.005** |  |
| Harvest1 / Harvest2 | 0.97 | 0.32 | -0.09 | 0.930 |  |
| Recovery / Harvest2 | 2.82 | 1.21 | 2.43 | **0.015** |  |
| Positive |  |  |  |  |  |
| Start / Harvest1 | 0.65 | 0.25 | -1.12 | 0.264 |  |
| Start / Recovery | 0.14 | 0.08 | -3.66 | **0.0003** |  |
| Start / Harvest2 | 1.41 | 0.62 | 0.77 | 0.442 |  |
| Harvest1 / Recovery | 0.22 | 0.10 | -3.33 | **0.001** |  |
| Harvest1 / Harvest2 | 2.16 | 0.75 | 2.22 | **0.027** |  |
| Recovery / Harvest2 | 9.99 | 5.13 | 4.48 | <.0001 |  |

Figure S7. Length and age at which the probability of becoming mature is 50% (Lp50), 25% (Lp25) and 75% (Lp75) for females (Lp50=symbols, Lp25=dashed lines, Lp75=dotted lines), for each experimental phase: initial phase (before size-selection) in the first pale-shaded panel, harvest phase 1, recovery phase dark-shaded panel, and harvest phase 2 in the right panel; and harvest regime: Positive (orange inverted triangles), Random (yellow circles), and Negative harvest (blue triangles).

### 4.3.3. Energy acquisition and allocation between growth and reproduction

Male growth curves differed very little among Harvest regimes, as only fish exposed to Negative harvest had lower coefficient in the energy acquisition rate, *c*, relative to fish exposed to random harvest (Figure S8; Table S13a). Lower *c* in Negative harvested individuals is represented by a slower (less steep) juvenile growth. Males’ growth curves differed among phases in the experiment, due to phase affecting both juvenile growth – through the coefficient in the energy acquisition rate, *c* – and adult growth – through the reproductive investment, *r*. The highest values of *c* were present in fish exposed to the Recovery phase, second highest values were in the Initial phase, followed by harvest phase 2, and finally harvest phase 1 with the lowest estimated values of *c*. Therefore, juvenile curves are steeper in the Recovery and Initial phases, relative to the harvest phases 1 and 2 (Figure S8). The highest investment in reproduction, *r*, occurred in fish in the Initial phase, followed by those in the Recovery phase, while the lowest investment was in fish during the harvest phases (which did not differ; Table S13a). The highest investment in reproduction together with youngest age at maturation during Initial and Recovery phases resulted in an earlier shift towards the slower adult growth, relative to both harvest phases (Figure S8). Overall fish exposed to Positive harvest seem to plateau at shorter lengths relative to the other Harvest regimes in harvest phases 1 and 2; while it is the fish exposed to Random harvest that plateau at shorter length in the Recovery phase. It should be noted that the model does not accurately estimate the plateau part of the curve in the Initial phase, as our data on adult fish is lacking in that phase compared to the other phases.

Female growth curves were more strongly affected (through changes in α, *c* and *r*) by harvest regimes relative to males (only affected through *c*), although harvest regime effects were different among phases in the experiment. Female juvenile growth was steeper – through higher values of coefficient in the energy acquisition rate, *c –* in fish exposed to Positive harvest relative to the other harvest regimes only during the harvest phase 1 and the recovery phase (Figure S9, Table S14a). There was no difference in *c* among harvest regimes in the remaining phases of the experiment. Females exposed to Negative harvest presented a higher investment in reproduction, *r*, relative to females exposed to Positive harvest in all phases (Table S14a). However, females exposed to Negative harvest had older age at maturation relative to females exposed to Positive harvest in all phases, except the initial one. The highest investment in reproduction and the oldest age at maturation occurred in harvest phase 2, while the lowest *r* and youngest age at maturation occurred in the Recovery phase (Table S14a). Overall, this resulted in 1) females during the initial phase having an equal juvenile growth in all harvest regimes, but the younger age at maturation and the higher investment in reproduction of Females exposed to Negative harvest led to slower adult growth, while females exposed to Positive and Random harvest did not differ in the Initial phase. 2) During the harvest phase 1, females exposed to Positive harvest had faster juvenile growth (i.e., higher values of *c*) and younger age at maturation, the latter resulting in an earlier shift towards adult growth relative to Negative harvest females. However, the lower investment in reproduction in females exposed to Positive harvest relative to Negative harvest resulted in overall faster adult growth rate in Positive harvest females. 3) During the recovery phase, the growth patterns are like those in harvest phase 1. 4) During harvest phase 2, females exposed to Positive harvest had the same juvenile growth as the other harvest regimes, thus the slower adult growth rate (and growth plateau at shorter length) in females exposed to Positive harvest is a result of the younger age at maturation – earlier shift towards adult growth – and a higher investment in reproduction, relative to other phases, despite being still smaller than in females exposed to Negative harvest.

Figure S8. Male biphasic growth curves for males in the different phases of the experiment (panels) for Negative (dashed black line), Random (solid black line), and Positive (dotted black line) size-selective harvest. Individual fish growth curves are shown in grey.

| Table S13a. Male biphasic growth models estimation of coefficient in the energy acquisition rate, *c*, and reproductive investment, *r,* given the fixed parameters in Table S2a. SE refers to standard error and CI to confidence intervals. Results from harvest regime and phase comparisons are in Table S13b. | | | | | | | |
| --- | --- | --- | --- | --- | --- | --- | --- |
|  |  | *c* | | | *r* | | |
| Phase | Treatment | Estimate | Lower CI | Upper CI | Estimate | Lower CI | Upper CI |
| Initial | Random | 0.52 | 0.50 | 0.53 | 0.054 | 0.050 | 0.058 |
|  | Negative | 0.52 | 0.51 | 0.54 |  |  |  |
|  | Positive | 0.52 | 0.50 | 0.53 |  |  |  |
| Harvest 1 | Random | 0.38 | 0.37 | 0.39 | 0.028 | 0.027 | 0.028 |
|  | Negative | 0.39 | 0.38 | 0.40 |  |  |  |
|  | Positive | 0.38 | 0.37 | 0.39 |  |  |  |
| Recovery | Random | 0.62 | 0.61 | 0.64 | 0.029 | 0.028 | 0.030 |
|  | Negative | 0.63 | 0.62 | 0.64 |  |  |  |
|  | Positive | 0.63 | 0.61 | 0.64 |  |  |  |
| Harvest 2 | Random | 0.40 | 0.39 | 0.41 | 0.028 | 0.027 | 0.029 |
|  | Negative | 0.41 | 0.40 | 0.42 |  |  |  |
|  | Positive | 0.41 | 0.40 | 0.42 |  |  |  |

| Table S13b. Comparison of male biphasic growth models parameters: coefficient in the energy acquisition rate, *c*, and reproductive investment, *r,* among phases in the experiment and Harvest regimes*.* SE refers to standard error. Parameters fixed in the biphasic model are given in Table S2a, and estimated values of *c* and *r* are given in Table S13a. | | | | | | | | | | | | | | |
| --- | --- | --- | --- | --- | --- | --- | --- | --- | --- | --- | --- | --- | --- | --- |
|  | *c* | | | | |  | |  | | |  |  |  |  |
| Contrast | Estimated difference | SE | *t* ratio | *P* value | | Estimated difference | | | SE | | | | *t* ratio | *P* value |
| Random-Negative | -0.007 | 0.003 | -2.45 | | **0.014** | |  | | |  | |  | |  |
| Random-Positive | -0.002 | 0.003 | -0.82 | | 0.413 | |  | | |  | |  | |  |
| Negative-Positive | 0.005 | 0.003 | 1.64 | | 0.102 | |  | | |  | |  | |  |
| Initial -Harvest1 | 0.136 | 0.008 | 17.33 | | **<0.0001** | | 0.0263 | | | 0.0022 | | 11.89 | | **<0.0001** |
| Initial -Recovery | -0.107 | 0.009 | -11.23 | | **<0.0001** | | 0.0246 | | | 0.0022 | | 10.98 | | **<0.0001** |
| Initial -Harvest2 | 0.112 | 0.009 | 13.00 | | **<0.0001** | | 0.0259 | | | 0.0022 | | 11.67 | | **<0.0001** |
| Harvest1-Recovery | -0.242 | 0.007 | -33.07 | | **<0.0001** | | -0.0017 | | | 0.0005 | | -3.38 | | **0.0007** |
| Harvest1-Harvest2 | -0.024 | 0.006 | -3.84 | | **0.0001** | | -0.0003 | | | 0.0004 | | -0.76 | | 0.447 |
| Recovery-Harvest2 | 0.219 | 0.008 | 26.79 | | **<0.0001** | | 0.0014 | | | 0.0006 | | 2.48 | | **0.013** |

Figure S9. Female biphasic growth curves for males in the different phases of the experiment (panels) for Negative (dashed black line), Random (solid black line), and Positive (dotted black line) size-selective harvest. Individual fish growth curves are shown in grey.

| Table S14a. Female biphasic growth models estimation of coefficient in the energy acquisition rate, *c*, and reproductive investment, *r,* given the fixed parameters in Table S2a. SE refers to standard error and CI to confidence intervals. Results from harvest regime and phase comparisons are in Table S14b. | | | | | | | |
| --- | --- | --- | --- | --- | --- | --- | --- |
|  |  | *c* | |  |  | *r* | |
| Phase | Treatment | Estimate | Lower CI | Upper CL | Estimate | Lower CI | Upper CL |
| Initial | Random | 11.37 | 10.86 | 11.88 | 0.0068 | 0.0062 | 0.0075 |
|  | Negative | 10.87 | 10.35 | 11.38 | 0.0074 | 0.0067 | 0.0081 |
|  | Positive | 11.47 | 10.96 | 11.99 | 0.0064 | 0.0057 | 0.0071 |
| Harvest 1 | Random | 9.33 | 9.03 | 9.63 | 0.0050 | 0.0046 | 0.0055 |
|  | Negative | 9.33 | 9.03 | 9.63 | 0.0056 | 0.0051 | 0.0061 |
|  | Positive | 9.96 | 9.66 | 10.26 | 0.0046 | 0.0042 | 0.0051 |
| Recovery | Random | 14.35 | 13.82 | 14.88 | 0.0039 | 0.0032 | 0.0045 |
|  | Negative | 14.65 | 14.12 | 15.18 | 0.0044 | 0.0038 | 0.0051 |
|  | Positive | 15.70 | 15.17 | 16.23 | 0.0035 | 0.0028 | 0.0041 |
| Harvest 2 | Random | 7.76 | 7.33 | 8.19 | 0.0088 | 0.0082 | 0.0094 |
|  | Negative | 7.56 | 7.11 | 8.01 | 0.0093 | 0.0087 | 0.0100 |
|  | Positive | 7.45 | 6.99 | 7.91 | 0.0084 | 0.0078 | 0.0090 |

| Table S14b. Comparison of female biphasic growth models parameters: coefficient in the energy acquisition rate, *c*, and reproductive investment, *r,* among phases in the experiment and Harvest regimes*.* SE refers to standard error. Parameters fixed in the biphasic model are given in Table S2a, and estimated values of *c* and *r* are given in Table S14a. | | | | | |
| --- | --- | --- | --- | --- | --- |
|  | *c* | | | | |
|  | Contrast | Estimate difference | SE | *t* ratio | *P* value |
| Initial | Random-Negative | 0.51 | 0.35 | 1.46 | 0.145 |
|  | Random-Positive | -0.10 | 0.35 | -0.29 | 0.770 |
|  | Negative-Positive | -0.61 | 0.35 | -1.73 | 0.083 |
| Harvest 1 | Random-Negative | 0.00 | 0.21 | -0.01 | 0.995 |
|  | Random-Positive | -0.63 | 0.21 | -3.03 | **0.002** |
|  | Negative-Positive | -0.63 | 0.21 | -3.04 | **0.002** |
| Recovery | Random-Negative | -0.30 | 0.36 | -0.84 | 0.403 |
|  | Random-Positive | -1.35 | 0.36 | -3.75 | **0.0002** |
|  | Negative-Positive | -1.05 | 0.36 | -2.93 | **0.003** |
| Harvest 2 | Random-Negative | 0.20 | 0.30 | 0.67 | 0.504 |
|  | Random-Positive | 0.31 | 0.31 | 1.00 | 0.315 |
|  | Negative-Positive | 0.11 | 0.32 | 0.34 | 0.738 |
|  | *r* | | | | |
|  | Contrast | Estimate  difference | SE | *t* ratio | *P* value |
|  | Random-Negative | -0.0006 | 0.0003 | -1.89 | 0.0586 |
|  | Random-Positive | 0.0004 | 0.0003 | 1.38 | 0.1683 |
|  | Negative-Positive | 0.0010 | 0.0003 | 3.27 | **0.0011** |
|  | Initial -Harvest 1 | 0.0018 | 0.0004 | 5.06 | **<0.0001** |
|  | Initial -Recovery | 0.0030 | 0.0004 | 6.89 | **<0.0001** |
|  | Initial - Harvest 2 | -0.0020 | 0.0004 | -4.75 | **<0.0001** |
|  | Harvest 1-Recovery | 0.0012 | 0.0003 | 3.39 | **0.0007** |
|  | Harvest 1-Harvest 2 | -0.0038 | 0.0003 | -11.93 | **<0.0001** |
|  | Recovery-Harvest 2 | -0.0049 | 0.0004 | -12.41 | **<0.0001** |

### 4.3.4. Fecundity: First-broods and lifetime fecundity

| Table S15. Results from fecundity a) offspring count of first two broods with Poisson distribution, where length is standardised by 16 mm, and b) lifetime offspring count with negative binomial distribution | | | | | |
| --- | --- | --- | --- | --- | --- |
| Predictors | Estimate | Log-estimate | CI | z value | P value |
| 1. Offspring count of first two broods | | | | | |
| (Intercept):  Random, Initial phase, Length=16 | 1.14 | 0.13 | -0.166 – 0.419 | 0.85 | 0.395 |
| log(Length) |  | 4.16 | 3.310 – 5.009 | 9.59 | **<0.0001** |
| Negative | 1.01 | 0.01 | -0.080 – 0.101 | 0.23 | 0.821 |
| Positive | 1.02 | 0.02 | -0.074 – 0.104 | 0.33 | 0.745 |
| Harvest phase 1 | 1.17 | 0.16 | -0.183 – 0.496 | 0.90 | 0.367 |
| Recovery phase | 1.42 | 0.35 | -0.031 – 0.732 | 1.80 | 0.072 |
| Harvest phase 2 | 1.61 | 0.48 | 0.117 – 0.839 | 2.59 | **0.010** |
| log(Length)*Harvest phase 1 | 0.53 | -0.64 | -1.678 – 0.406 | -1.20 | 0.231 |
| log(Length)*Recovery phase | 0.31 | -1.18 | -2.468 – 0.112 | -1.79 | 0.073 |
| log(Length)*Harvest phase 2 | 0.06 | -2.80 | -4.608 – -0.982 | -3.02 | **0.003** |
| 1. Lifetime offspring count | | | | | |
| (Intercept):  Random, Harvest phase 1 | 11.22 | 2.42 | 2.164 – 2.673 | 18.62 | **<0.0001** |
| Negative | 1.21 | 0.19 | -0.155 – 0.535 | 1.08 | 0.281 |
| Positive | 1.24 | 0.22 | -0.134 – 0.571 | 1.22 | 0.224 |
| Harvest phase 2 | 0.83 | -0.19 | -0.545 – 0.163 | -1.06 | 0.290 |
| Negative *Harvest phase 2 | 0.39 | -0.94 | -1.439 – -0.444 | -3.71 | **0.0002** |
| Positive * Harvest phase 2 | 0.57 | -0.56 | -1.049 – -0.072 | -2.25 | **0.025** |

### *4.3.5.* Lifespan

Figure S10. a) Adult, b) Reproductive, and c) Post-reproductive lifespans for males (thicker symbol stroke, only in a)) and females exposed to different harvest regimes: Negative (blue triangle), Random (circle), and Positive (orange inverted triangle) harvests.

| Table S16. Pairwise comparison between harvest regimes for adult lifespan in males and females, and reproductive and post-reproductive lifespans for females only. Estimates are in days. Degrees of freedom are 251 for adult lifespan, and 92 for reproductive and post-reproductive lifespans. | | | | |
| --- | --- | --- | --- | --- |
| Contrast | Estimate | SE | *t* ratio | *P* value |
| Adult lifespan: Females | | | | |
| Random - Negative | -33.1 | 39.6 | -0.84 | 0.404 |
| Random - Positive | -111.2 | 41.0 | -2.71 | **0.007** |
| Negative - Positive | -78.1 | 39.2 | -1.99 | **0.048** |
| Adult lifespan: Males | | | | |
| Random - Negative | 140.5 | 51.4 | 2.73 | **0.007** |
| Random - Positive | -36.4 | 51.4 | -0.71 | 0.480 |
| Negative - Positive | -176.8 | 52.4 | -3.38 | **0.001** |
| Reproductive lifespan: Females | | | | |
| Random - Negative | -13.5 | 12.3 | -1.10 | 0.275 |
| Random - Positive | -16.04 | 12.7 | -1.26 | 0.212 |
| Negative - Positive | -2.5 | 12.2 | -0.21 | 0.837 |
| Post-reproductive lifespan: Females | | | | |
| Random - Negative | -19.60 | 41.30 | -0.48 | 0.636 |
| Random - Positive | -95.10 | 42.70 | -2.23 | **0.029** |
| Negative - Positive | -75.50 | 40.90 | -1.85 | 0.068 |

# References

Amaral, I.P.G. & Johnston, I.A. (2012). Experimental selection for body size at age modifies early life-history traits and muscle gene expression in adult zebrafish. *Journal of Experimental Biology*, 215, 3895–3904.

Andersen, K.H. & Brander, K. (2009). Expected rate of fisheries-induced evolution is slow. *Proceedings of the National Academy of Sciences of the United States of America*, 106, 11657–11660.

Auer, S.K. (2010). Phenotypic plasticity in adult life‐history strategies compensates for a poor start in life in Trinidadian guppies (*Poecilia reticulata*). *The American Naturalist*, 176, 818–829.

Boukal, D.S., Dieckmann, U., Enberg, K., Heino, M. & Jørgensen, C. (2014). Life-history implications of the allometric scaling of growth. *Journal of Theoretical Biology*, 359, 199–207.

Browman, H.I., Law, R. & Marshall, T. (2008). The role of fisheries-induced evolution. *Science*, 320, 47–50; author reply 47-50.

Brown, C., Hobday, A., Ziegler, P. & Welsford, D. (2008). Darwinian fisheries science needs to consider realistic fishing pressures over evolutionary time scales. *Mar. Ecol. Prog. Ser.*, 369, 257–266.

Conover, D.O. & Munch, S.B. (2002). Sustaining fisheries yields over evolutionary time scales. *Science*, 297, 94–96.

Constantz, G.D. (1984). Sperm competition in poeciliid fishes. In: *Sperm competition and the evolution of animal mating systems* (ed. Smith, R.L.). Academic Press, Orlando, FL, pp. 465–485.

de Villemereuil P, Gaggiotti OE, Mouterde M, Till-Bottraud I. (2016). Common garden experiments in the genomic era: new perspectives and opportunities, *Heredity*. 116, 249-54.

Diaz Pauli, B. (2012). Contemporary evolution caused by fisheries. Contributions from experimental studies. PhD Thesis. University of Bergen.

Diaz Pauli, B., Garric, S., Evangelista, C., Vøllestad, L.A. & Edeline, E. (2019). Selection for small body size favours contrasting sex-specific life histories, boldness and feeding in medaka, *Oryzias latipes*. *BMC Evolutionary Biology*, 19, 127.

Diaz Pauli, B. & Heino, M. (2013). The importance of social dimension and maturation stage for the probabilistic maturation reaction norm in *Poecilia reticulata*. *Journal of Evolutionary Biology*, 26, 2184–2196.

Evangelista, C., Dupeu, J., Sandkjenn, J., Diaz Pauli, B., Herland, A., Meriguet, J., *et al.* (2021). Ecological ramifications of adaptation to size-selective mortality. *Royal Society Open Science*, 8, 210842.

Evans, J.P. & Magurran, A.E. (2000). Multiple benefits of multiple mating in guppies. *PNAS*, 97, 10074–10076.

Haldane, J.B.S. (1949). Suggestions as to Quantitative Measurement of Rates of Evolution. *Evolution*, 3, 51–56.

Heino, M., Dieckmann, U. & Godø, O.R. (2002). Measuring probabilistic reaction norms for age and size at maturation. *Evolution*, 56, 669–678.

Hendry, A.P. & Kinnison, M.T. (1999). The pace of modern life: measuring rates of contemporary microevolution. *Evolution*, 53, 1637–1653.

Hilborn, R. (2006). Faith-based fisheries. *Fisheries*, 31, 554–555.

Hilborn, R. & Minte-Vera, C.V. (2008). Fisheries-induced changes in growth rates in marine fisheries: are they significant? *Bulletin of Marine Science*, 83, 95–105.

Huxman, T. E., Winkler, D. E., & Mooney, K. A. (2022). A common garden super-experiment: An impossible dream to inspire possible synthesis. *Journal of Ecology*, 110, 997–1004.

Kallman, K.D. & Schreibman, M.P. (1973). A sex-linked gene controlling gonadotrop differentiation and its significance in determining the age of sexual maturation and size of the platyfish, \emphXiphophorus maculatus. *General and Comparative Endrocrinology*, 21, 287–304.

Kuparinen, A. & Merilä, J. (2008). The role of fisheries-induced evolution. *Science*, 320, 47–50; author reply 47-50.

Lambert, M. R., Brans, K. I., Des Roches, S., Donihue, C. M., & Diamond, S. E (2021). Adaptive Evolution in Cities: Progress and Misconceptions. *Trends in Ecology & Evolution,* 36, 239-257

López-Sepulcre, A., Gordon, S.P., Paterson, I.G., Bentzen, P. & Reznick, D.N. (2013). Beyond lifetime reproductive success: the posthumous reproductive dynamics of male Trinidadian guppies. *Proc Biol Sci*, 280, 20131116.

Minte-Vera, C.V., Maunder, M.N., Casselman, J.M. & Campana, S.E. (2016). Growth functions that incorporate the cost of reproduction. *Fisheries Research*, 180, 31–44.

Molles Jr, M.C. (2002). *Ecology: Concepts and Applications*. 2nd edn. The McGraw-Hill Companies, Inc, New York.

Olsen, E.M. & Moland, E. (2011). Fitness landscape of Atlantic cod shaped by harvest selection and natural selection. *Evolutionary Ecology*, 25, 695–710.

Pinheiro, J., Bates, D., DebRoy, S., Sarkar, D., & R Core Team. (2021). nlme: Linear and nonlinear mixed effects models. R package version 3.1-152.

Potter, T., King, L., Travis, J. & Bassar, R.D. (2019). Competitive asymmetry and local adaptation in Trinidadian guppies. *Journal of Animal Ecology*, 88, 330–342.

Quince, C., Abrams, P.A., Shuter, B.J. & Lester, N.P. (2008). Biphasic growth in fish I: theoretical foundations. *Journal of Theoretical Biology*, 254, 197–206.

Renneville, C., Millot, A., Agostini, S., Carmignac, D., Maugars, G., Dufour, S., *et al.* (2020). Unidirectional response to bidirectional selection on body size. I. Phenotypic, life-history, and endocrine responses. *Ecology and Evolution*, 10, 10571–10592.

Reznick, D.N., Bassar, R.D., Travis, J. & Helen Rodd, F. (2012). LIFE-HISTORY EVOLUTION IN GUPPIES VIII: THE DEMOGRAPHICS OF DENSITY REGULATION IN GUPPIES (POECILIA RETICULATA). *Evolution*, 66, 2903–2915.

Reznick, D.N. & Bryant, M. (2007). Comparative long-term mark-recapture studies of guppies (Poecilia reticulata): differences among high and low predation localities in growth and survival. *Annales Zoologici Fennici*.

Reznick, D.N., Butler, M.J., Rodd, F.H. & Ross, P. (1996a). Life-history evolution in guppies (Poecilia reticulata) .6. Differential mortality as a mechanism for natural selection. *Evolution*, 50, 1651–1660.

Reznick, D.N., Rodd, F.H. & Cardenas, M. (1996b). Life-History Evolution in Guppies (Poecilia reticulata: Poeciliidae). IV. Parallelism in Life-History Phenotypes. *The American Naturalist*, 147, 319–338.

Reznick, D.N. & Travis, J. (2019). Experimental Studies of Evolution and Eco-Evo Dynamics in Guppies ( *Poecilia reticulata* ). *Annu. Rev. Ecol. Evol. Syst.*, 50, 335–354.

Schreibman, M.P. & Kallman, K.D. (1977). The genetic control of the pituitary-gonadal axis in the platyfish, Xiphophorus maculatus. *Journal of Experimental Zoology*, 200, 277–293.

Schröder, A., Nilsson, K.A., van Kooten, T. & Reichstein, B. (2009). Invasion success depends on invader body size in a size-structured mixed predation-competition community. *Journal of Animal Ecology*, 78, 1152–1162.

Thompson, M.J., Réale, D., Chenet, B., Delaitre, S., Fargevieille, A., Romans, M., Caro, S. P., Charmantier, A. (2025). The city and forest bird flock together in a common garden: genetic and environmental effects drive urban phenotypic divergence, *Evolution*, 79, 800–822.

Turner, C.L. (1941a). Morphogenesis of the gonopodium in \emphGambusia affinis affinis. *Journal of Morphology*, 69, 161–185.

Turner, C.L. (1941b). Morphogenesis of the gonopodium in *Gambusia affinis affinis*. *Journal of Morphology*, 69, 161–185.

van Wijk, S.J., Taylor, M.I., Creer, S., Dreyer, C., Rodrigues, F.M., Ramnarine, I.W., *et al.* (2013). Experimental harvesting of fish populations drives genetically based shifts in body size and maturation. *Frontiers in Ecology and the Environment*, 11, 181–187.
